# Rhenium(I) Complexes with 2-(1,2,4-Triazol-5-yl)-*β*-Carboline-Based Bidentate Luminophores and Neutral Co-Ligands: Towards Tunable Phosphorescence and Efficient Singlet Dioxygen Photoproduction

**DOI:** 10.3390/ijms262110349

**Published:** 2025-10-24

**Authors:** Joschua Lüke, Iván Maisuls, Alexander Hepp, Cristian A. Strassert

**Affiliations:** 1 Institut für Anorganische und Analytische Chemie, Universität Münster, Corrensstraße 28/30, 48149 Münster, Germany; 2 CiMIC, SoN, CeNTech, Universität Münster, Heisenbergstraße 11, 48149 Münster, Germany

**Keywords:** *β*-carboline alkaloids, rhenium(I) complexes, bidentate ligands, photoluminescence, phosphorescence, photoproduction of singlet dioxygen, photosensitizer, photosensitization

## Abstract

A bidentate ligand concept based on *β*-carbolines functionalized with a 1,2,4-triazolyl-moiety was designed and realized, enabling the development of a series of neutral rhenium(I) complexes. This new class of anionic ligands, incorporating either an unsubstituted 9*H*-pyrido[3,4-*b*]indole core (**L_nHo_**) or a 9-methyl-substitued variant (**L_Me-nHo_**), was developed towards tailored photofunctionality. Structural modification via methyl substitution at the indole moiety was found to enhance overall phosphorescence efficiency. Comparative studies of two monodentate auxiliary units revealed that 1,3,5-triaza-7-phosphaadamantane (**PTA**) significantly reduces the photoluminescence efficiency compared to pyridine (**Py**). Solvent-dependent photoluminescence studies indicated that a lowered polarity leads to an increase in photoluminescence quantum yields (*Φ*_L_). The complex **Re(L_Me__-nHo_)Py** emerged as the most efficient emitter, displaying a *Φ*_L_ of 44% in dichloromethane (DCM). Notably, all complexes exhibited efficient quenching of excited triplet states by diffusional collision with triplet dioxygen (^3^O_2_), yielding good singlet dioxygen (^1^O_2_) photoproduction efficiencies (*Φ_Δ_*) with a maximum of 45% observed for **Re(L_nHo_)Py**. These results highlight the suitability of these complexes for applications requiring efficient phosphorescence and oxygen photosensitization, such as bioimaging, and photodynamic therapy or photooxidation catalysis, while underscoring the central role of the tailored *β*-carboline-based chromoluminophores in enabling precise tuneability of photophysical properties.

## 1. Introduction

**Scheme 1:**
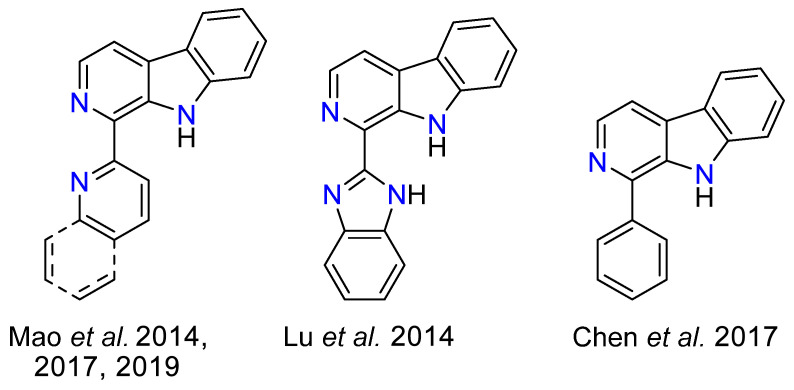
Bidentate ligand precursors incorporating a *β*-carboline developed by Mao et al. [32] for Ir(III), Re(I) [33], and Ru(II) [34] complexes (**left**); by Lu et al. [35] for Pt(II), Ni(II), Cu(II), and Co(II) complexes (**center**); by Chen et al. [36] for Ru(II) complexes (**right**).

Photoactive transition metal complexes have attracted significant attention due to their diverse applications in many fields. Among these, rhenium(I) complexes stand out for their robust coordination chemistry, well-defined molecular structures, and remarkable photophysical properties [1]. Owing to their exceptional versatility, rhenium(I) complexes are commonly employed as metallodrugs [2,3,4,5], in OLEDs [6,7,8], as photosensitizers [9,10,11] and as photocatalysts [12,13,14]. Among them, rhenium(I) tricarbonyl complexes have been predominantly investigated with bidentate chelating ligands derived from *α*,*α*-diimines (particularly 1,10-phenantroline and 2,2′-bipyridine), as result of their well-defined coordination geometry and synthetic accessibility [15,16,17,18,19,20,21]. These complexes exhibit photoexcited states with predominantly metal-to-ligand charge-transfer (MLCT, *d*-π*), ligand-to-ligand charge-transfer (LLCT), or ligand-centered (LC, *π*-π*) character, often occurring as admixtures thereof [22]. However, there is a growing interest in incorporating heterocyclic five-membered rings, such as triazoles, as ligand components to expand the tunability of the electronic structure and photophysical response of metal complexes. In this context, the triazole ring, with its potentially anionic nitrogen coordination sites and a delocalized *π*-electronic system, offers enhanced synthetic versatility and opportunities for fine-tuning luminescent properties [23]. Similarly, *β*-carbolines represent another class of heterocyclic ligands characterized by a rigid, *π*-conjugated framework combined with strong donor functionalities [24,25,26,27,28,29,30,31]. While *β*-carbolines have been predominantly investigated as monodentate ligands for their photophysical and biological properties, their potential as bidentate luminophores remains underexplored, with only few examples present on the literature (Scheme 1) [32,33,34,35,36]. The fusion of *β*-carboline scaffolds with triazolyl moieties offers an opportunity to develop novel ligand platforms with tailored electronic and photophysical characteristics.

In this work, as part of our efforts to further explore the photophysical properties of bidentate *β*-carboline-based systems, we introduce a monoanionic bidentate ligand based on 2-[1,2,4-triazol-5-yl]-*β*-carboline. This ligand combines the strong *σ*-donor properties of the deprotonated triazolyl unit with the extended *π*-conjugation of the *β*-carboline core, enabling the formation of neutral rhenium(I) tricarbonyl complexes. Additionally, the effect of methyl substitution at the pyrrole moiety was investigated to modulate its photophysical characteristics. Furthermore, the influence of different monodentate auxiliary units, either pyridine (**Py**) or 1,3,5-triaza-7-phosphaadamantane (**PTA**), on photoluminescence and singlet dioxygen photogeneration efficiency was also evaluated.

The resulting neutral rhenium(I) complexes were fully characterized, including structural analysis by infrared (IR) and two-dimensional nuclear magnetic resonance spectroscopy (2D-NMR), as well as by exact mass spectrometry (EM-MS). Their photophysical properties were systematically investigated through UV–vis absorption, steady-state and time-resolved photoluminescence spectroscopies, as well as photoluminescence quantum yield measurements in two different solvents. Additionally, the ability of the complexes to photosensitize molecular oxygen was assessed by quantifying the photoproduction of singlet dioxygen.

## 2. Results and Discussion

### 2.1. Synthesis and Structural Characterization of the Complexes

**Scheme 2:**
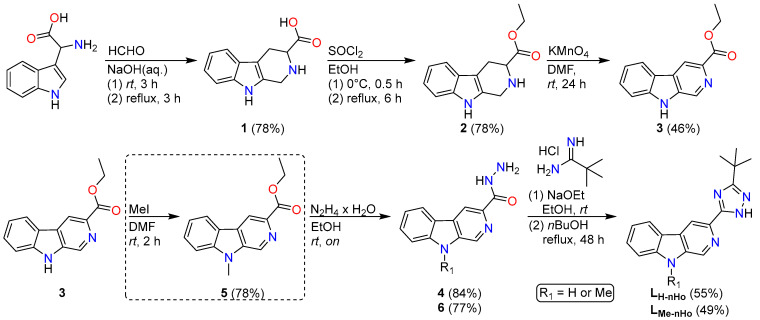
Synthetic route towards the *β*-carboline-derived chelators. The additional reaction step for the introduction of the methyl group in **L_Me-nHo_** is shown in a dashed box, as it is not required for the synthesis of **L_nHo_**.

The synthesis of the two ligand precursors (Scheme 2), namely 2-[3-(*tert*-butyl)-1*H*-1,2,4-triazol-5-yl]-*β*-carboline (**L_nHo_**) and 2-[3-(*tert*-butyl)-1*H*-1,2,4-triazol-5-yl]-6-methyl-*β*-carboline (**L_Me-nHo_**), was carried out by following a previously reported procedure up to the complete formation of the *β*-carboline core [35]. Subsequently, the 1,2,4-triazole moiety was introduced via a two-step approach [37]: first, formation of a formyl hydrazide intermediate using hydrazine monohydrate, followed by cyclocondensation with pivalamidine hydrochloride.

**Scheme 3:**
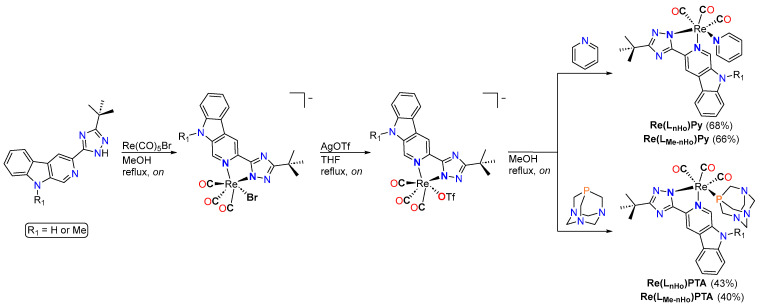
Synthetic route towards the rhenium(I) complexes.

The rhenium complexes **Re(L_nHo_)Py**, **Re(L_nHo_)PTA**, **Re(L_Me-nHo_)Py**, and **Re(L_Me-nHo_)PTA** were synthesized through a three-step route (Scheme 3). Initially, an Ar-purged methanol solution of Re(CO)_5_Br (0.16 mmol) was treated with an equimolar amount of the corresponding bidentate ligand precursor (**L_nHo_** or **L_Me-nHo_**) and refluxed overnight under Ar. After filtration, the resulting intermediate complex (Scheme 3) was reacted with an equimolar amount of silver trifluoromethanesulfonate (AgOTf) in methanol and refluxed to facilitate bromide abstraction. The resulting AgBr precipitate was removed by filtration, and the solvent was evaporated under reduced pressure.

The resulting triflato-containing complexes were then redissolved in methanol and treated with an equimolar amount of the desired monodentate co-ligand, either 1,3,5-triaza-7-phosphoadamantane (**PTA**) or pyridine (**Py**), and the mixture was refluxed overnight to ensure complete ligand exchange. Final purification was achieved by silica gel column chromatography (DCM/MeOH 98:2), affording the desired rhenium complexes in good purity.

Both chromoluminophoric chelators and the four complexes were comprehensively characterized by electrospray ionization mass spectrometry (ESI-MS) as well as by one- and two-dimensional NMR spectroscopies (^1^H and ^13^C), allowing unambiguous assignment of all observed signals (Figures S1–S62). Detailed experimental procedures, including synthetic protocols, structural characterization of all ligand precursors and complexes, along with the results from the photophysical measurements, are provided in the Supplementary Materials. In addition, the structural characterization was further supported by IR spectroscopy of the four complexes (Figures S63–S66). In all cases, the characteristic CO stretching vibrations are observed in the range of 1850–2050 cm^−1^. Notably, the Re(I) complexes bearing **PTA** display three distinct CO-related stretching bands, while those with **Py** units show only two prominent bands. This difference may arise from the greater steric demand of **PTA**, which enforces a more prominently asymmetric coordination environment. In contrast, the **Py**-modified complexes give rise to a simpler IR pattern, consistent with a rather rigid and symmetric ligand arrangement around the metal center.

### 2.2. Photophysical Characterization

All complexes were characterized in terms of their photophysical properties, including UV–vis absorption and photoluminescence emission spectroscopy, in acetonitrile (ACN) and dichloromethane (DCM) solutions, which were chosen as representative high- and low-polarity solvents, respectively, to evaluate the effect of the dielectric constant on the excited states. Emission spectra, excited-state lifetimes (*τ*), and photoluminescence quantum yields (*Φ*_L_) were measured both under aerated and deoxygenated conditions. Low-temperature measurements were carried out in frozen glassy matrices of a 1:1 DCM/MeOH mixture at 77 K.

**Figure 1:**
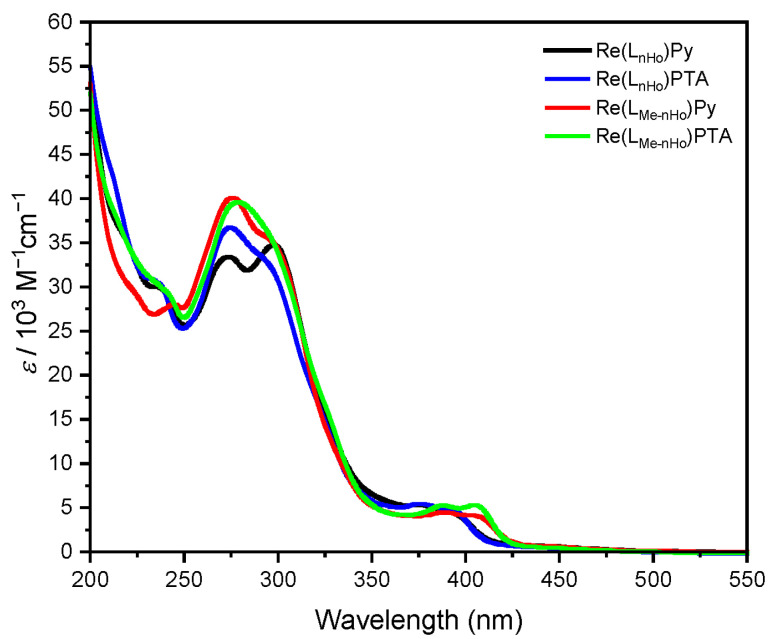
Absorption spectra (molar absorption coefficients as a function of wavelength) of **Re(L_nHo_)Py** (black), **Re(L_nHo_)PTA** (blue), **Re(L_Me-nHo_)Py** (red) and **Re(L_Me-nHo_)PTA** (green). Validity range: *c* = 1 × 10^−5^–1 × 10^−6^ M in ACN at 298 K.

The UV–vis absorption spectra of all complexes in ACN (Figure 1) exhibit two distinct bands: one in the UV region and another in the visible range. Based on previous reports concerning Re(I) complexes, the intense absorption bands between 250 nm and 325 nm observed for both ligand types are attributed to transitions into ligand-centered singlet states (^1^LC) with *π*-π* character [28,38,39]. The lower-energy bands between 350 nm and 425 nm correspond to transitions into metal-to-ligand charge-transfer singlet states (^1^MLCT). Notably, variation in the auxiliary ligand has minimal influence on the absorption energies. In contrast, methyl substitution at the pyrrole unit of the bidentate ligand induces a bathochromic shift in the MLCT-related band without notably altering its intensity. This behavior is consistent with previous observations in related Re(I) complexes incorporating *β*-carboline units as the auxiliary units [40,41].

**Figure 2:**
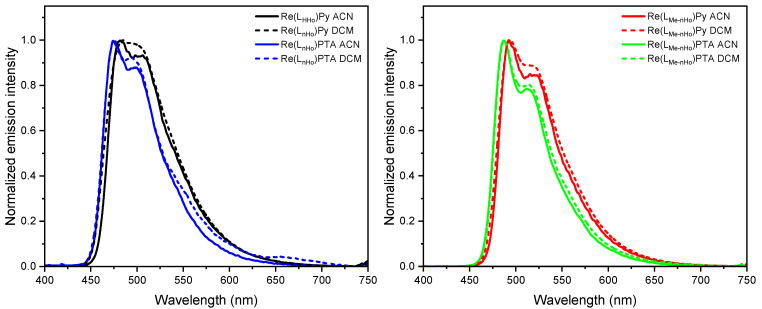
Photoluminescence emission spectra of **Re(L_nHo_)Py** (black), **Re(L_nHo_)PTA** (blue), **Re(L_Me-nHo_)Py** (red) and **Re(L_Me-nHo_)PTA** (green), recorded in different solvents and conditions (*c* = 10^−5^ M, *λ*_exc_ = 314–360 nm). Left: Emission spectra of **Re(L_nHo_)Py** and **Re(L_nHo_)PTA** in ACN (solid lines) and DCM (dotted lines) at 298 K. Right: Emission spectra of **Re(L_Me-nHo_)Py** and **Re(L_Me-nHo_)PTA** in ACN (solid lines) and DCM (dotted lines) at 298 K.

**Figure 3:**
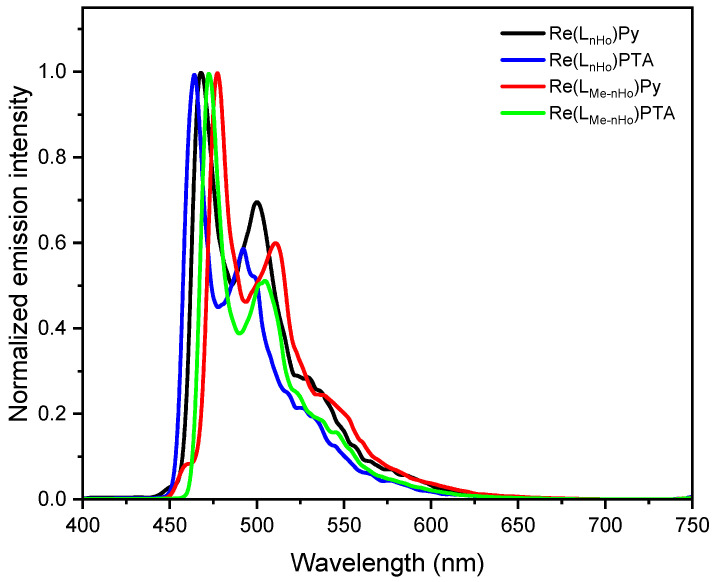
Photoluminescence emission spectra of **Re(L_nHo_)Py** (black), **Re(L_nHo_)PTA** (blue), **Re(L_Me-nHo_)Py** (red), and **Re(L_Me-nHo_)PTA** (green) recorded in a frozen glassy matrix of DCM/MeOH (V:V = 1:1) at 77 K (*λ*_exc_ = 320 nm, *c* = 10^−7^ M).

All complexes display intense photoluminescence at room temperature, characterized by broad yet partially structured emission bands, predominantly attributed to metal-to-ligand charge-transfer triplet states (^3^MLCT) with admixtures of ^3^LC (i.e., ligand-centered) character [28,42,43]. As shown in Figure 2 and Figure S67, the dielectric constant exerts minimal influence on the emission maxima, indicating relatively weak solvent-excited state interactions. However, the red-shift observed at increased polarities (i.e., when going from DCM to ACN) confirms the charge-transfer (CT) character of the emissive states. Moreover, the introduction of a methyl group at the pyrrole unit of the bidentate ligand leads to bathochromic shifts in the emission spectra: specifically, the emission maxima are red-shifted to 492 nm for **Re(L_Me-nHo_)Py** (Δ*λ*_Em_ = 10 nm; Δ*E*_Em_ = 52 meV) and to 487 nm for **Re(L_Me-nHo_)PTA** (Δ*λ*_Em_ = 12 nm; Δ*E*_Em_ = 64 meV), compared with their non-methylated analogs. The more structured emission profiles of the methyl-decorated compounds suggest a reduced ^3^MLCT character in the emissive excited state (i.e., increased ^3^LC character), further supported by longer excited-state lifetimes (*τ*, see Table 1) [28]. This bathochromic shift with an enhanced ^3^LC character points to a destabilization of occupied *π*-orbitals, lowering the energy of the *π*-*π* * configurations. Notably, replacement of **PTA** by **Py** also causes a mild red-shift with partial loss of vibronic resolution, indicating an increased ^3^MLCT character upon insertion of the **Py** coligand. In frozen glassy matrices at 77 K, the blue-shifted emission spectra display pronounced vibronic progressions, which is typically associated with reduced stabilization of the charge-transfer (CT) excited states due to suppressed solvent relaxation in a rigid environment. Thus, these spectral features point toward an increased contribution from ligand-centered (^3^LC) configurations and a diminished ^3^MLCT character, in line with observations for related Re(I) complexes (see Figure 3) [28]. Also at 77 K, an additive redshift is consistently observable upon methylation of the main ligand and insertion of **Py** as the ancillary unit.

As summarized in Table 1, comparison of the four complexes in terms of their excited-state lifetimes and photoluminescence quantum yields highlights the influence of the ligand environment (i.e., the first coordination sphere) and the solvent (i.e., the second coordination sphere) on the nature of the excited states and their deactivation pathways, consistent with previous reports on comparable Re(I) complexes [38,40,44,45,46].

**Table 1:** Photophysical properties of the rhenium(I) complexes (*c* = 10^−7^ M) in liquid ACN (top) or DCM (center) at RT (*c* = 10^−5^ M) and a glassy frozen matrix of DCM/MeOH at 77 K (bottom).

Complex	*τ* (Air) ^a^/µs	*τ* (Ar) ^a^/µs	*Φ*_L_ (Air)/%	*Φ*_L_ (Ar)/%	*k*_r_^b^/10^4^ s^−1^	*k*_nr_^b^/10^4^ s^−1^
ACN						
**Re(L_nHo_)Py**	***τ*_av___amp_** = 0.0690 ± 0.0001 [*τ*_1_ = 0.011 ± 0.002 (14%)] [*τ*_2_ = 0.0785 ± 0.0002 (51%)]	***τ*_av___amp_** = 13.77 ± 0.09 [*τ*_1_ = 15.30 ± 0.01 (89%)] [*τ*_2_ = 1.2 ± 0.1 (11%)]	<2	14 ± 2	1.0 ± 0.1	0.6 ± 0.1
**Re(L_nHo_)PTA**	***τ*_av___amp_** = 0.038 ± 0.001 [*τ*_1_ = 0.074 ± 0.001 (49%)] [*τ*_2_ = 0.0030 ± 0.0002 (51%)]	2.084 ± 0.003	<2	<2	<0.95	47.02–47.98
**Re(L_Me-nHo_)Py**	***τ*_av___amp_** = 0.059 ± 0.001 [*τ*_1_ = 0.093 ± 0.001 (58%)] [*τ*_2_ = 0.0114 ± 0.0007 (42%)]	54.02 ± 0.07	<2	26 ± 3	0.48 ± 0.04	1.37 ± 0.04
**Re(L_Me-nHo_)PTA**	***τ*_av___amp_** = 0.037 ± 0.001 [*τ*_1_ = 0.0859 ± 0.0002 (39%)] [*τ*_2_ = 0.0048 ± 0.0002 (61%)]	12.458 ± 0.005	<2	10 ± 2	0.8 ± 0.2	7.2 ± 0.2
DCM						
**Re(L_nHo_)Py**	***τ*_av___amp_** = 0.484 ± 0.001 [*τ*_1_ = 0.770 ± 0.004 (49%)] [*τ*_2_ = 0.210 ± 0.003 (51%)]	***τ*_av___amp_** = 10.85 ± 0.03 [*τ*_1_ = 11.45 ± 0.03 (92%)] [*τ*_2_ = 4.2 ± 0.4 (8%)]	<2	26 ± 3	2.3 ± 0.2	6.8 ± 0.2
**Re(L_nHo_)PTA**	***τ*_av___amp_** = 0.357 ± 0.001 [*τ*_1_ = 0.554 ± 0.004 (49%)] [*τ*_2_ = 0.167 ± 0.006 (51%)]	***τ*_av___amp_** = 3.46 ± 0.01 [*τ*_1_ = 6.4 ± 0.3 (13%)] [*τ*_2_ = 3.04 ± 0.05 (87%)]	<2	6 ± 2	1.7 ± 0.6	27.5 ± 0.6
**Re(L_Me-nHo_)Py**	***τ*_av___amp_** = 0.612 ± 0.004 [*τ*_1_ = 0.776 ± 0.003 (72%)] [*τ*_2_ = 0.20 ± 0.01 (28%)]	***τ*_av___amp_** = 38.4 ± 0.2 [*τ*_1_ = 42.08 ± 0.04 (91%)] [*τ*_2_ = 2.8 ± 0.3 (9%)]	<2	43 ± 4	1.11 ± 0.06	1.48 ± 0.05
**Re(L_Me-nHo_)PTA**	***τ*_av___amp_** = 0.284 ± 0.001 [*τ*_1_ = 0.686 ± 0.006 (12%)] [*τ*_2_ = 0.227 ± 0.002 (88%)]	***τ*_av___amp_** = 28.12 ± 0.05 [*τ*_1_ = 31.8 ± 0.3 (71%)] [*τ*_2_ = 19.1 ± 0.9 (29%)]	<2	22 ± 2	0.78 ± 0.07	2.77 ± 0.07
77 K (DCM/MeOH)						
	***τ*** **^a^** **/µs**		***Φ*_L_** **/%**		***k*** **_r_** **^b^** **/10^4^ s^−1^**	***k*** **_nr_** **^b^** **/10^4^ s^−1^**
**Re(L_nHo_)Py**	***τ*_av___amp_** = 83.6 ± 0.2 [*τ*_1_ = 114 ± 3 (44%)] [*τ*_2_ = 60 ± 2 (56%)]		95 ± 5		1.13 ± 0.03	0.06 ± 0.02
**Re(L_nHo_)PTA**	***τ*_av___amp_** = 141.7 ± 0.2 [*τ*_1_ = 195 ± 2 (44%)] [*τ*_2_ = 99 ± 1 (56%)]		95 ± 5		0.67 ± 0.02	0.04 ± 0.01
**Re(L_Me-nHo_)Py**	***τ*_av___amp_** = 111.4 ± 0.2 [*τ*_1_ = 149 ± 2 (41%)] [*τ*_2_ = 86 ± 2 (59%)]		95 ± 5		0.85 ± 0.02	0.04 ± 0.02
**Re(L_Me-nHo_)PTA**	***τ*_av___amp_** = 171.6 ± 0.3 [*τ*_1_ = 213 ± 2 (54%)] [*τ*_2_ = 124 ± 3 (46%)]		95 ± 5		0.55 ± 0.01	0.03 ± 0.01

^a^ For the multiexponential photoluminescence decays, the amplitude-weighted average lifetimes (*τ*_av_amp_) are shown, along with the individual decay components [*τ*_n_] and their corresponding amplitudes (in parentheses). ^b^ For multiexponential lifetimes, *τ*_av_amp_ was used to estimate average *k*_r_ and *k*_nr_ values [47]. Raw time-resolved photoluminescence decays and fitting parameters are shown in the Supplementary Materials Figures S68–S87. *k*_r_ and *k*_nr_ were estimated only for the Ar-purged solutions. A more extended table can be found in Table S1 (Section S4).

Assuming unitary intersystem crossing efficiencies (due to chelation of a late transition metal) [9], the average radiative and radiationless deactivation rate constants (*k*_r_ and *k*_nr_, respectively) were estimated according to the following equations and relationships:

(1) $$k_{r}=\;\frac{\Phi_{L}}{\tau_{L}}$$

(2) $$k_{nr}=\frac{1-\Phi_{L}}{\tau_{L}}$$

(3) $$\tau_{L}=\frac{1}{k_{r}+{\;k}_{nr}}$$

where *τ*_L_ is the excited-state lifetime (or amplitude-weighted average lifetime, *τ*_av_amp_ for multiexponential decays) [47] determined by time-resolved photoluminescence spectroscopy, and *Φ*_L_ is the absolute photoluminescence quantum yield. All the quantum yields, excited-state lifetimes, as well as the calculated radiative (*k*_r_) and non-radiative (*k*_nr_) rate constants are summarized in Table 1.

In deoxygenated ACN, **Re(L_nHo_)Py** exhibits a photoluminescence lifetime of 13.77 µs. In contrast, with the **PTA** ligand (**Re(L_nHo_)PTA**) a significant quenching of the excited state occurs, with the lifetime decreasing to 2.08 µs compared to **Re(L_nHo_)Py**. This effect can be attributed to the **PTA** ligand’s nitrogen lone pairs facilitating non-radiative decay of the excited triplet state by partially quenching the MLCT states [48]. A similar trend is observed upon co-ligand exchange on the methyl-substituted analogues, although the overall lifetimes are considerably longer for the methylated species. Specifically **Re(L_Me-nHo_)Py** shows a *τ* of 54.03 µs, while **Re(L_Me-nHo_)PTA** exhibits a shorter, yet still extended *τ* of 12.46 µs. The pronounced increase in lifetime upon methyl substitution likely arises from the enhanced ^3^LC character deriving from the destabilization of occupied *π*-orbitals while lowering the energy of the emissive state [40].

Interestingly, the methylated complexes exhibit a higher *Φ*_L_ compared to their non-methylated counterparts (Table 1). While both **PTA**-containing complexes display relatively low *Φ*_L_ values (2% for **Re(L_nHo_)PTA** and 10% for **Re(L_Me-nHo_)PTA**), the pyridine-containing analogues exhibit significantly enhanced efficiencies, reaching *Φ*_L_ values of 14% and 26% for **Re(L_nHo_)Py** and **Re(L_Me-nHo_)Py**, respectively. These results highlight the favorable photophysical properties imparted by the pyridine ligand in combination with the electron-donating methyl substituent. As previously discussed, this behavior can be rationalized by the ability of the **PTA** ligand to quench the ^3^MLCT states through the lone pairs of its nitrogen atoms, which facilitates non-radiative decay pathways [48].

When studied in the less polar solvent DCM instead of ACN, the complexes display a distinct profile. The lifetimes of the **Py**-decorated complexes slightly decrease, whereas those of the **PTA**-containing analogues moderately increase. Moreover, the **Py**-containing systems (characterized by a more pronounced ^3^MLCT nature as compared to their **PTA**-bearing analogs, *vide supra*), experience a mild destabilization of the excited state in DCM. As shown in Table 1, despite the increase in lifetime for the **PTA**-containing complexes in DCM compared with ACN, their lifetimes remain considerably shorter than those of their **Py**-decorated analogues, indicating that quenching by **PTA** persists, albeit less efficiently in this solvent. Interestingly, in DCM, the *Φ*_L_ are enhanced compared with those measured in ACN, yet the same general trend is observed for all coordination compounds: **Py**-containing complexes outperform their **PTA** counterparts, and methylated luminophores (**L_Me-nHo_**) consistently surpass the performance of the non-methylated ones (**L_nHo_**).

Analysis of *k*_r_ and *k*_nr_ values in both solvents provided further insights (Table 1). First, methylation of the nHo chelator (**L_Me-nHo_**) leads to a decrease in *k*_r_ relative to the non-methylated analogues (**L_nHo_**), likely reflecting the slight increase in the ^3^LC character mentioned above (*vide supra*). At the same time, the **PTA** ligand, due to the quenching mechanism described above, significantly increases *k*_nr_ when compared to the corresponding **Py**-based complexes. Moreover, the enhanced ^3^MLCT character related to **Py** appears to enhance the *k*_r_ values. However, given the decisive yet intricate solvent-complex interactions (including solvent–monodentate co-ligand (**PTA** or **Py**), solvent–metal center (i.e., Re^I^(CO)_3_ core), solvent-luminophoric chelator (**L_nHo_** or **L_Me-nHo_**), chelator-co-ligand, metal center-chelator, and co-ligand-metal center interactions), no unambiguous trend can be established regarding *k*_nr_ values and the coordination spheres (i.e., methylation of the luminophoric chelator, exchange of the co-ligand, and the dielectric constant of the environment). For this reason, we also performed measurements at 77 K, where the solvent is frozen, and its influence is essentially negligible.

Within a glassy matrix at 77 K, all complexes exhibit time-resolved photoluminescence decays with amplitude-weighted average lifetimes ranging from 84 to 172 μs (Table 1). These markedly prolonged lifetimes, compared to those measured at room temperature in liquid solutions, reflect the suppression of non-radiative deactivation pathways at lower temperatures due to restricted molecular motion and solvent relaxation. This effect is also evident in the *Φ*_L_ values, which approach unity for all four complexes under these conditions. Moreover, the calculated *k*_nr_ values confirm the efficient suppression of radiationless deactivation pathways.

**Figure 4:**
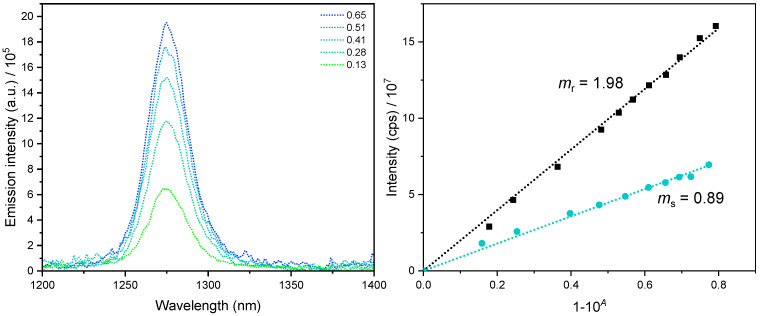
Singlet dioxygen phosphorescence exemplarily shown for **Re(L_nHo_)Py** in ACN. **Left**: ^1^O_2_ phosphorescence spectra at different concentrations of the complex. **Right**: ^1^O_2_ phosphorescence intensity versus the fraction of light absorbed (i.e., 1–10^−*A*^) for **Re(L_nHo_)Py** (cyan) and the reference (black). *l*_ex_ = 350 nm. *m*_s_ and *m*_r_ are the slopes of the sample and reference, respectively. For the other complexes, see Figures S88−S91.

With non-radiative processes essentially negligible at 77 K, the influence of methyl substitution at the nHo unit becomes clearly visible on *k*_r_. Specifically, when comparing the complexes bearing the same monodentate ligand (**PTA** or **Py**), methylation of the nHo moiety consistently decreases *k*_r_, in line with the reasons discussed above (i.e., reduced ^3^MLCT character). In addition, the **PTA** ligand moderately reduces *k*_r_, likely due to a reduced ^3^MLCT character. Consequently, as shown in Table 1, the highest *k*_r_ is observed for **Re(L_nHo_)Py**, while the lowest corresponds to **Re(L_Me-nHo_)PTA**. Due to the frozen second coordination sphere at 77 K, the emission likely arises from triplet states with more ^3^LC and less ^3^MLCT character. This shift is also reflected in the emission spectra (Figure 4), which display a rather structured vibronic progression characteristic of ligand-centered excited states [49].

### 2.3. Photogeneration of ^1^O_2_

Given the ability of these complexes to populate long-lived triplet excited states and their potential to photogenerate singlet dioxygen (^1^O_2_), their interaction with triplet dioxygen (^3^O_2_) was further investigated [11]. In particular, differences in oxygen sensitivity among the complexes provide insights into their quenching dynamics and photosensitizing capabilities.

The efficiency of diffusion-controlled quenching of the photoexcited triplet state of the complexes by ^3^O_2_ was assessed via singlet dioxygen photoproduction quantum yields (*Φ_Δ_*). The determination of ^1^O_2_ generation efficiency involved an analysis of the quenching interaction between the triplet excited states of the photosensitizer and dissolved ^3^O_2_. This bimolecular process is governed by the total quenching rate constant (*k*_q_) which quantifies the essentially diffusion-limited deactivation of the triplet state by ^3^O_2_.

In this context, $P_{O_{2}}^{T}$ represents the fraction of triplet states that are deactivated by ^3^O_2_ under the experimental conditions while providing a measure of the overall quenching efficiency, whereas $f_{O_{2}}^{T}$ represents the fraction of quenching events that actually lead to the formation of ^1^O_2_. The employed equations and methodological details are provided in the Supplementary Materials. A summary of the data described herein is compiled in Table 2.

With a *Φ*_L_ of 26% in Ar-purged and <2% in air-equilibrated ACN solutions, the **Re(L_Me__-nHo_)Py** complex appears to be the most sensitive species regarding quenching by ^3^O_2_. Its singlet dioxygen generation efficiency reached 41%, which is a slightly lower than that of **Re(L_nHo_)Py** attaining 45% as the highest *Φ_Δ_* value observed in the series (representative data shown in Figure 4). Considering the experimental uncertainty, both complexes can be regarded as having comparable *Φ_Δ_* values, suggesting that methyl substitution at the indole-moiety does not significantly affect the efficiency of oxygen photosensitization.

Values up to 29% and 39% were recorded for **Re(L_nHo_)PTA** and **Re(L_Me-nHo_)PTA**, respectively. Overall, the obtained *Φ_Δ_* values are in agreement with previously reported data [11,27,50], although comparable studies on Re(I) complexes bearing monoanionic bidentate ligand systems remain scarce [28,40]. Slightly higher efficiencies are generally observed for the complexes incorporating pyridine as an auxiliary ligand (i.e., for the species with more ^3^MLCT character in the excited state, *vide supra*), whereas **Re(L_nHo_)PTA** stands out with a notably lower singlet dioxygen generation efficiency. This diminished performance can likely be attributed to the quenching pathway previously discussed for **PTA**-containing complexes, as well to a higher excited state ^3^LC character. As shown in Table 1, the difference between *τ*_air_ and *τ*_Ar_ is significantly larger for the **Py**-containing complexes than for those bearing **PTA**, indicating a higher fraction of triplet states effectively quenched by triplet dioxygen in the former. This supports the interpretation that the **PTA** ligand promotes non-radiative deactivation pathways that compete with ^1^O_2_ sensitization.

From the experimental *τ*_air_ and *τ*_Ar_ values (Table 1), the parameter $P_{O_{2}}^{T}$ was determined. Due to the very short excited-state lifetimes in air-equilibrated ACN solutions, nearly complete quenching of the triplet states by triplet dioxygen is evident for all compounds. Only **Re(L_nHo_)PTA** again shows a slightly reduced efficiency in this regard. As $P_{O_{2}}^{T}$ approaches 100% for all systems, the fraction of triplet states quenched by ^3^O_2_ that results into ^1^O_2_ generation ($f_{O_{2}}^{T}$) are nearly identical to the values of singlet dioxygen photoproduction. A marginal deviation is observed for **Re(L_nHo_)PTA**, which exhibits a somewhat smaller $f_{O_{2}}^{T}$, consistent with its overall lower sensitization efficiency *Φ_Δ_*.

The fraction of quenching events leading to ^1^O_2_ (herein denoted as $f_{O_{2}}^{T}$) from an exciplex between the excited metal complex (^3^M) and ^3^O_2_ can only reach unity if relaxation occurs exclusively via a singlet pathway (this is usually the case if spin statistics are severely perturbed, *vide infra*):


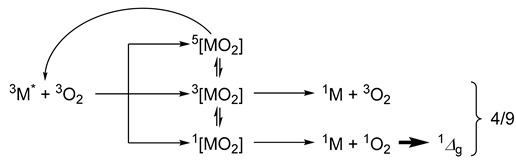


According to Hund’s rule, the dissociative quintet exciplex (^5^[MO_2_]) is the most stable spin state. However, it undergoes reversible decay and does not directly participate in excited state quenching or singlet dioxygen generation. Thus, rapid intersystem crossing (ISC) from ^3^[MO_2_] to the more stable ^5^[MO_2_] state can deplete the population of triplet exciplexes, while reducing their contribution to overall quenching. In contrast, the singlet exciplex pathway could remain largely unaffected, as the larger singlet–triplet energy gap can hamper ISC from the ^1^[MO_2_] species. However, if the quenching process is exclusively limited to the singlet path (i.e., with ISC channeling only a fraction of the singlet encounter complexes yet all triplet exciplexes towards dissociative quintets), the quenching rate constant is limited to *k*_q_ ≤ *k*_d_/9 (where *k*_d_ is the diffusion rate of triplet dioxygen). Under these conditions, even though *k*_q_ could remain limited (i.e., *k*_q_ ≤ *k*_d_/9), the singlet dioxygen generation efficiency $f_{O_{2}}^{T}$ can still approach unity, since all productive quenching events proceed through the singlet channel [9,51]. In contrast, under diffusion-controlled conditions where singlet, triplet and quintet exciplexes are formed with spin-statistical probability (i.e., with negligible ISC between them), the quenching rate approximates to *k*_q_ ≈ 4*k*_d_/9. In this case, the maximum attainable efficiency would drop to $f_{O_{2}}^{T}$ = 0.25, since only the singlet exciplexes contribute to ^1^O_2_ generation (whereas the triplet exciplexes do not, despite contributing to overall quenching) [1]. In general, smaller than expected values of $f_{O_{2}}^{T}$ (i.e., <1 or <0.25 with fast or negligible ISC, respectively) would point to non-productive relaxation paths affecting the singlet exciplexes.

The theoretical considerations discussed above can be contrasted with the actual values obtained: While the highest attainable quenching rate would be *k*_q_ ≈ 4*k*_d_/9 (due to diffusion-limited encounters with *k*_d_ ≈ 30 × 10^9^ M^−1^s^−1^) [52,53], the relationship of *k*_q_ ≤ *k*_d_/9 is confirmed herein, with values that range from 1.4 to 2.7 × 10^9^ M^−1^s^−1^. Moreover, the observation that $f_{O_{2}}^{T}$ exceeds 0.25 (yet still below 1, i.e., ranging between 0.30 and 0.40) further indicates that quenching through the triplet pathway is at least partially competitive [54]. This is likely due to strong spin–orbit coupling (and/or charge-transfer character of the exciplex), which alters the exciplexes’ spin state distributions and facilitates ISC within Dexter-type energy transfer [11]. Thus, the *k*_q_ and $f_{O_{2}}^{T}$ values are consistent with a triplet metal-to-ligand charge-transfer (^3^MLCT) excited states and agree with data reported for related Re(I) tricarbonyl complexes [11,28]. These findings highlight the favorable photophysical dynamics of the complexes and reinforce their potential as singlet dioxygen photosensitizers.

## 3. Materials and Methods

General information regarding synthesis and characterization is found in Section 2. Further details regarding materials, synthetic procedures, purification and structural characterization are found in the Supplementary Materials Sections S1–S3, along with an extended description of the photophysical characterization methods and techniques as well as detailed photophysical data.

## 4. Conclusions

In summary, a new class of monoanionic 2-(1,2,4-triazol-5-yl)*-β*-carboline-based ligands as bidentate chelators was developed and effectively incorporated into a series of neutral rhenium(I) complexes. Structural modification through methyl substitution at the pyrrole moiety of the complexes caused a red-shift in the emission while enhancing the photoluminescence efficiencies. Hence, the bathochromic effect is attributed to the stabilization of the emissive ^3^LC configurations. Furthermore, a comparative analysis of two monodentate auxiliary ligands, **Py** and **PTA**, revealed that the presence of **PTA** consistently reduced emission efficiencies: this is likely due to an enhanced non-radiative deactivation via its lone pairs at the nitrogen atoms. However, the radiative rates are improved with **Py** as the participation of the Re(I) center on the excited state is increased. In addition, the influence of the dielectric constant on the emission properties were investigated and it was found that *Φ*_L_ was somewhat reduced in more polar solvents. Among all complexes, **Re(L_Me__-nHo_)Py** emerged as the most efficient emitter, exhibiting a *Φ*_L_ of 43% in liquid DCM at room temperature with a relatively long excited triplet state lifetime reaching roughly 40 µs. Time-resolved and steady-state measurements also revealed efficient interaction of all complexes with ^3^O_2_, enabled by population of long-lived triplet states and facilitating ^1^O_2_ generation. In particular, **Re(L_nHo_)Py** achieved the highest singlet dioxygen photoproduction quantum yield (*Φ_Δ_* = 45%), highlighting its potential as a photosensitizer. Overall, this study demonstrates that rational design strategies, based on targeted substitution of bidentate frameworks and judicious choice of monodentate ligands, can effectively tune the excited-state properties of Re(I) complexes, yielding efficient luminophores for potential applications in photochemical and photobiological contexts.

## Figures and Tables

**Table 2:** Singlet dioxygen photogeneration efficiencies (*Φ_Δ_*) and derived photophysical parameters.

	*Φ_Δ_*_/_% ^a^	$P_{O_{2}}^{T}$ ***/*% ^a^**	$f_{O_{2}}^{T}$ **/% ^a^**	*k*_q_/10^9^ M^−1^s^−1 a^
**Re(L_nHo_)Py**	45 ± 2	99.50 ± 0.01	45 ± 2	1.4 ± 0.1
**Re(L_nHo_)PTA**	29 ± 1	98.18 ± 0.01	30 ± 1	2.6 ± 0.3
**Re(L_Me-nHo_)Py**	41 ± 2	99.891 ± 0.002	41 ± 2	1.7 ± 0.2
**Re(L_Me-nHo_)PTA**	39 ± 2	99.70 ± 0.01	39 ± 2	2.7 ± 0.3

^a^ The uncertainties were calculated by the total differentials in all cases (see the Supplementary Materials for further details).

## Data Availability

Detailed information and data regarding structural characterization is available along with the corresponding NMR, IR and ESI-mass spectra (see Supplementary Materials). Detailed data regarding time-resolved as and steady-state spectroscopy as well as singlet dioxygen photogeneration is available in the Supplementary Materials or from the authors upon reasonable request.

## Supplementary Materials

The following supporting information can be downloaded at: https://www.mdpi.com/article/10.3390/ijms262110349/s1. Reference [35] is cited in the Supplementary Materials Section S5.
