# Supplementary material for: Rhenium(I) Complexes with 2-(1,2,4-Triazol-5-yl)-β-Carboline-Based Bidentate Luminophores and Neutral Co-Ligands: Towards Tunable Phosphorescence and Efficient Singlet Dioxygen Photoproduction
Source: Int J Mol Sci. 2025 Oct 24;26(21):10349. doi: 10.3390/ijms262110349 (PMC12609697; doi:10.3390/ijms262110349)
Supplement: Supplementary file 1 [file ijms-26-10349-s001.zip › ijms-3875389-supplementary.pdf]

Supporting Information

Rhenium(I) Complexes with 2-(1,2,4-Triazol-5-yl)-*β*-Carboline-Based Bidentate Luminophores and Neutral Co-Ligands: Towards Tunable Phosphorescence and Efficient Singlet Dioxygen Photoproduction

Joschua Lüke,[a,b] Iván Maisuls,[a,b] Alexander Hepp,[a] and Cristian A. Strassert[a,b]

[a] Institut für Anorganische und Analytische Chemie, Universität Münster, Corrensstraße 28/30, 48149 Münster, Germany

[b] CiMIC, SoN, CeNTech, Universität Münster, Heisenbergstraße 11, 48149 Münster, Germany

Table of contents

Section S1: Methods and materials 2

Section S2: Synthesis and characterization 3

Section S3: NMR, IR and mass spectra 12

Section S4: Photophysical characterization of the complexes in solution 45

Section S5: Reference 59

Section S1: Methods and materials

For all reactions, chemicals were used as purchased without further purification. For column chromatography silica gel 60 (0.063 – 0.200 mm) was used, purchased from Merck (Darmstadt, Germany).

S1.1 NMR spectroscopy and mass spectrometry

NMR spectra were recorded at the Institut für Anorganische und Analytische Chemie (Universität Münster) using a Bruker Avance III 400 or a Bruker Avance Neo 500 (Massachusetts, USA). All NMR measurements were performed at 300 K. The chemical shifts (*δ*) of the spectra are given in parts per million (ppm) and the signals are referenced to the residual proton signal in deuterated methylene chloride DCM-d_2 from Merck (^{1}H = 5.32 ppm / ^{13}C = 54 ppm). The signal multiplicities are given as s (singlet), d (doublet), t (triplet), q (quartet) and m (multiplet). Mass spectrometry was carried out at the Organisch-Chemisches Institut (Universität Münster) using a LTQ Orbitrap LTQXL (Thermo Fisher Scientific, Bremen, Germany) with electrospray ionization (ESI) to obtain exact mass (EM) spectra.

S1.2 Photophysical characterization

Absorption spectra were measured using quartz (Hellma®) square cuvettes on a Shimadzu UV-3600i Plus UV-vis-NIR spectrophotometer (Kyoto, Japan) and baseline-corrected. Photoluminescence quantum yields were measured with a Hamamatsu Photonics absolute PL quantum yield measurement system (C9920-02) equipped with a L9799-01 CW Xe light source (150 W), a monochromator, a C7473 photonic multi-channel analyser, an integrating sphere and employing U6039-05 software (Hamamatsu Photonics, Ltd., Shizuoka, Japan).

Steady-state excitation and emission spectra were recorded on a FluoTime 300 spectrometer from PicoQuant (Berlin, Germany) equipped with a 300 W ozone-free Xe lamp (250-1100 nm), a 10 W Xe flash-lamp (200-1100 nm, pulse width *ca*. 1 µs) with repetition rates of 1 – 300 Hz, a double grating excitation monochromator (Czerny-Turner type, grating with 1200 lines/mm, blaze wavelength: 300 nm), diode lasers (pulse width < 20 ps) operated by a computer-controlled laser driver PDL-820 "Sepia II" (repetition rate up to 80 MHz, burst mode for slow and weak decays), two emission monochromators (Czerny-Turner, selectable between double-grating blazed at 500 nm with 2.7 nm/mm dispersion and 1200 lines/mm, or single-grating blazed at 1250 nm with 5.4 nm/mm dispersion and 600 lines/mm) with adjustable slit width between 25 µm and 7 mm, Glan-Thompson polarizers for excitation (after the Xe-lamps) and emission (after the sample). Different sample holders (Peltier-cooled mounting unit ranging from -15 to 110 °C or an adjustable front-face sample holder), along with two detectors (namely a PMA Hybrid-07 from PicoQuant with transit time spread FWHM < 50 ps, 200 – 850 nm, or a H10330C-45-C3 NIR detector with transit time spread FWHM 0.4 ns, 950-1400 nm from Hamamatsu) were used. Steady-state spectra and photoluminescence lifetimes were recorded in TCSPC mode by a PicoHarp 300 (minimum base resolution 4 ps) or in MCS mode by a TimeHarp 260 (where up to several ms can be traced). Emission and excitation spectra were corrected for source intensity (lamp and grating) by standard correction curves. For samples with lifetimes in the ns order, an instrument response function calibration (IRF) was performed using a diluted Ludox® dispersion. Lifetime analysis was performed using the commercial EasyTau 2 software (PicoQuant). The quality of the fit was assessed by minimizing

the reduced chi squared function (χ^2) and visual inspection of the weighted residuals and their autocorrelation. All solvents used were of spectrometric grade (Uvasol®, Merck).

Section S2: Synthesis and characterization

All chemicals were purchased from abcr (Karlsruhe, Germany) , BLDPharm (Shanghai, China), Sigma-Aldrich (St. Louis, USA), and TCI (Tokyo, Japan), unless otherwise specified, in the highest available purity grade.

The synthesis of 1, 2 and 3was carried out by following modified reported literature procedure up to the complete formation of the *β*-carboline core.[1]

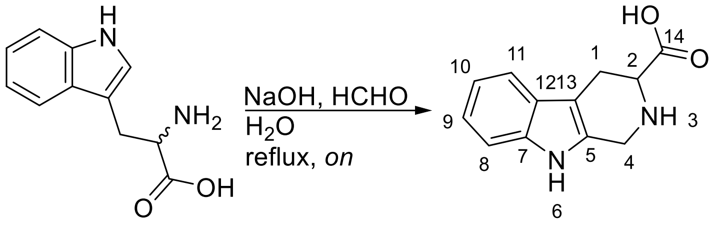

1

***1,2,3,4-Tetrahydro-β-carboline-2-carboxylic acid* (1).** (±)-Tryptophan (5.0 g, 24.48 mmol, 1.0 eq.) and sodium hydroxide (979 mg, 24.48 mmol, 1.0 eq.) were dissolved in water (50 mL) and treated with a 41% formaldehyde solution (1.97 mL, 24.48 mmol, 1.0 eq.). The mixture was refluxed for 3 hours and subsequently stirred at room temperature overnight. The reaction mixture was then adjusted to a pH 5 with acetic acid. The formed precipitate was filtered and washed with water (2 x 20 mL). Drying in vacuum was yielding **1** (4.13 g, 19.10 mmol, 78%) as a white solid.

EM-MS-ESI (MeOH, $C_{12}H_{12}N_2O_2$, *m/z*): calcd. [**1**+H]$^+$ = 217.0972, found [M+H]$^+$ = 217.1045.

^{1}H-NMR (500 MHz, DMSO-*d*$_6$): δ (ppm) = 11.08 (m, 1H), 7.37 (m, 2H), 7.02 (m, 2H), 4.25 (s, 1H), 3.66 (s, 1H), 3.14 (s, 1H), 2.85 (s, 1H), 1.90 (s, 1H).

^{13}C{^{1}H}-NMR (126 MHz, DMSO-*d*$_6$): δ (ppm) = 172.1, 170.1, 136.3, 127.8, 126.3, 121.2, 118.7, 117.8, 111.2, 106.5, 56.6, 22.9, 21.3.

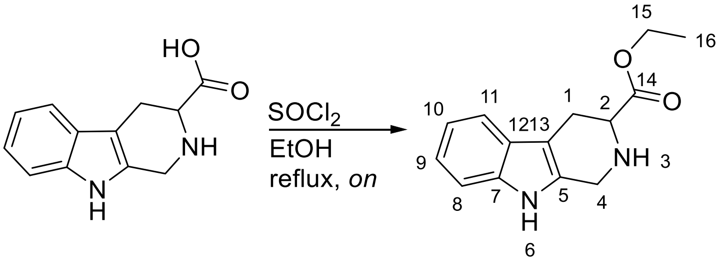

1 **2**

***Ethyl-1,2,3,4-tetrahydro-β-carboline-2-carboxylate* (2). 1** (4.0 g, 18.49 mmol, 1.0 eq.) was dissolved in ethanol (60 mL) and was cooled down in an ice bath to 0 °C. Thionylchloride was added carefully to the solution within 30 minutes the solution, which was subsequently refluxed overnight. The reaction was allowed to cool down to room temperature and the solvent was removed under reduced pressure.

The residue was taken up in water and adjusted to pH 9 with a saturated NaOH solution. The aqueous solution was extracted with ethyl acetate (3 x 30 mL) and the combined organic phases were dried over anhydrous sodium sulfate. The solvent was removed under reduced pressure, and the crude product was recrystallized from ethyl acetate with *n*-hexane. Drying with an airflow yielded **2** (3.53 g, 14.45 mmol, 78%) as a brownish solid.

EM-MS-ESI (MeOH, $C_{14}H_{16}N_2O_2$, *m/z*): calcd. [**2**+H]$^+$ = 263.0789, found [M+H]$^+$ = 263.0791.

^{1}H-NMR (400 MHz, DCM-*d*$_2$): δ (ppm) = 8.32 (s, 1H), 7.45 (dd, *J* = 8.3, 1.2 Hz, 1H), 7.28 (dt, *J* = 7.9, 1.0 Hz, 1H), 7.11 (m, 2H), 4.23 (q, *J* = 7.7 Hz, 2H), 4.08 (q, *J* = 15.8 Hz, 2H), 3.76 (dd, *J* = 9.8, 4.8 Hz, 1H), 3.11 (m, 2H), 2.86 (ddt, *J* = 15.5, 9.9, 1.9 Hz, 1H), 1.30 (t, *J* = 7.1 Hz, 3H).

^{13}C{^{1}H}-NMR (101 MHz, DCM-*d*$_2$): δ (ppm) = 173.0, 136.2, 131.6, 127.2, 121.8, 119.6, 117.9, 111.1, 107.2, 61.4, 56.0, 42.1, 25.2, 14.3.

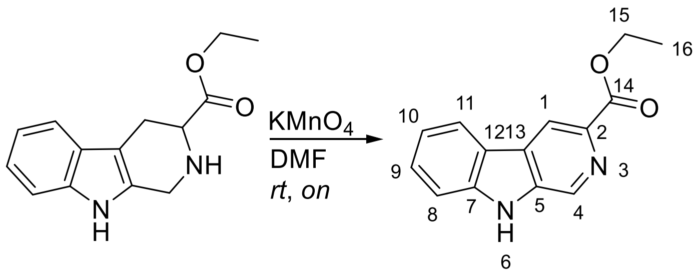

2 **3**

***Ethyl-β-carboline-2-carboxylate* (3).** **2** (3.5 g, 14.33 mmol, 1.0 eq.) was dissolved in DMF and cooled down in an ice bath to 0 °C. $KMnO_4$ (9.06 g, 57.31 mmol, 4.0 eq.) was added gradually in small portions to the solution and finally the solution was stirred overnight at room temperature. The resulting suspension was filtered off in a glass frit, the residue was washed with DMF and the solvent was removed under reduced pressure. The product was purified by flash column chromatography (SiO_2, DCM/methanol = 95:5) yielding **3** (1.58 g, 6.58 mmol, 46%) as a pale brown solid.

EM-MS-ESI (MeOH, $C_{14}H_{16}N_2O_2$, *m/z*): calcd. [**3**+H]$^+$ = 245.1285, found [M+H]$^+$ = 245.1283.

^{1}H-NMR (400 MHz, $CDCl_3$): δ (ppm) = 11.20 (s, 1H, H_6), 9.19 (d, $^4J_{HH}$ = 1.0 Hz, 1H, H_4), 8.90 (s, H_1), 8.20 (d, $^3J_{HH}$ = 7.9 Hz, 1H, H_{11}), 7.78 (dt, $^3J_{HH}$ = 8.3 Hz, $^4J_{HH}$ = 0.9 Hz, 1H, H_8), 7.33 (ddd, $^3J_{HH}$ = 8.3 Hz, $^3J_{HH}$ = 7.1 Hz, $^4J_{HH}$ = 1.2 Hz, 1H, H_9), 7.33 (ddd, $^3J_{HH}$ = 8.0 Hz, $^3J_{HH}$ = 7.1 Hz, $^4J_{HH}$ = 0.9 Hz, 1H, H_{10}), 4.56 (q, $^3J_{HH}$ = 7.1 Hz, 2H, H_{15}), 1.43 (t, $^3J_{HH}$ = 7.1 Hz, 3H, H_{16}).

^{13}C{^{1}H}-NMR (101 MHz, $CDCl_3$): δ (ppm) = 166.6 (C_{14}), 141.7 (C_7), 138.2 (C_5), 137.1 (C_2), 133.8 (C_4), 129.0 (C_9), 128.9 (C_{13}), 121.8 (C_{11}), 121.6 (C_{12}), 120.7 (C_{10}), 118.1 (C_1), 113.0 (C_8), 61.7 (C_{15}), 14.6 (C_{16}).

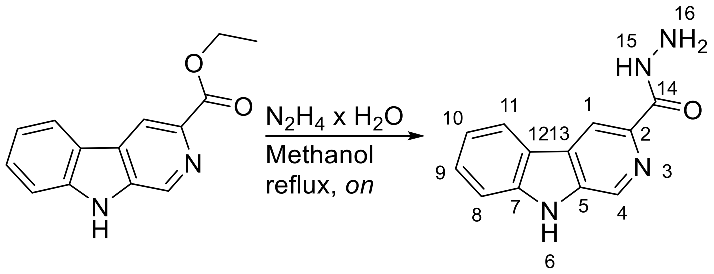

3 **4**

***β*-carboline-2-carbohydrazide (4).** **3** (1.50 g, 6.24 mmol, 1.0 eq.) was suspended in methanol (50 mL) and heated to 40 °C, followed by the addition of hydrazine monohydrate (937 µL, 18.72 mmol, 3.0 eq.) and stirred overnight. The reaction was allowed to cool down to room temperature and the solvent was removed to a minimum under reduced pressure. The resulting precipitate was filtered through a glass frit and subsequently washed with distilled water (2 x 15 mL). Drying with an airflow yielded **4** (1.19 g, 5.26 mmol, 84%) as a white solid.

EM-MS-ESI (MeOH, $C_{12}H_{10}N_4O$, *m/z*): calcd. [**4**+H]$^+$ = 227.0927, found [M+H]$^+$ = 227.0927.

^{1}H-NMR (400 MHz, $CDCl_3$): δ (ppm) = 11.29 (s, 1H, H_6), 9.67 (s, 1H, H_{15}), 8.87 (d, $^4J_{HH}$ = 1.0 Hz, 1H, H_4), 8.80 (s, H_1), 8.39 (m, 1H, H_{11}), 7.65 (m, 1H, H_8), 7.58 (m, 1H, H_9), 7.29 (ddd, $^3J_{HH}$ = 8.0 Hz, $^3J_{HH}$ = 6.9 Hz, $^4J_{HH}$ = 1.1 Hz, 1H, H_{10}).

^{13}C{^{1}H}-NMR (101 MHz, $CDCl_3$): δ (ppm) = 164.1 (C_{14}), 141.0 (C_7), 139.3 (C_2), 137.1 (C_5), 132.6 (C_4), 128.7 (C_9), 128.1 (C_{13}), 122.3 (C_{11}), 121.0 (C_{12}), 120.0 (C_{10}), 113.9 (C_1), 112.3 (C_8).

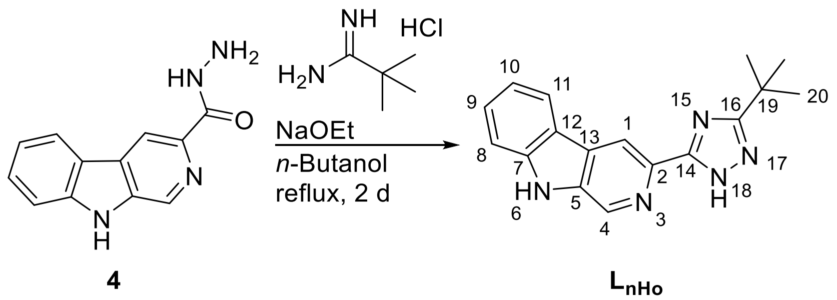

4 **L_{nHo}**

***2-(3-(tert-Butyl)-1H-1,2,4-triazol-5-yl)-β-carboline* (L_{nHo}).** Pivalamidine x HCl (196 mg, 1.44 mmol, 1.3 eq.) and NaOEt (98 mg, 1.44 mmol, 1.3 eq.) were suspended in ethanol (5 mL) and stirred at room temperature for 2 hours. Afterwards the suspension was filtered and the solvent of the filtrate was removed under reduced pressure. The resulting solid was combined with **4** (250 mg, 1.11 mmol, 1.0 eq.), the reactants were dissolved in *n*-butanol (40 mL) and were refluxed at 130 °C for 2 days. The solvent was removed under reduced pressure, and the crude product was purified by flash column chromatography (SiO_2, cyclohexane/acetone = 65:35) yielding **L_{nHo}** (120 mg, 0.37 mmol, 63%) as a white solid.

EM-MS-ESI (MeOH, $C_{17}H_{17}N_5$, *m/z*): calcd. [**L_{nHo}**+H]$^+$ = 292.1557, found [M+H]$^+$ = 292.1555.

^{1}H-NMR (400 MHz, DMSO-d_6): δ (ppm) = 13.98 (s, 1H, H_{18}), 11.83 (s, 1H, H_6), 8.95 (d, $^4J_{HH}$ = 1.1 Hz, 1H, H_4), 8.78 (s, H_1), 8.42 (d, $^3J_{HH}$ = 6.6 Hz, 1H, H_{11}), 7.64 (d, $^3J_{HH}$ = 8.2 Hz, 1H, H_8), 7.58 (ddd, $^3J_{HH}$ =

8.2 Hz, ${}^3J_{HH}$ = 6.9 Hz, ${}^4J_{HH}$ = 1.2 Hz, 1H, H_9), 7.28 (dd, ${}^3J_{HH}$ = 6.9 Hz, ${}^4J_{HH}$ = 1.2 Hz, 1H, H_{10}), 1.43 (s, H_{20}).

^{13}C{^{1}H}-NMR (101 MHz, DCM-*d₂*): δ (ppm) = 173.1 (C_{16}), 154.9 (C_{14}), 141.1 (C_7), 136.2 (C_5), 133.5 (C_4), 128.6 (C_9), 128.2 (C_{13}), 122.3 (C_{11}), 120.9 (C_{12}), 119.8 (C_{10}), 112.5 (C_1), 112.1 (C_8), 32.4 (C_{19}), 29.7 (C_{20}).

^{15}N-NMR (41 MHz, DCM-d_2): δ (ppm) = 297 (N_3).

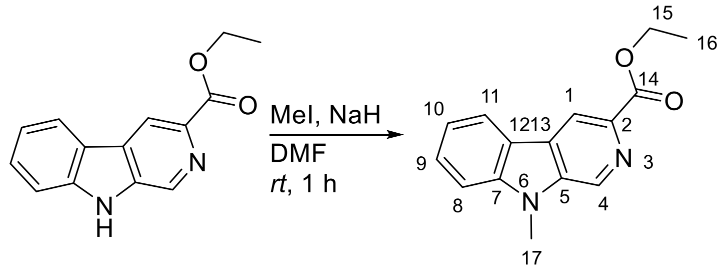

***Ethyl-6-methyl-β-carboline-2-carboxylate* (5). 3** (500 mg, 2.08 mmol, 1.0 eq.) was dissolved in dry DMF and methylene iodide (259 µL, 4.16 mmol, 2.0 eq.) and sodium hydride (60 mg, 2.50 mmol, 1.2 eq.) were added. The solution was stirred for 60 minutes at room temperature and quenched with water. The aqueous phase was extracted with ethyl acetate (3 x 50 mL) and the solvent was removed under reduced pressure. The crude product was purified by flash column chromatography (SiO_2, cyclohexane/acetone = 1:1) yielding **5** (413 mg, 1.53 mmol, 78%) as a white solid.

EM-MS-ESI (MeOH, $C_{15}H_{14}N_2O_2$, *m/z*): calcd. [**5**+H]$^+$ = 255.1128, found [M+H]$^+$ = 255.1126.

^{1}H-NMR (500 MHz, $CDCl_3$): δ (ppm) = 8.91 (d, ${}^4J_{HH}$ = 1.0 Hz, 1H, H_4), 8.84 (d, ${}^4J_{HH}$ = 1.0 Hz, 1H, H_1), 8.17 (dt, ${}^3J_{HH}$ = 7.9 Hz, ${}^4J_{HH}$ = 1.0 Hz, 1H, H_{11}), 7.64 (ddd, ${}^3J_{HH}$ = 8.3 Hz, ${}^3J_{HH}$ = 7.1 Hz, ${}^4J_{HH}$ = 1.2 Hz, 1H, H_9), 7.48 (d, ${}^3J_{HH}$ = 8.3 Hz, 1H, H_8), 7.35 (t, ${}^3J_{HH}$ = 7.5 Hz, 1H, H_{10}), 4.53 (q, ${}^3J_{HH}$ = 7.1 Hz, 2H, H_{15}), 3.95 (s, 3H, H_{17}), 1.49 (t, ${}^3J_{HH}$ = 7.1 Hz, 3H, H_{16}).

^{13}C{^{1}H}-NMR (126 MHz, $CDCl_3$): δ (ppm) = 166.3 (C_{14}), 142.2 (C_7), 138.3 (C_5), 137.9 (C_2), 131.6 (C_4), 129.0 (C_9), 128.4 (C_{13}), 122.3 (C_{11}), 121.4 (C_{12}), 120.8 (C_{10}), 117.7 (C_1), 109.8 (C_8), 61.7 (C_{15}), 29.8 (C_{17}), 14.7 (C_{16}).

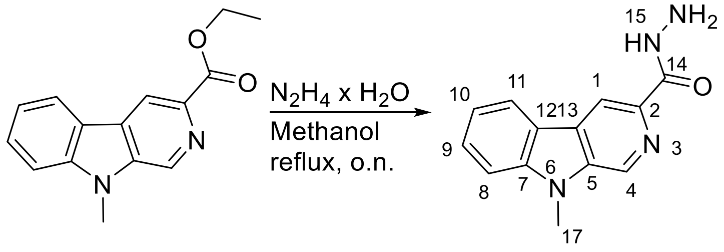

***6-Methyl-β-carboline-2-carbohydrazide* (6). 5** (390 mg, 1.53 mmol, 1.0 eq.) was suspended in ethanol (30 mL) and heated to 70 °C, followed by the addition of hydrazine monohydrate (384 µL, 7.67 mmol,

5.0 eq.) and refluxed for 2 days. The reaction was allowed to cool down to room temperature and the solvent was removed to a minimum under reduced pressure. The resulting precipitate was filtered through a glass frit and subsequently washed with distilled water (2 x 15 mL). Drying with an airflow yielded **4** (284 mg, 1.18 mmol, 77%) as a white solid.

EM-MS-ESI (MeOH $C_{13}H_{12}N_4O$, *m/z*): calcd. [**6**+H]$^+$ = 241.1084, found [M+H]$^+$ = 241.1080.

^{1}H-NMR (400 MHz, DMSO-*d*$_6$): δ (ppm) = 9.70 (d, $^3J_{HH}$ = 5.2 Hz, 1H, H_{15}), 9.02 (d, $^4J_{HH}$ = 1.0 Hz, 1H, H_4), 8.82 (d, $^4J_{HH}$ = 1.0 Hz, 1H, H_1), 8.42 (dt, $^3J_{HH}$ = 7.9 Hz, $^4J_{HH}$ = 1.0 Hz, 1H, H_{11}), 7.74 (dt, $^3J_{HH}$ = 8.4 Hz, $^4J_{HH}$ = 1.0 Hz, 1H, H_8), 7.66 (ddd, $^3J_{HH}$ = 8.3 Hz, $^3J_{HH}$ = 7.1 Hz, $^4J_{HH}$ = 1.2 Hz, 1H, H_9), 7.34 (ddd, $^3J_{HH}$ = 7.9 Hz, $^3J_{HH}$ = 7.0 Hz, $^4J_{HH}$ = 1.0 Hz, 1H, H_{10}), 4.54 (d, $^3J_{HH}$ = 4.3 Hz, 2H, H_{16}), 4.04 (s, 3H, H_{17}) .

^{13}C{^{1}H}-NMR (101 MHz, DMSO-*d*$_6$): δ (ppm) = 163.9 (C_{14}), 141.8 (C_7), 139.6 (C_2), 137.6 (C_5), 131.2 (C_4), 128.7 (C_9), 127.6 (C_{13}), 122.3 (C_{11}), 120.6 (C_{12}), 120.1 (C_{10}), 113.6 (C_1), 110.3 (C_8), 29.5 (C_{17}).

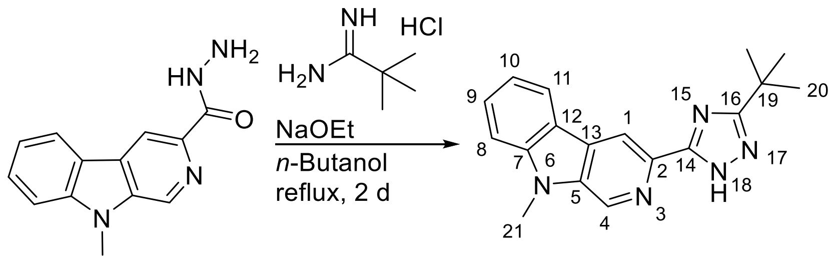

6 **$L_{Me\text{-}nHo}$**

2-(3-(tert-Butyl)-1H-1,2,4-triazol-5-yl)-6-methyl-β-carboline **($L_{Me\text{-}nHo}$).** Pivalamidine x HCl (628 mg, 4.60 mmol, 1.3 eq.) and NaOEt (313 mg, 4.60 mmol, 1.3 eq.) were suspended in ethanol (5 mL) and stirred at room temperature for 2 hours. Afterwards, the suspension was filtered and the solvent of the filtrate was removed under reduced pressure. The resulting solid was combined with **6** (850 mg, 3.54 mmol, 1.0 eq.), the reactants were dissolved in *n*-butanol (60 mL) and then refluxed at 130 °C for 2 days. The solvent was removed under reduced pressure, and the crude product was purified by flash column chromatography (SiO_2, DCM/methanol = 98:2) yielding **$L_{Me\text{-}nHo}$** (531 mg, 1.74 mmol, 49%) as a white solid.

EM-MS-ESI (MeOH, $C_{18}H_{19}N_5$, *m/z*): calcd. [**$L_{Me\text{-}nHo}$**+H]$^+$ = 306.1713, found [M+H]$^+$ = 306.1709.

^{1}H-NMR (400 MHz, DCM-*d*$_2$): δ (ppm) = 8.92 (d, $^4J_{HH}$ = 1.0 Hz, 1H, H_4), 8.88 (d, $^4J_{HH}$ = 1.0 Hz, H_1), 8.25 (dt, $^3J_{HH}$ = 7.9 Hz, $^4J_{HH}$ = 1.0 Hz, 1H, H_{11}), 7.66 (ddd, $^3J_{HH}$ = 8.3 Hz, $^3J_{HH}$ = 7.1 Hz, $^4J_{HH}$ = 1.2 Hz, 1H, H_9), 7.52 (dt, $^3J_{HH}$ = 8.3 Hz, $^4J_{HH}$ = 0.9 Hz, 1H, H_8), 7.34 (ddd, $^3J_{HH}$ = 8.0 Hz, $^3J_{HH}$ = 7.1 Hz, $^4J_{HH}$ = 1.0 Hz, 1H, H_{10}), 3.95 (s, 3H, H_{21}), 1.48 (s, 9H, H_{20}).

^{13}C{^{1}H}-NMR (101 MHz, DCM-*d*$_2$): δ (ppm) = 172.4 (C_{16}), 156.6 (C_{14}), 142.9 (C_7), 137.8 (C_5), 137.3 (C_2), 132.0 (C_4), 129.6 (C_9), 129.6 (C_{13}), 122.8 (C_{11}), 121.7 (C_{12}), 120.8 (C_{10}), 113.6 (C_1), 110.2 (C_8), 33.3 (C_{19}), 30.1 (C_{21}), 29.9 (C_{20}).

^{15}N-NMR (41 MHz, DCM-*d*$_2$): δ (ppm) = 290 (N_3), 107 (N_6).

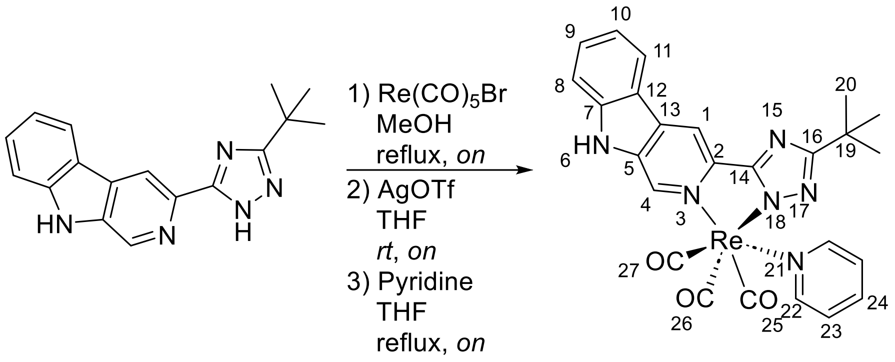

L_{nHo} **Re(L_{nHo})Py**

Re(L_{nHo})Py. Re(CO)$_5$Br (70 mg, 0.17 mmol, 1.0 eq.) and **L_{nHo}** (50 mg, 0.17 mmol, 1.0 eq.) were dissolved in methanol (10 mL). The solution was subsequently purged with argon for 10 minutes and was stirred at 70 °C for two days. The reaction mixture was cooled to room temperature, filtered through a glass frit and the solvent was removed under reduced pressure. The crude product was used without further purification and dissolved in THF (15 mL) with silver triflate (44 mg, 0.17 mmol, 1.0 eq.). The solution subsequently was purged with argon for 5 minutes and was refluxed overnight. The reaction mixture was allowed to cool down to room temperature, the resulting precipitate was filtered through a glass frit and pyridine (14 µL, 0.17 mmol, 1.0 eq.) was added to the solution. The solution was subsequently purged with argon for 10 minutes and was refluxed overnight. The reaction mixture was allowed to cool down to room temperature and purified by flash column chromatography (SiO_2, DCM/methanol = 96:4). Re-crystallization from *n*-hexane in DCM yielded **Re(L_{nHo})Py** (62 mg, 0.11 mmol, 68%) as a yellow amorphous solid.

EM-MS-ESI (MeOH, $C_{25}H_{21}N_6O_3Re$, , *m/z*): calcd. [**Re(L_{nHo})Py**+H]$^+$ = 641.1306, found [M+H]$^+$ = 641.1306.

^{1}H-NMR (500 MHz, DCM-*d*$_2$): δ (ppm) = 11.07 (s, 1H, H_6), 9.23 (s, 1H, H_4), 8.63 (s, 1H, H_1), 8.27 (d, $^3J_{HH}$ = 5.6 Hz, 1H, H_{22}), 8.04 (d, $^3J_{HH}$ = 8.0 Hz, 1H, H_{11}), 7.56 (m, 3H, $H_{8/9/24}$), 7.28 (ddd, $^3J_{HH}$ = 8.0 Hz, $^3J_{HH}$ = 6.8 Hz, $^4J_{HH}$ = 1.2 Hz, 1H, H_{10}), 7.05 (t, $^3J_{HH}$ = 6.9 Hz, 1H, H_{23}), 1.49 (s, 9H, H_{20}).

^{13}C{^{1}H}-NMR (126 MHz, DCM-*d*$_2$): δ (ppm) = 199.2 (C_{CO}), 197.2 (C_{CO}), 194.2 (C_{CO}), 175.9 (C_{16}), 165.5 (C_{14}), 152.5 (C_{22}), 143.1 (C_7), 142.1 (C_2), 139.0 (C_{24}), 137.0 (C_5), 136.8 (C_4), 132.2 (C_{13}), 130.9 (C_9), 126.2 (C_{23}), 123.0 (C_{11}), 121.6 (C_{10}), 121.5 (C_{12}), 113.2 (C_1), 112.9 (C_8), 33.7 (C_{19}), 30.7 (C_{20}).

^{15}N-NMR (51 MHz, DCM-*d*$_2$): δ (ppm) = 244 (N_{21}), 227 (N_3).

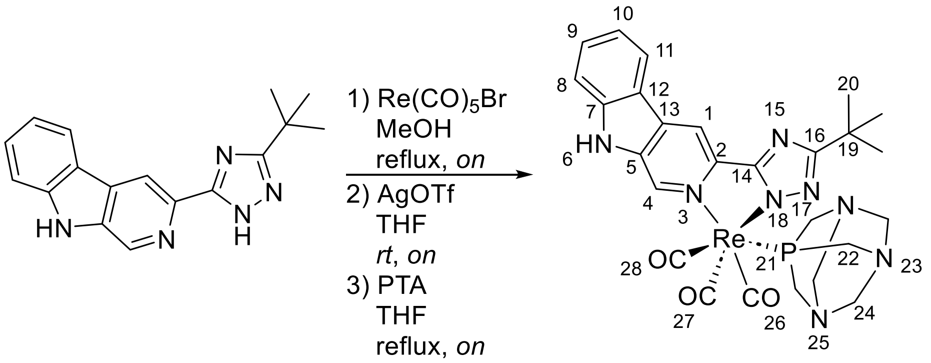

L_{nHo} **$Re(L_{nHo})PTA$**

***Re(L$_{nHo}$)PTA*.** $Re(CO)_5Br$ (75 mg, 0.18 mmol, 1.0 eq.) and **L_{nHo}** (54 mg, 0.18 mmol, 1.0 eq.) were dissolved in methanol (10 mL). The solution was subsequently purged with argon for 10 minutes and was stirred at 70 °C for two days. The reaction mixture was cooled to room temperature, filtered through a glass frit and the solvent was removed under reduced pressure. The crude product was used without further purification and then dissolved in THF (15 mL) with silver triflate (46 mg, 0.18 mmol, 1.0 eq.). The solution subsequently was purged with argon for 5 minutes and refluxed overnight. The reaction mixture was allowed to cool down to room temperature, the resulting precipitate was filtered through a glass frit and 1,3,5-triaza-7-phosphaadamantane (PTA; 25 mg, 0.18 mmol, 1.0 eq.) was added to the solution. The solution was subsequently purged with argon for 10 minutes and was refluxed overnight. The reaction mixture was allowed to cool down to room temperature and purification by flash column chromatography (SiO_2, DCM/methanol = 97:3) yielded **Re(L_{nHo})PTA** (56 mg, 0.078 mmol, 43%) as a yellow amorphous solid.

EM-MS-ESI (MeOH, $C_{26}H_{28}N_8O_3PRe$, *m/z*): calcd. [**Re(L_{nHo})PTA**+H]$^+$ = 764.2113, found [M+H]$^+$ = 764.2108.

^{1}H-NMR (400 MHz, DCM-*d*$_2$): δ (ppm) = 11.43 (s, 1H, H_6), 8.95 (s, 1H, H_4), 8.77 (s, 1H, H_1), 8.09 (d, $^3J_{HH}$ = 8.0 Hz, 1H, H_{11}), 7.55 (ddd, $^3J_{HH}$ = 8.2 Hz, $^3J_{HH}$ = 6.9 Hz, $^4J_{HH}$ = 1.2 Hz, 1H, H_9), 7.49 (d, $^3J_{HH}$ = 8.4 Hz, 1H, H_8), 7.27 (ddd, $^3J_{HH}$ = 8.0 Hz, $^3J_{HH}$ = 6.9 Hz, $^4J_{HH}$ = 1.1 Hz, 1H, H_{10}), 4.29 (d, $^2J_{HH}$ = 13.3 Hz, 3H, H_{24}), 4.07 (d, $^2J_{HH}$ = 13.1 Hz, 3H, $H_{24'}$), 3.48 (s, 6H, H_{22}), 1.43 (s, 9H, H_{20}).

^{13}C{^{1}H}-NMR (101 MHz, DCM-*d*$_2$): δ (ppm) = 195.3 (d, $^2J_{CP}$ = 7.5 Hz, C_{CO}), 193.7 (d, $^2J_{CP}$ = 7.9 Hz, C_{CO}), 188.9 (d, $^2J_{CP}$ = 67.8 Hz, C_{CO}), 173.8 (C_{16}), 163.2 (C_{14}), 143.3 (C_7), 142.9 (d, $^4J_{CP}$ = 23.8 Hz, C_2), 138.0 (C_4), 137.2 (C_5), 131.8 (C_{13}), 131.0 (C_9), 123.0 (C_{11}), 121.7 (C_{10}), 121.4 (C_{12}), 114.6 (C_1), 113.2 (C_8), 73.3 (d, $^1J_{CP}$ = 6.6 Hz, C_{22}), 50.5 (d, $^3J_{CP}$ = 14.9 Hz, C_{23}), 33.7 (C_{19}), 30.4 (C_{20}).

^{15}N-NMR (41 MHz, DCM-*d*$_2$): δ (ppm) = 208 (N_2), 33 ($N_{23/25}$).

^{31}P{^{1}H}-NMR (162 MHz, DCM-*d*$_2$): δ (ppm) = - 75.4 (P_{21}).

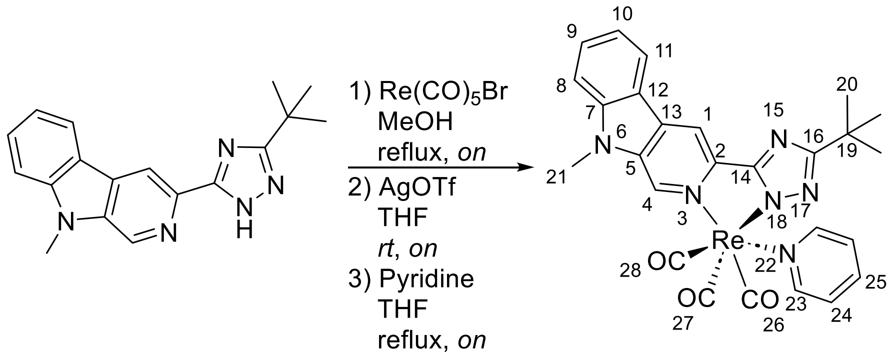

L$_{Me\text{-}nHo}$ **Re(L$_{Me\text{-}nHo}$)Py**

***Re(L$_{Me\text{-}nHo}$)Py*.** $Re(CO)_5Br$ (66 mg, 0.16 mmol, 1.0 eq.) and **L$_{Me\text{-}nHo}$** (50 mg, 0.16 mmol, 1.0 eq.) were dissolved in methanol (10 mL). The solution was subsequently purged with argon for 10 minutes and then stirred at 70 °C for two days. The reaction mixture was cooled to room temperature, filtered through a glass frit and the solvent was removed under reduced pressure. The crude product was used without further purification and then dissolved in THF (15 mL) with silver triflate (42 mg, 0.16 mmol, 1.0 eq.). The solution was subsequently purged with argon for 5 minutes and then refluxed overnight. The reaction mixture was allowed to cool down to room temperature, the resulting precipitate was filtered through a glass frit and pyridine (13 µL, 0.16 mmol, 1.0 eq.) was added to the solution. The solution was subsequently purged with argon for 10 minutes and was refluxed overnight. The reaction mixture was allowed to cool down to room temperature and purified by flash column chromatography (SiO_2, DCM/methanol = 96:4). Re-crystallization from *n*-hexane in DCM yielded **Re(L$_{Me\text{-}nHo}$)Py** (62 mg, 0.11 mmol, 66%) as a yellow amorphous solid.

EM-MS-ESI (MeOH, $C_{26}H_{23}N_6O_3Re$, *m/z*): calcd. [**Re(L$_{Me\text{-}nHo}$)Py**+H]$^+$ = 655.1463, found [M+H]$^+$ = 655.1471.

^{1}H-NMR (500 MHz, DCM-d_2): δ (ppm) = 9.14 (d, $^4J_{HH}$ = 0.9 Hz, 1H, H_4), 8.66 (s, 1H, H_1), 8.34 (dt, $^3J_{HH}$ = 5.0 Hz, $^4J_{HH}$ = 1.6 Hz, 1H, H_{23}), 8.21 (dt, $^3J_{HH}$ = 7.9 Hz, $^3J_{HH}$ = 1.0 Hz, 1H, H_{11}), 7.72 (ddd, $^3J_{HH}$ = 8.3 Hz, $^3J_{HH}$ = 7.1 Hz, $^4J_{HH}$ = 1.2 Hz, 1H, H_9), 7.65 (tt, $^3J_{HH}$ = 7.6 Hz, $^4J_{HH}$ = 1.6 Hz, 1H, H_{25}), 7.57 (dt, $^3J_{HH}$ = 8.5 Hz, $^4J_{HH}$ = 0.9 Hz, 1H, H_8), 7.36 (ddd, $^3J_{HH}$ = 8.0 Hz, $^3J_{HH}$ = 7.1 Hz, $^4J_{HH}$ = 0.9 Hz, 1H, H_{10}), 7.13 (m, 1H, H_{24}), 4.05 (s, 3H, H_{21}), 1.47 (s, 9H, H_{20}).

^{13}C{^{1}H}-NMR (126 MHz, DCM-d_2): δ (ppm) = 199.6 (C_{CO}), 197.3 (C_{CO}), 194.5 (C_{CO}), 175.8 (C_{16}), 165.1 (C_{14}), 152.7 (C_{23}), 144.1 (C_7), 142.6 (C_2), 139.0 (C_{25}), 137.7 (C_5), 134.4 (C_4), 131.8 (C_{13}), 131.0 (C_9), 126.1 (C_{24}), 123.4 (C_{11}), 121.6 (C_{10}), 121.1 (C_{12}), 113.1 (C_1), 110.4 (C_8), 33.6 (C_{19}), 30.7 (C_{20}), 30.5 (C_{21}).

^{15}N-NMR (51 MHz, DCM-d_2): δ (ppm) = 244 (N_{21}), 227 (N_3), 108 (N_6).

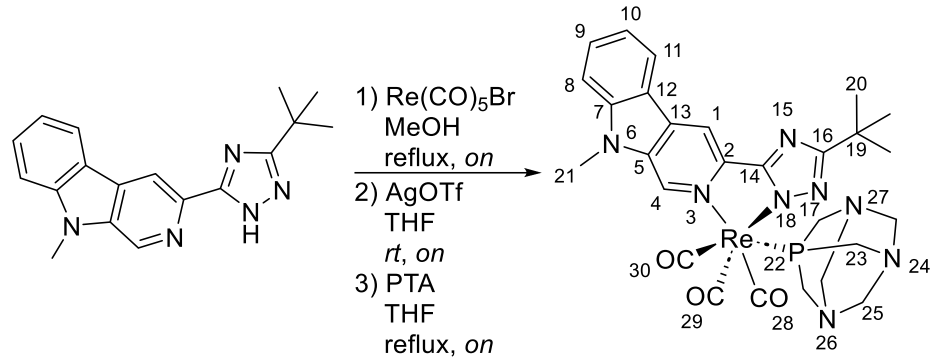

$\mathbf{L_{Me\text{-}nHo}}$ $\mathbf{Re(L_{Me\text{-}nHo})PTA}$

Re(L $_{\textbf{\textit{Me-nHo}}}$***)PTA*.** $Re(CO)_5Br$ (66 mg, 0.16 mmol, 1.0 eq.) and $\mathbf{L_{Me\text{-}nHo}}$ (50 mg, 0.16 mmol, 1.0 eq.) were dissolved in methanol (10 mL). The solution was subsequently purged with argon for 10 minutes and was stirred at 70 °C for two days. The reaction mixture was cooled to room temperature, filtered through a glass frit and the solvent was removed under reduced pressure. The crude product was used without further purification and then dissolved in THF (15 mL) with silver triflate (42 mg, 0.16 mmol, 1.0 eq.). The solution subsequently was purged with argon for 5 minutes and then refluxed overnight. The reaction mixture was allowed to cool down to room temperature, the resulting precipitate was filtered through a glass frit and PTA (26 mg, 0.16 mmol, 1.0 eq.) was added to the solution. The solution was subsequently purged with argon for 10 minutes and then refluxed overnight. The reaction mixture was allowed to cool down to room temperature and purified by flash column chromatography (SiO_2, DCM/methanol = 98:2). Re-crystallization from *n*-hexane in DCM yielded $\mathbf{Re(L_{Me\text{-}nHo})PTA}$ (47 mg, 0.064 mmol, 40%) as a pale yellow amorphous solid.

EM-MS-ESI (MeOH, $C_{27}H_{30}N_8O_3PRe$, *m/z*): calcd. [$\mathbf{Re(L_{Me\text{-}nHo})PTA}$+H]$^+$ = 733.1810, found [M+H]$^+$ = 733.1810.

^{1}H-NMR (400 MHz, DCM-d_2): δ (ppm) = 8.97 (d, $^4J_{HH}$ = 0.9 Hz, 1H, H_4), 8.83 (d, $^4J_{HH}$ = 0.9 Hz, 1H, H_1), 8.30 (dt, $^3J_{HH}$ = 7.9 Hz, $^4J_{HH}$ = 1.0 Hz, 1H, H_{11}), 7.75 (ddd, $^3J_{HH}$ = 8.3 Hz, $^3J_{HH}$ = 7.1 Hz, $^4J_{HH}$ = 1.2 Hz, 1H, H_9), 7.58 (dt, $^3J_{HH}$ = 8.4 Hz, $^4J_{HH}$ = 0.9 Hz, 1H, H_8), 7.41 (ddd, $^3J_{HH}$ = 8.0 Hz, $^3J_{HH}$ = 7.1 Hz, $^4J_{HH}$ = 0.9 Hz, 1H, H_{10}), 4.37 (d, $^2J_{HH}$ = 13.0 Hz, 3H, H_{25}), 4.16 (dt, $^2J_{HH}$ = 13.1 Hz, $^4J_{HH}$ = 1.3 Hz, 3H, $H_{25'}$), 4.00 (s, 3H, H_{21}), 3.48 (m, 6H, H_{25}), 1.48 (s, 9H, H_{20}).

^{13}C{^{1}H}-NMR (101 MHz, DCM-d_2): δ (ppm) = 196.2 (d, $^2J_{CP}$ = 7.5 Hz, C_{CO}), 194.5 (d, $^2J_{CP}$ = 8.0 Hz, C_{CO}), 189.7 (d, $^2J_{CP}$ = 59.1 Hz, C_{CO}), 176.1 (C_{16}), 164.1 (C_{14}), 144.1 (C_7), 142.7 (C_2), 137.5 (C_5), 135.0 (C_4), 131.8 (C_{13}), 131.0 (C_9), 123.4 (C_{11}), 121.6 (C_{10}), 121.2 (C_{12}), 113.2 (C_1), 110.4 (C_8), 73.4 (d, $^1J_{CP}$ = 6.5 Hz, C_{23}), 50.5 (d, $^3J_{CP}$ = 15.0 Hz, C_{25}), 33.5 (C_{19}), 30.8 (C_{21}), 30.4 (C_{20}).

^{15}N-NMR (41 MHz, DCM-d_2): δ (ppm) = 208 (N_2), 107 (N_6), 33 ($N_{24/26}$).

^{31}P{^{1}H}-NMR (162 MHz, DCM-d_2): δ (ppm) = - 75.1 (P_{22}).

Section S3: NMR, IR and mass spectra

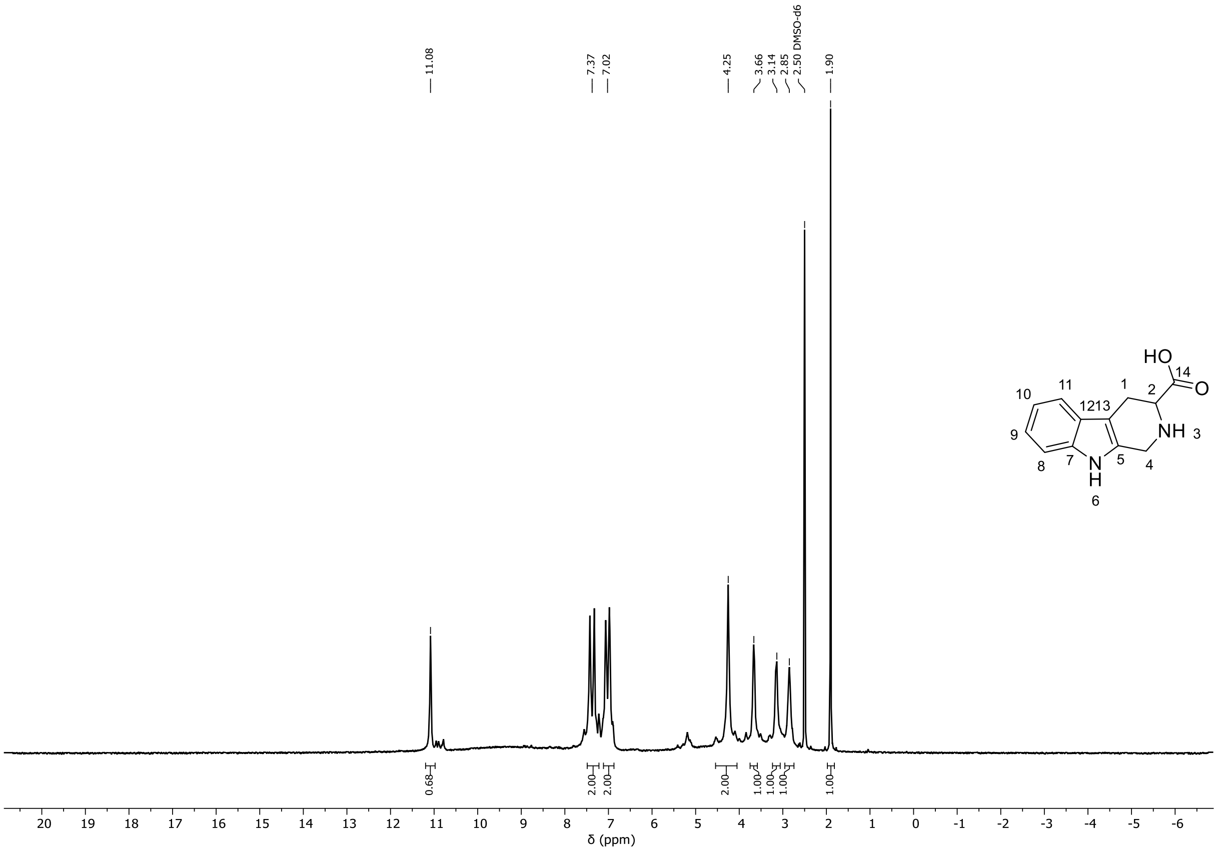

Figure S-1. ^{1}H-NMR spectrum (500 MHz, DMSO-d_6) of **1**.

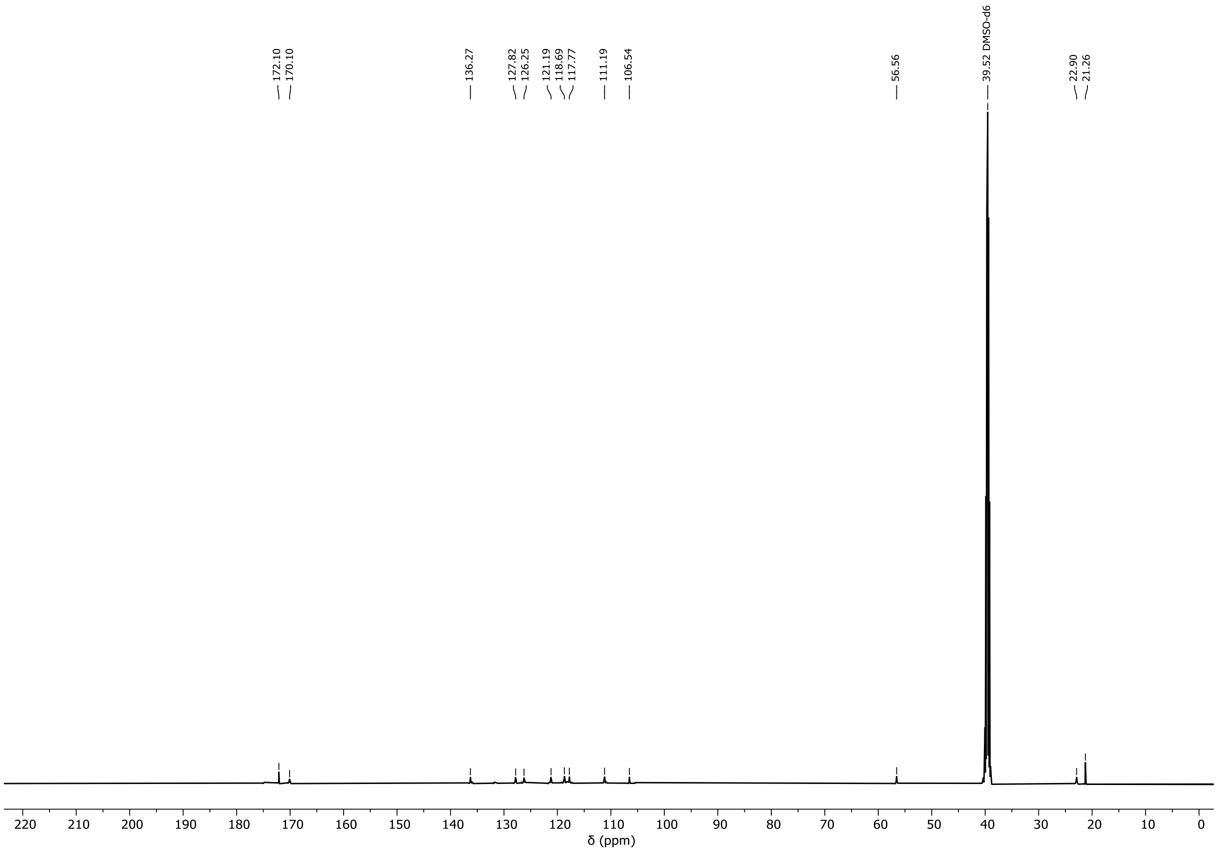

Figure S-2. ^{13}C{^{1}H}-NMR spectrum (126 MHz, DMSO-d_6) of **1**.

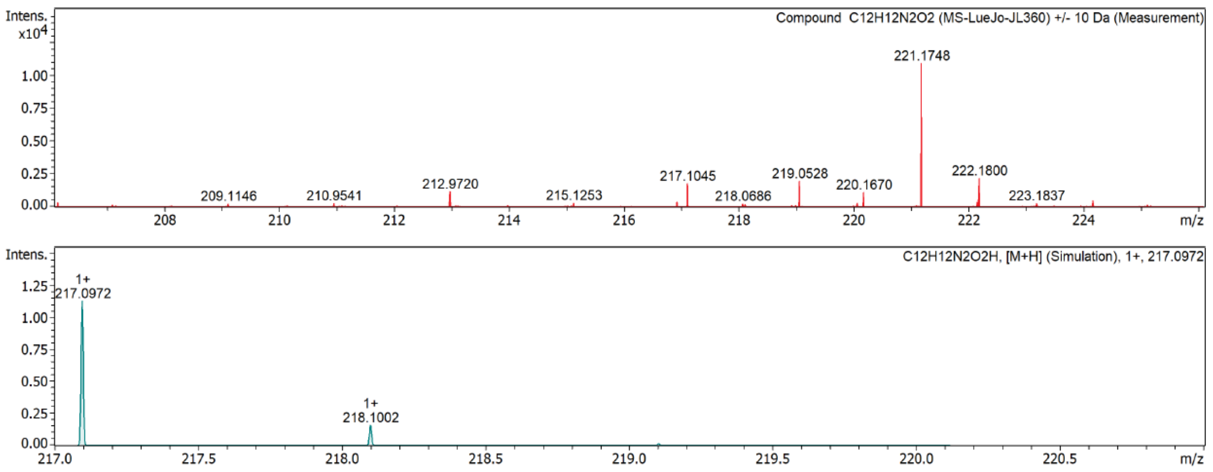

Figure S-3. Mass spectrum of **1** (MeOH). Additional simulation of the [**1**+H]$^+$ adduct.

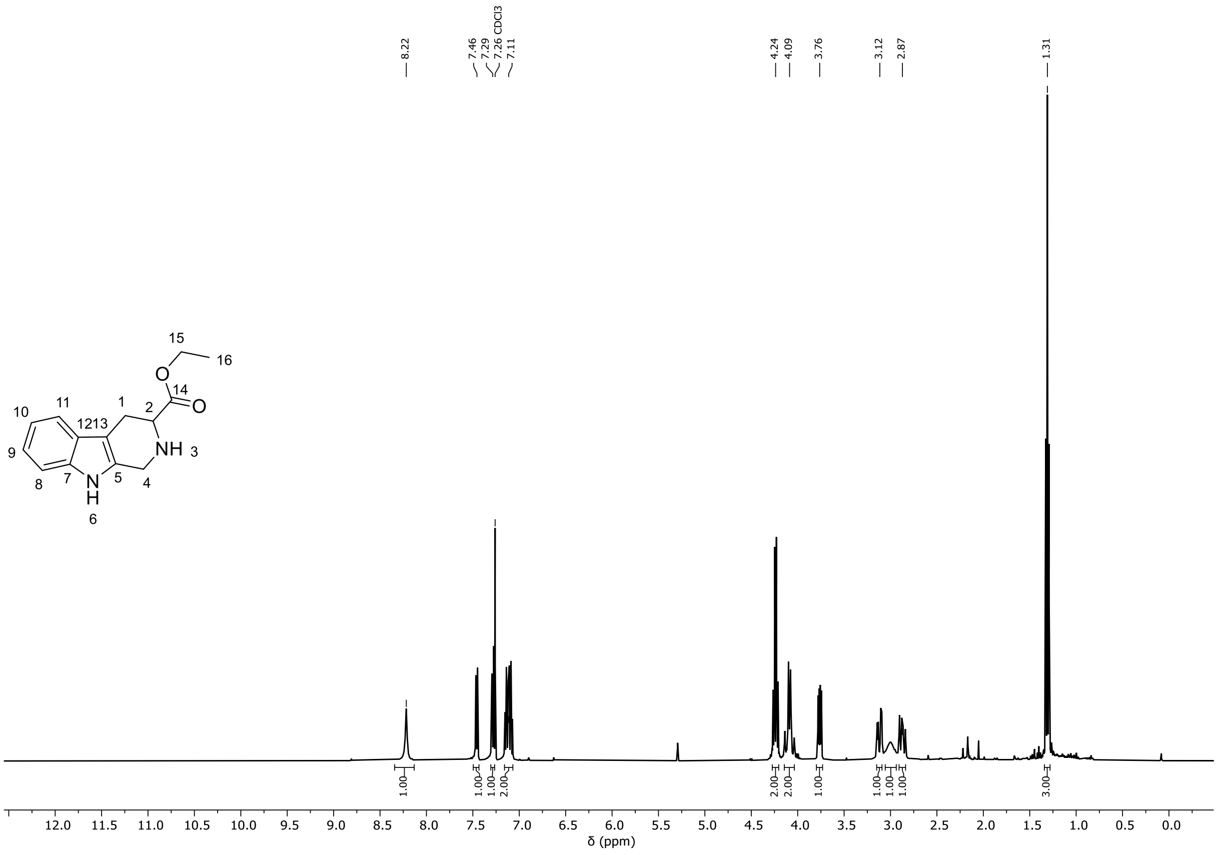

Figure S-4. ^{1}H-NMR spectrum (500 MHz, $CDCl_3$) of **2**.

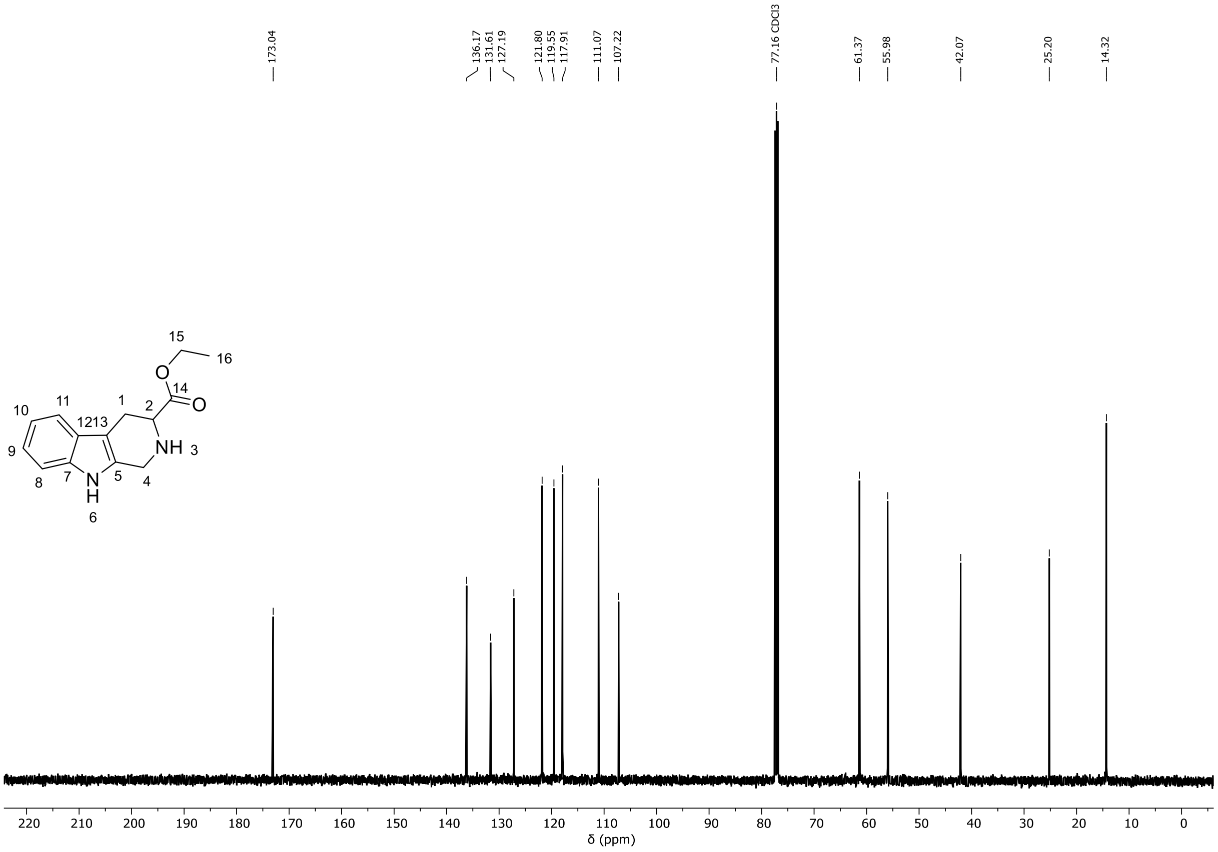

Figure S-5: $^{13}C\{^{1}H\}$-NMR spectrum (126 MHz, $CDCl_3$) of **2**.

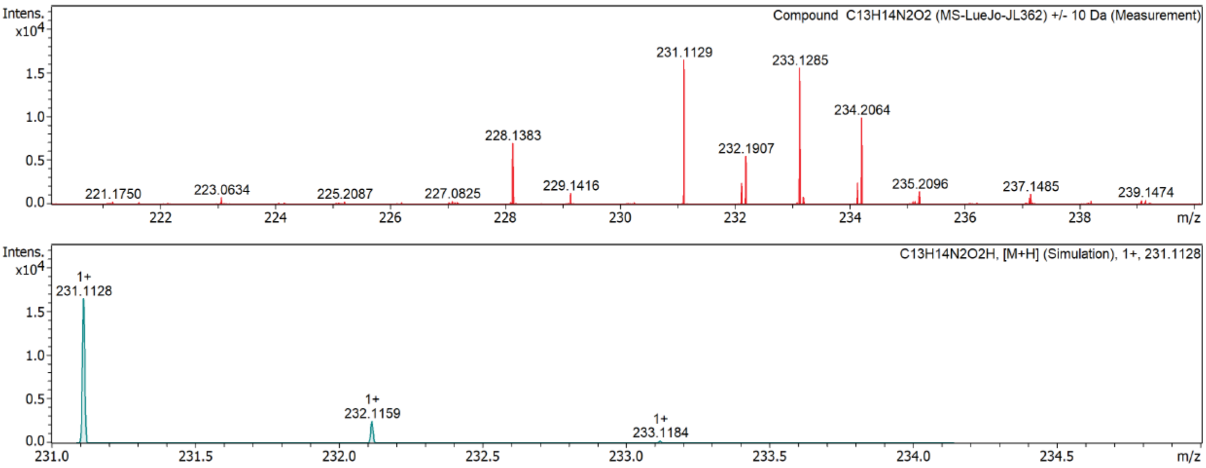

Figure S-6. Mass spectrum of **2** (MeOH). Additional simulation of the [**2**+H]$^{+}$ adduct.

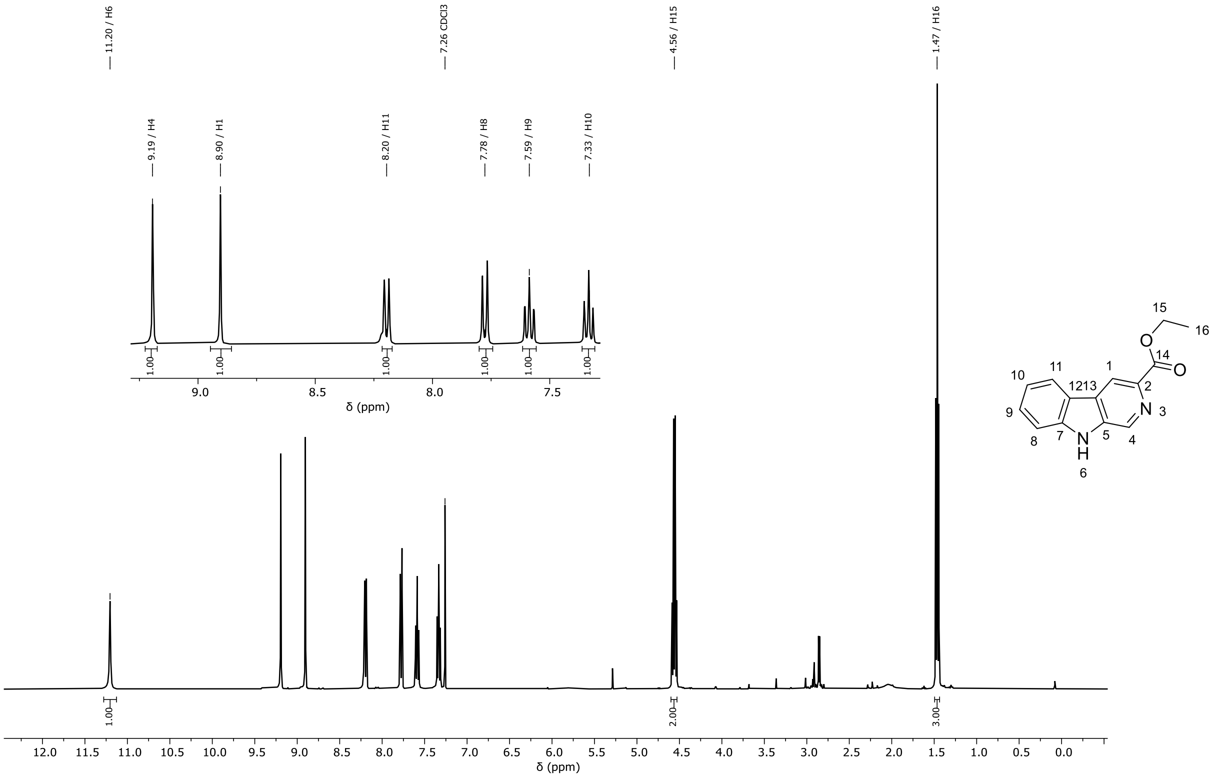

Figure S-7. ^{1}H-NMR spectrum (400 MHz, $CDCl_3$) of **3**.

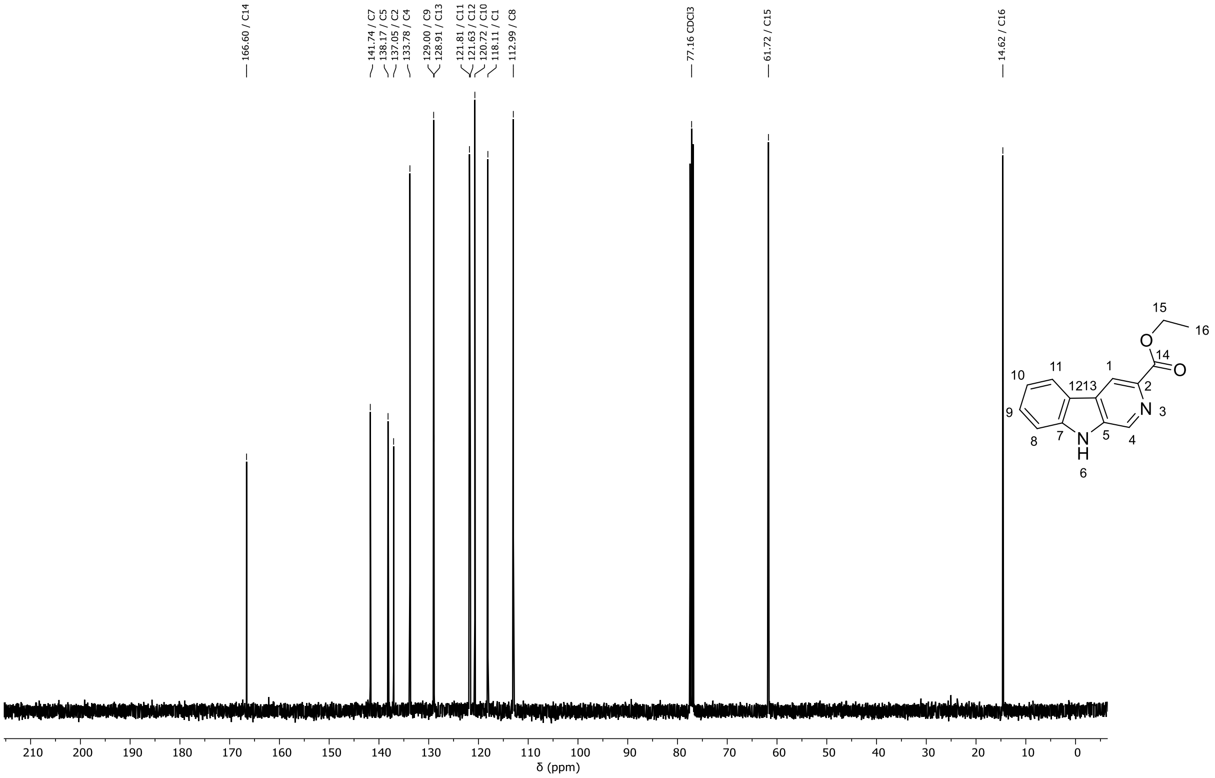

Figure S-8. $^{13}C\{^{1}H\}$-NMR spectrum (101 MHz, $CDCl_3$) of **3**.

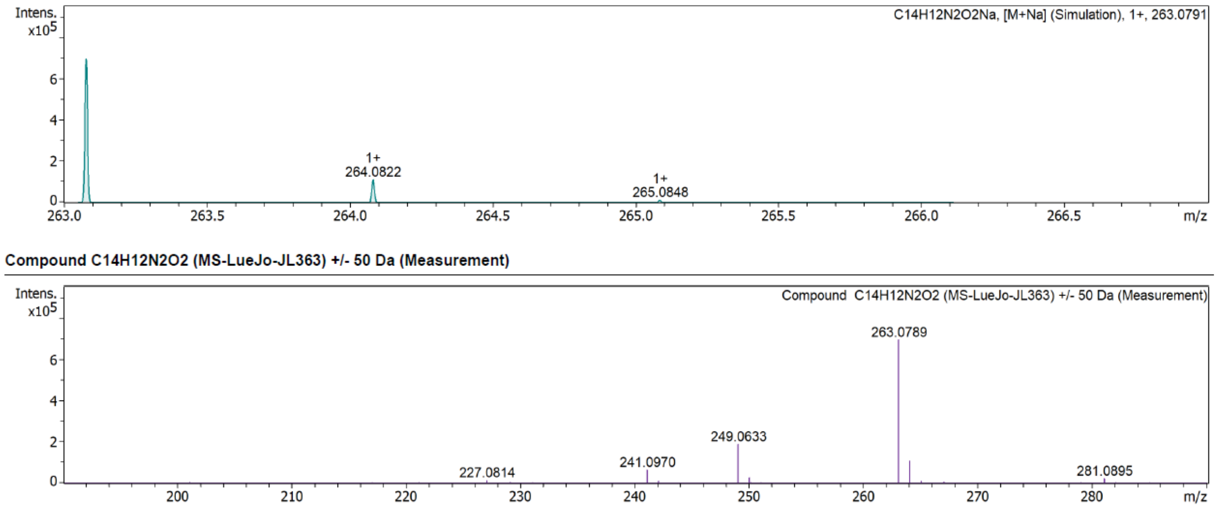

Figure S-9. Mass spectrum of **3** (MeOH). Additional simulation of the [**3**+Na]$^{+}$ adduct.

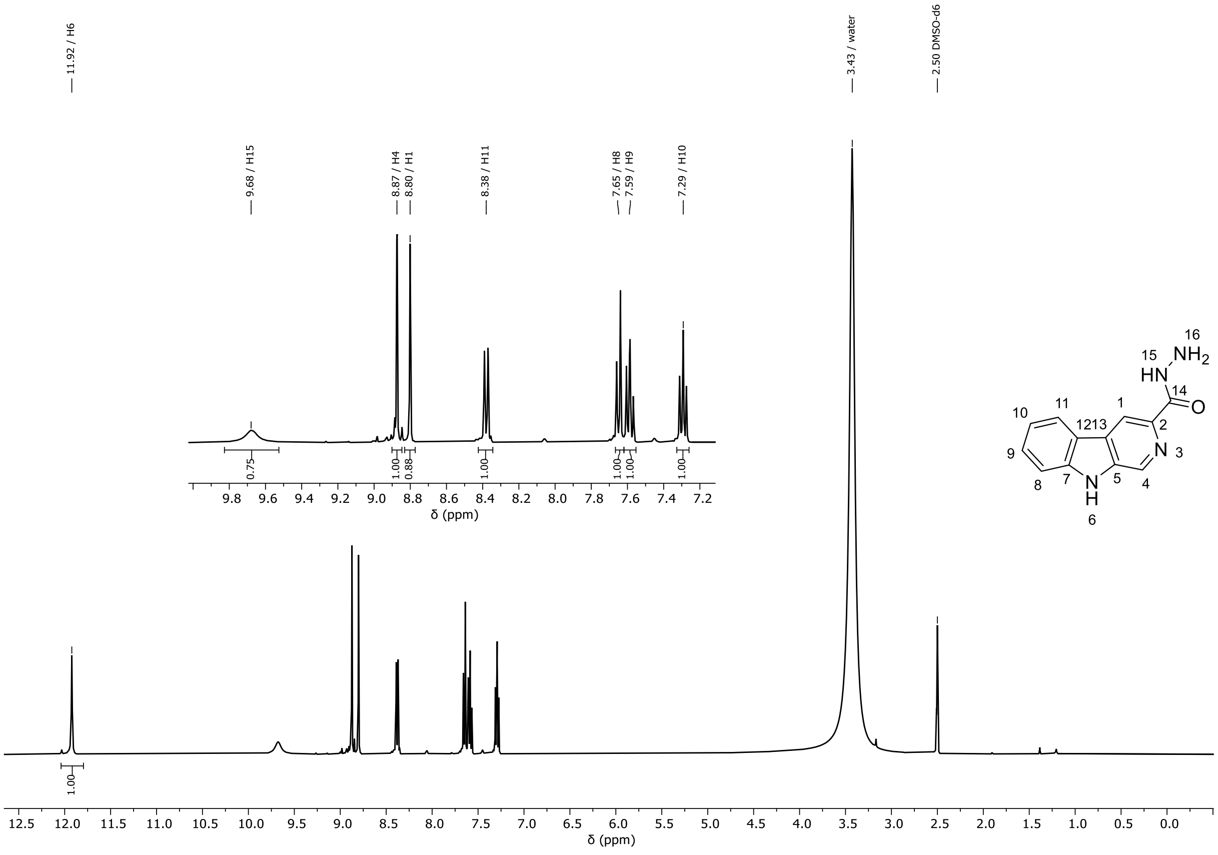

Figure S-10. ^{1}H-NMR spectrum (400 MHz, DMSO-d_6) of **4**.

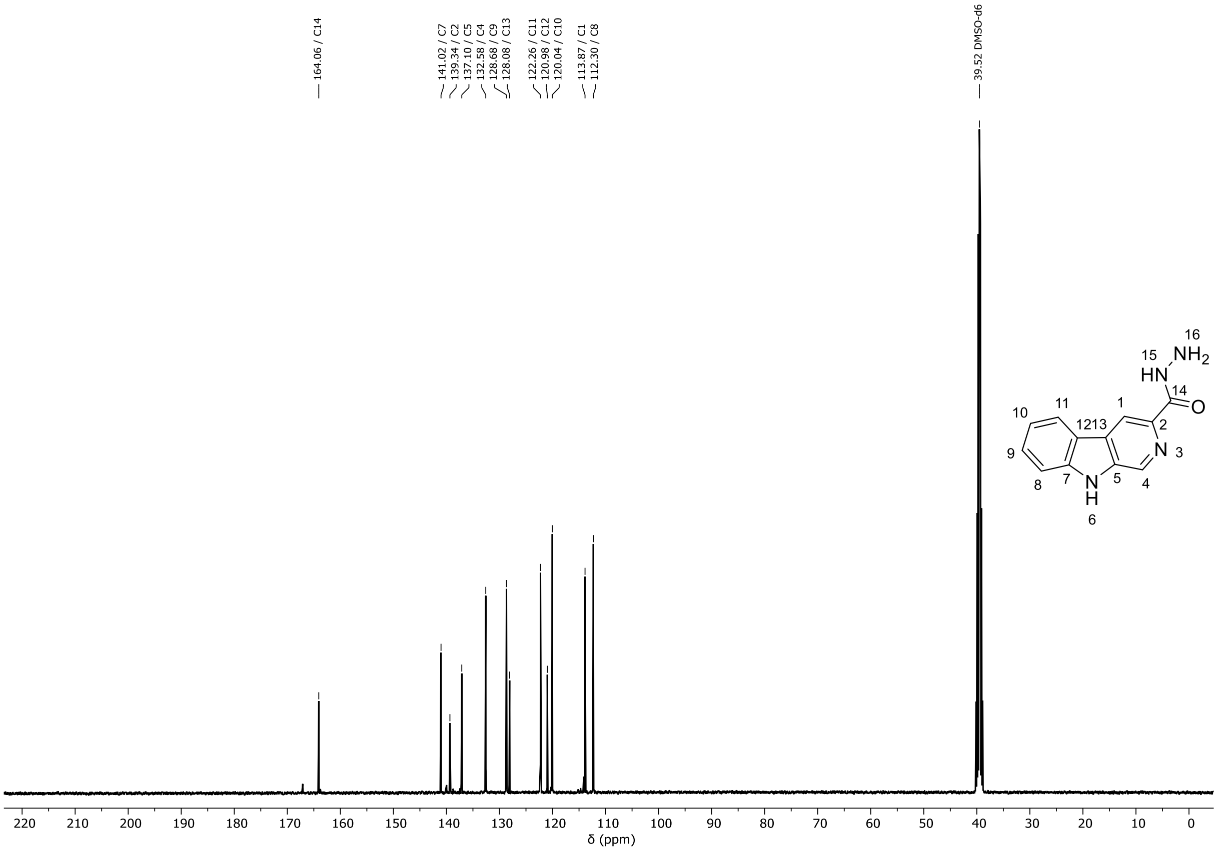

Figure S-11: $^{13}C\{^{1}H\}$-NMR spectrum (101 MHz, DMSO-d_6) of **4**.

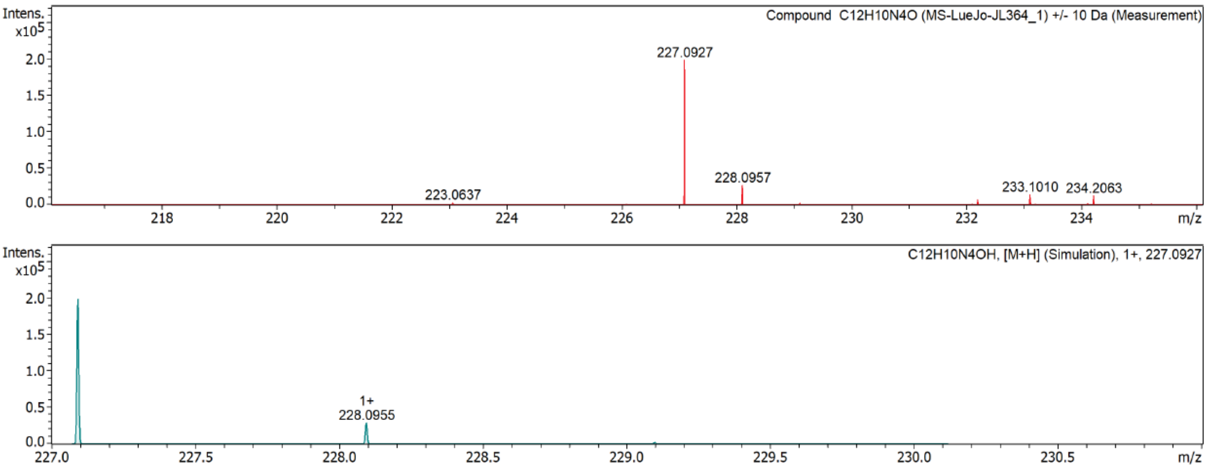

Figure S-12. Mass spectrum of **2.2** (MeOH). Additional simulation of the [**4**+H]$^+$ adduct.

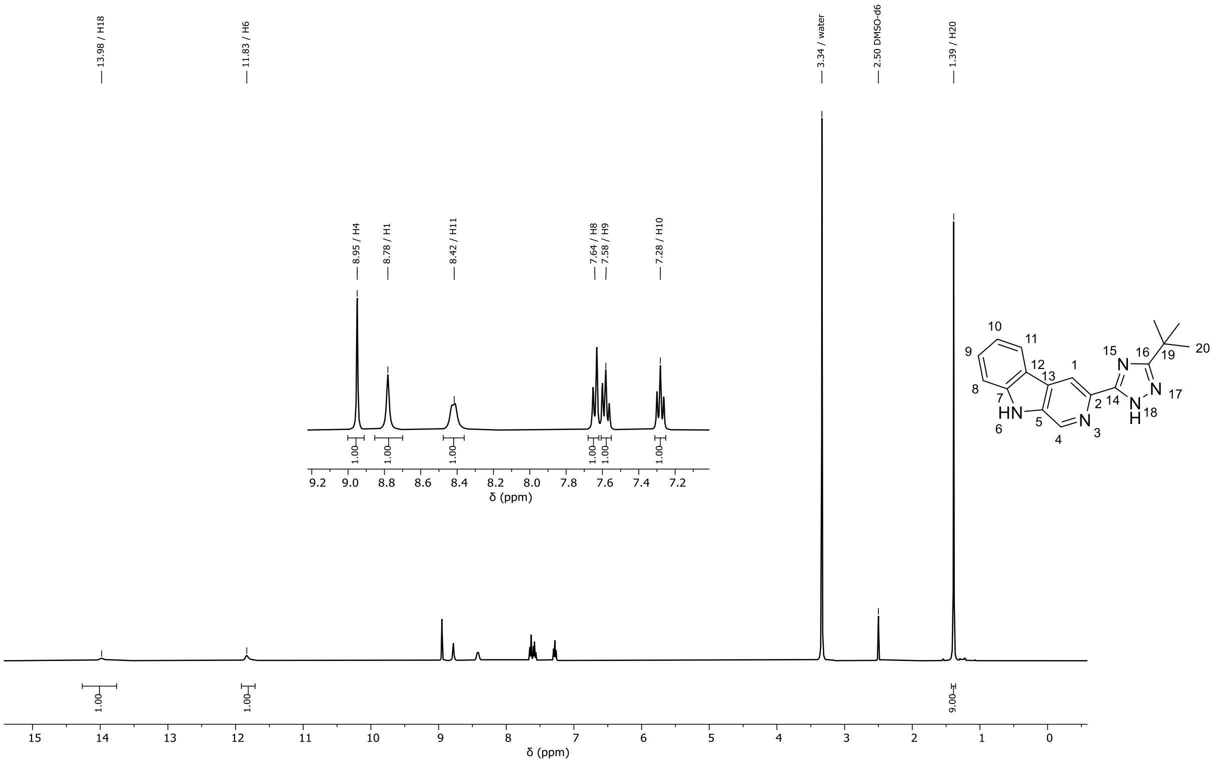

Figure S-13. ^{1}H-NMR spectrum (400 MHz, DMSO-d_6) of $\mathbf{L_{nHo}}$.

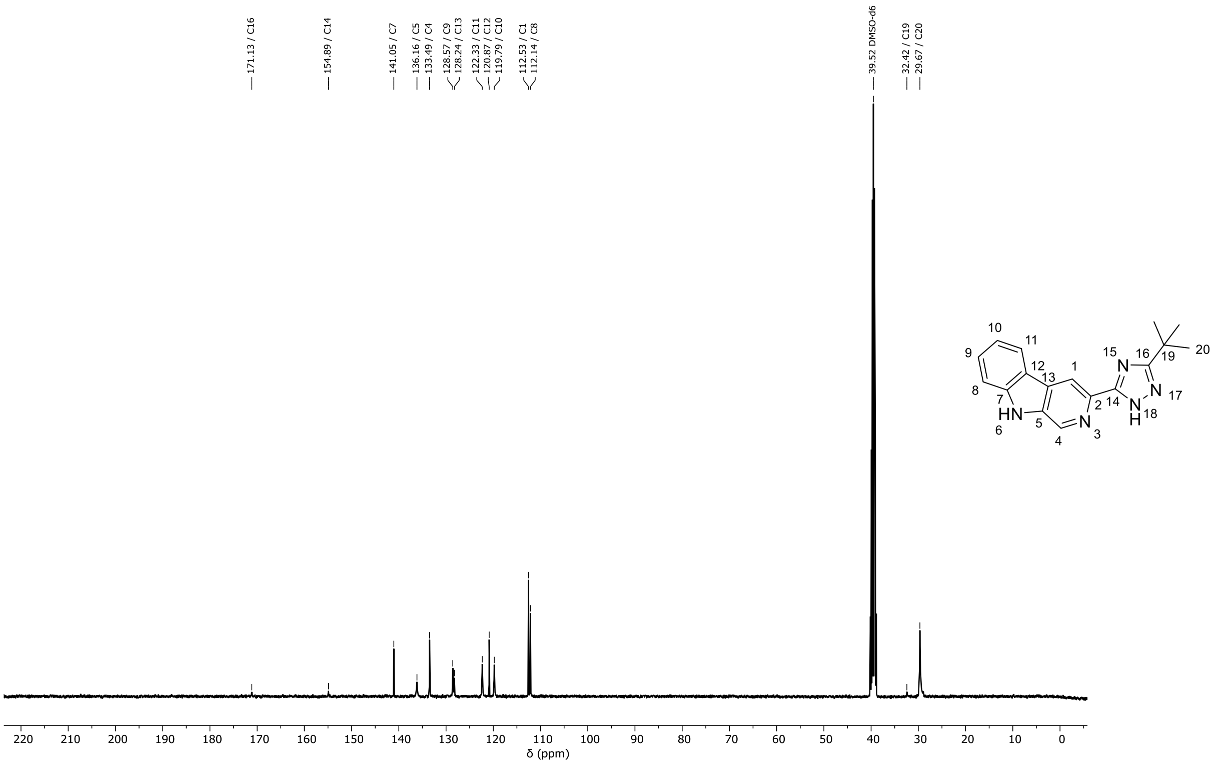

Figure S-14: ^{13}C{^{1}H}-NMR spectrum (101 MHz, DMSO-d_6) of $\mathbf{L_{nHo}}$.

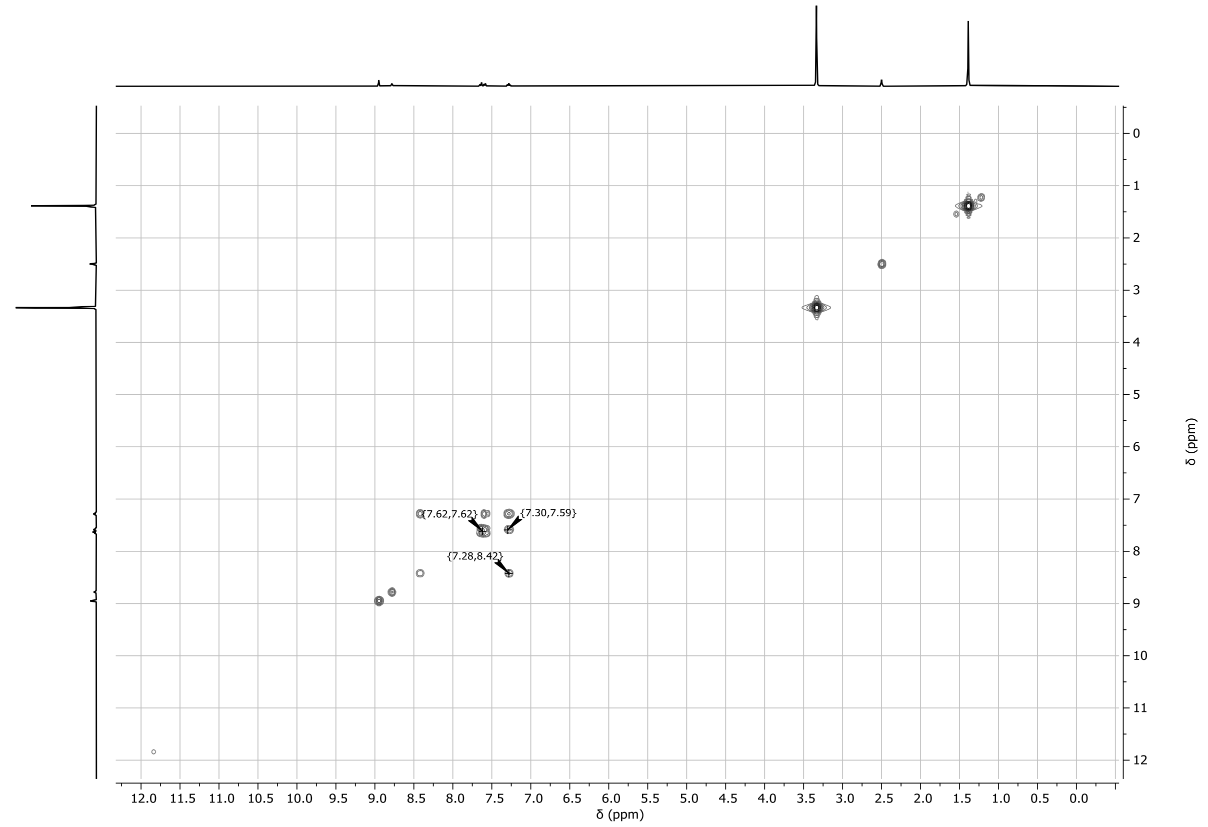

Figure S-15. HH-COSY-NMR spectrum (400 MHz, DMSO-d_6) of $\mathbf{L_{nHo}}$.

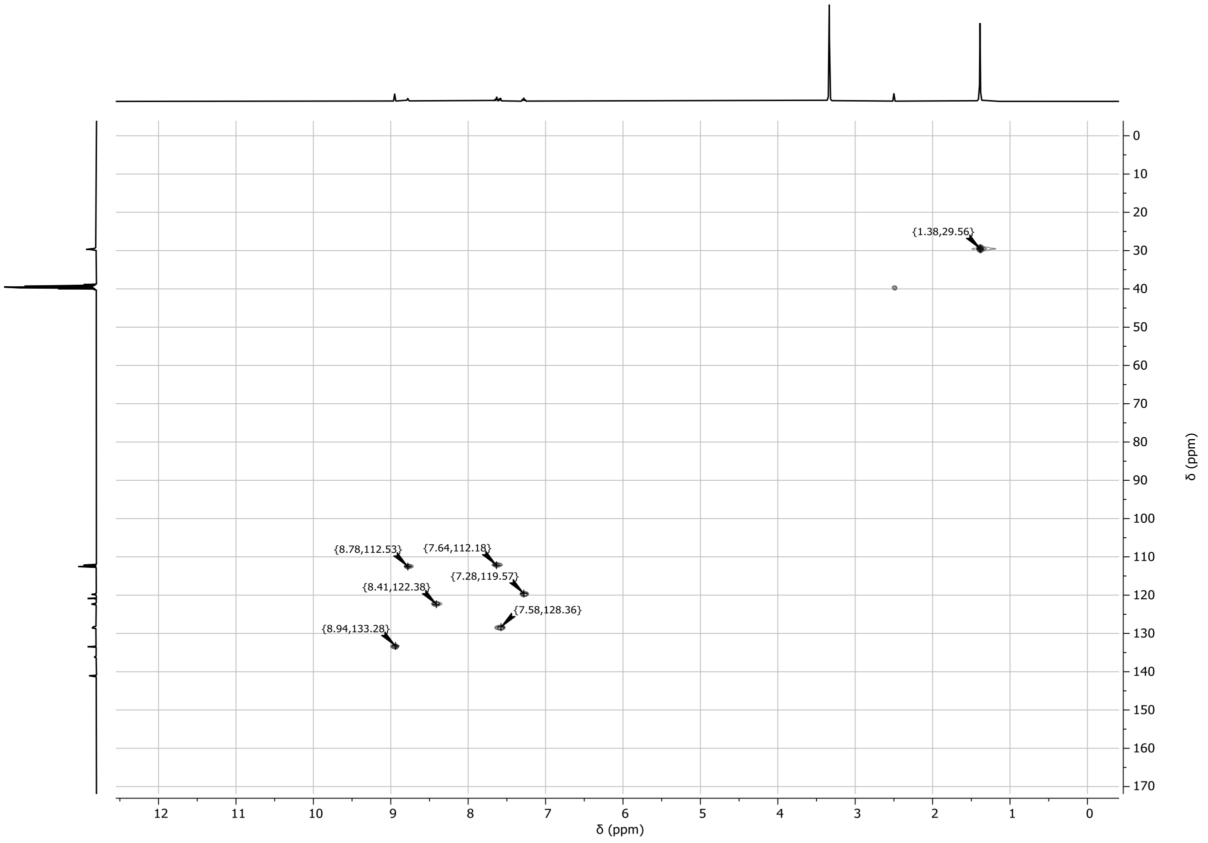

Figure S-16. HC-HSQC-NMR spectrum (101 MHz (^{13}C), 400 MHz (^{1}H), DMSO-d_6) of $\mathbf{L_{nHo}}$.

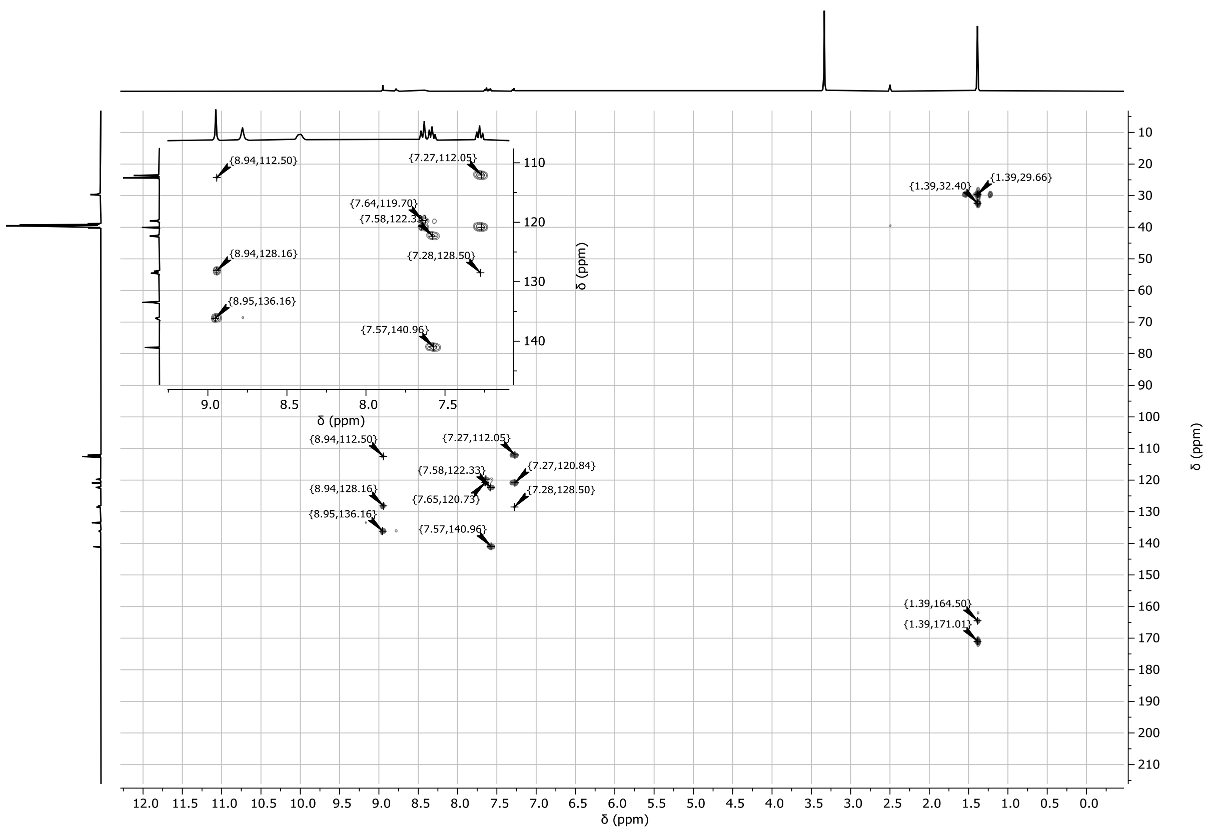

Figure S-17. HC-HMBC-NMR spectrum (101 MHz (^{13}C), 400 MHz (^{1}H), DMSO-d_6) of $\mathbf{L_{nHo}}$.

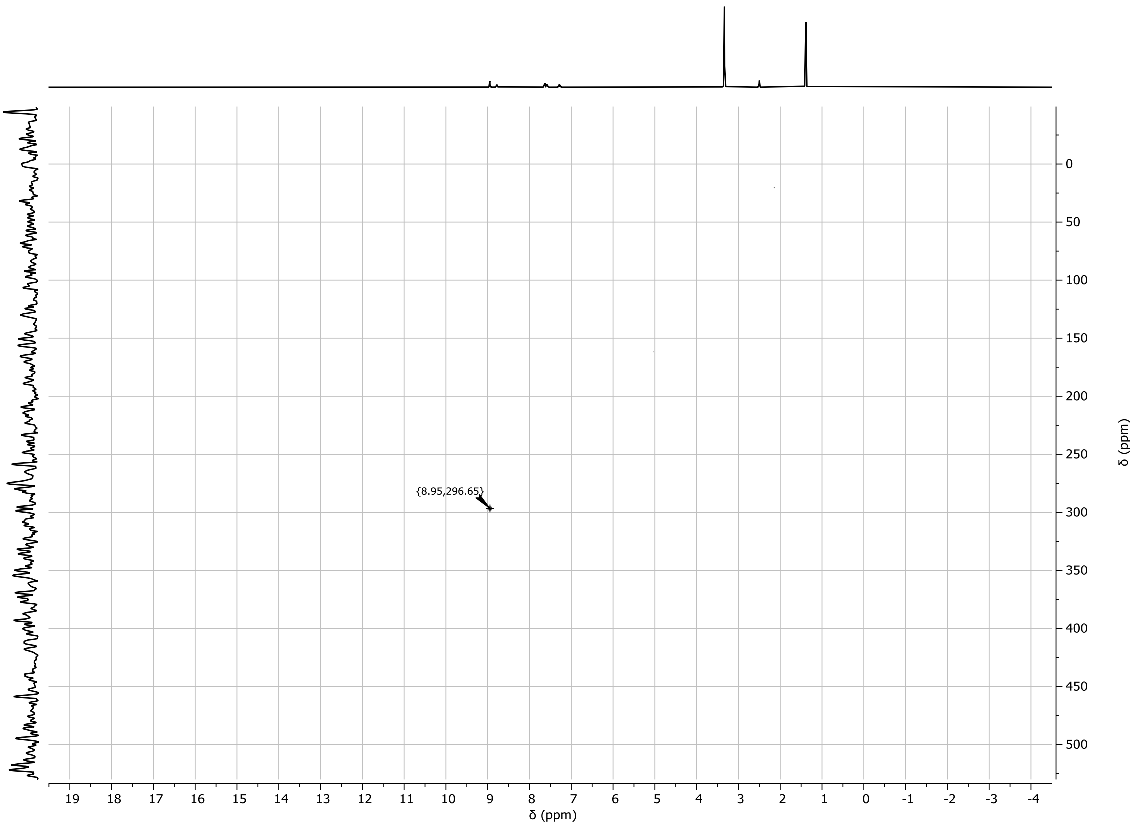

Figure S-18. HN-HMBC-NMR spectrum (41 MHz (^{13}C), 400 MHz (^{1}H), DCM-d_2) of $\mathbf{L_{nHo}}$.

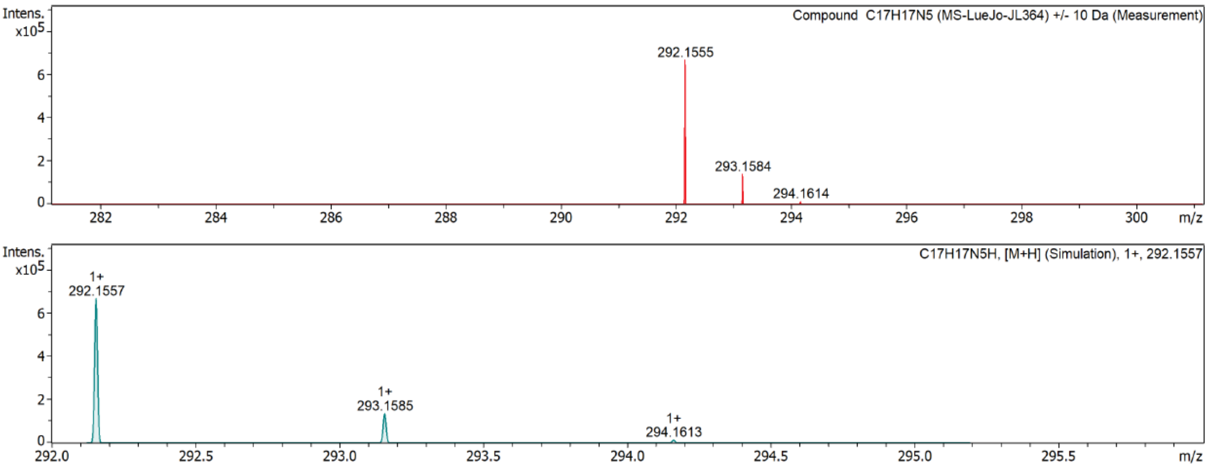

Figure S-19. Mass spectrum of $\mathbf{L_{nHo}}$ (MeOH). Additional simulation of the [$\mathbf{L_{nHo}}$+H]$^+$ adduct.

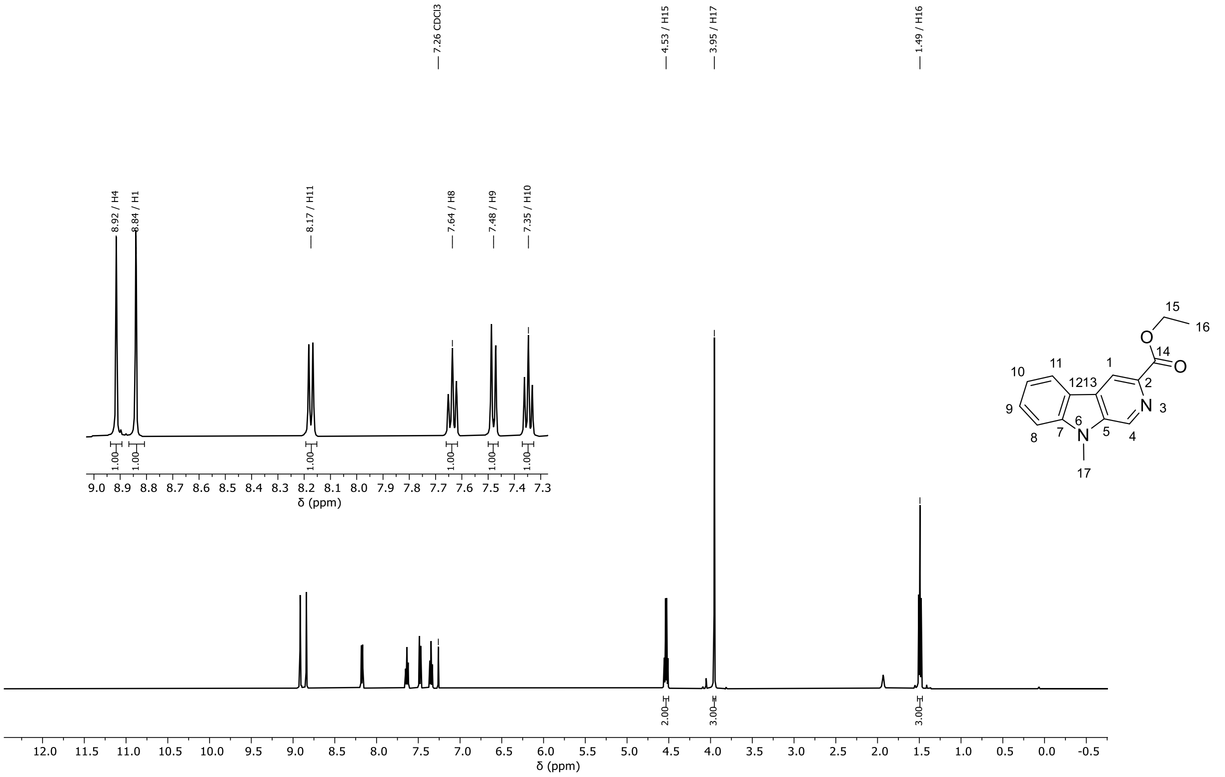

Figure S-20. ^{1}H-NMR spectrum (500 MHz, $CDCl_3$) of **5**.

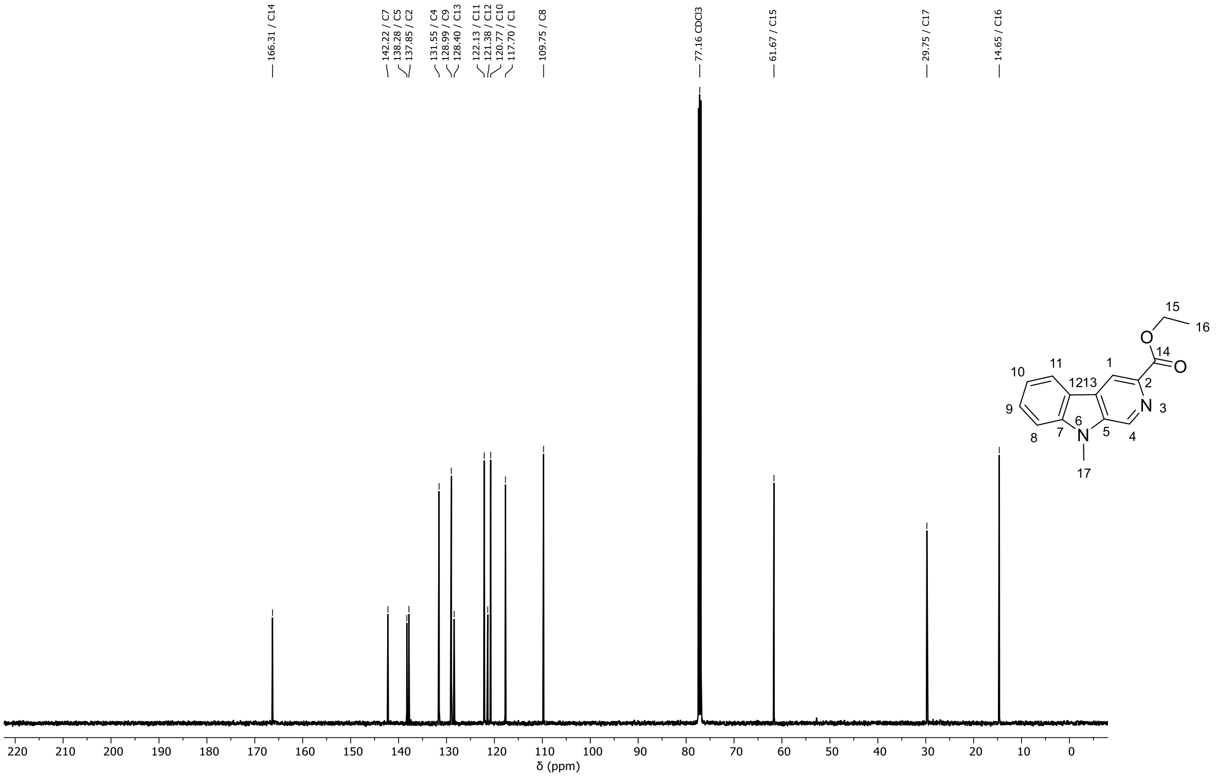

Figure S-21. ${}^{13}C\{{}^{1}H\}$-NMR spectrum (126 MHz, $CDCl_3$) of **5**.

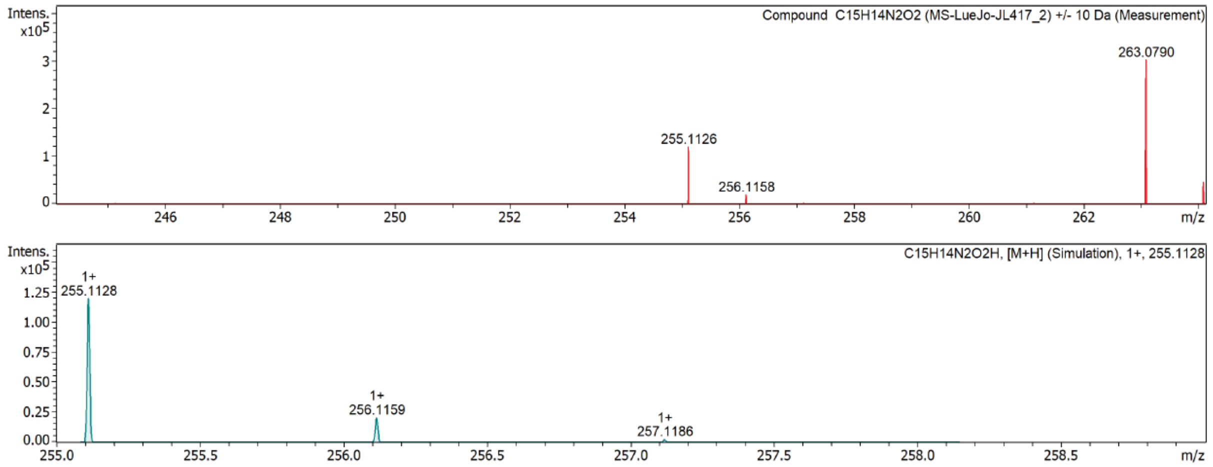

Figure S-22. Mass spectrum of **5** (MeOH). Additional simulation of the [**5**+H]$^+$ adduct.

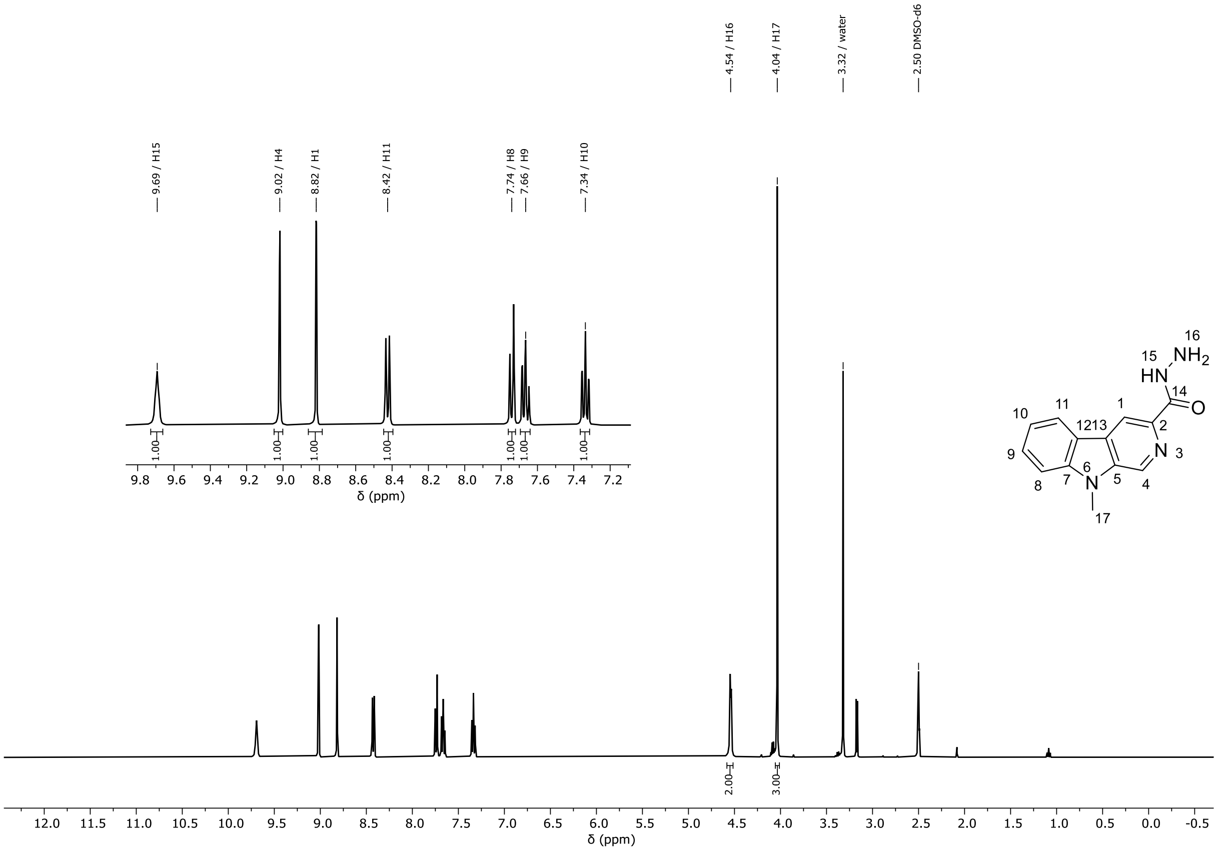

Figure S-23. ^{1}H-NMR spectrum (400 MHz, DMSO-d_6) of **6**.

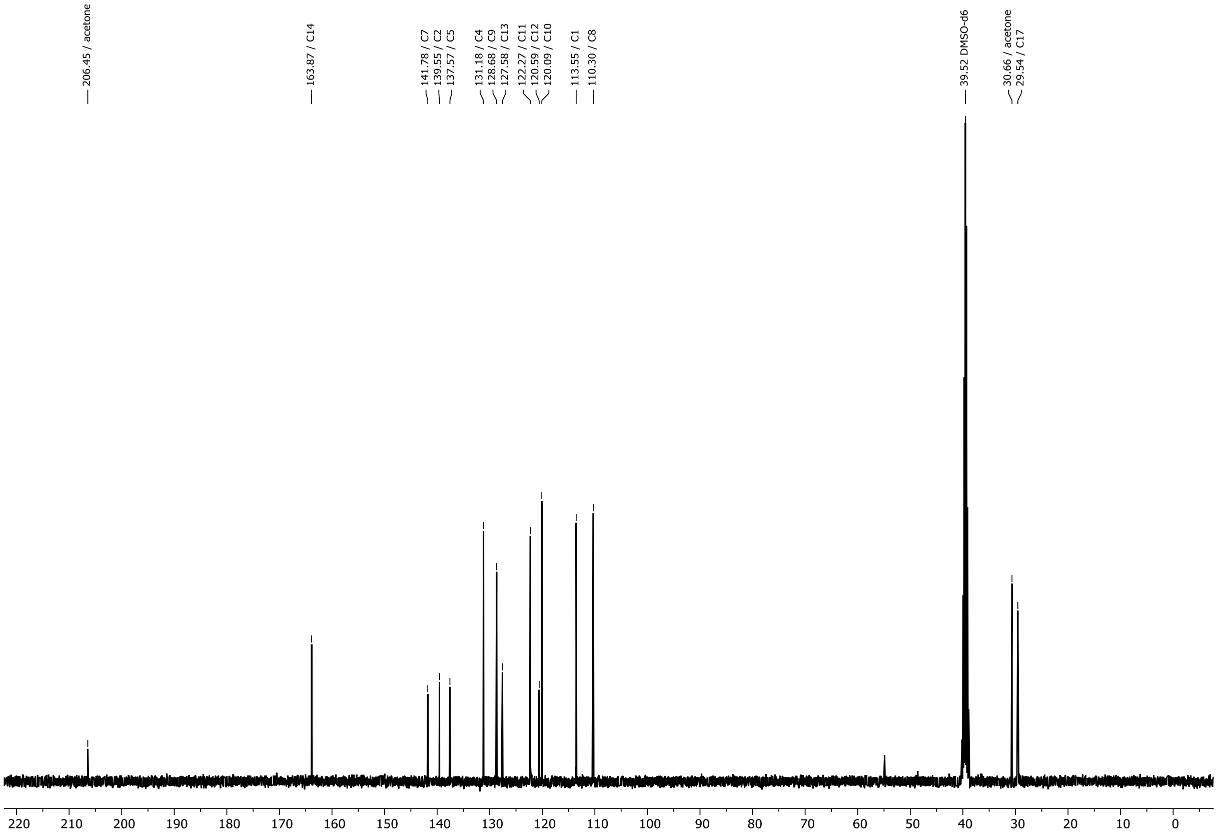

Figure S-24: ^{13}C{^{1}H}-NMR spectrum (101 MHz, DMSO-d_6) of **6**.

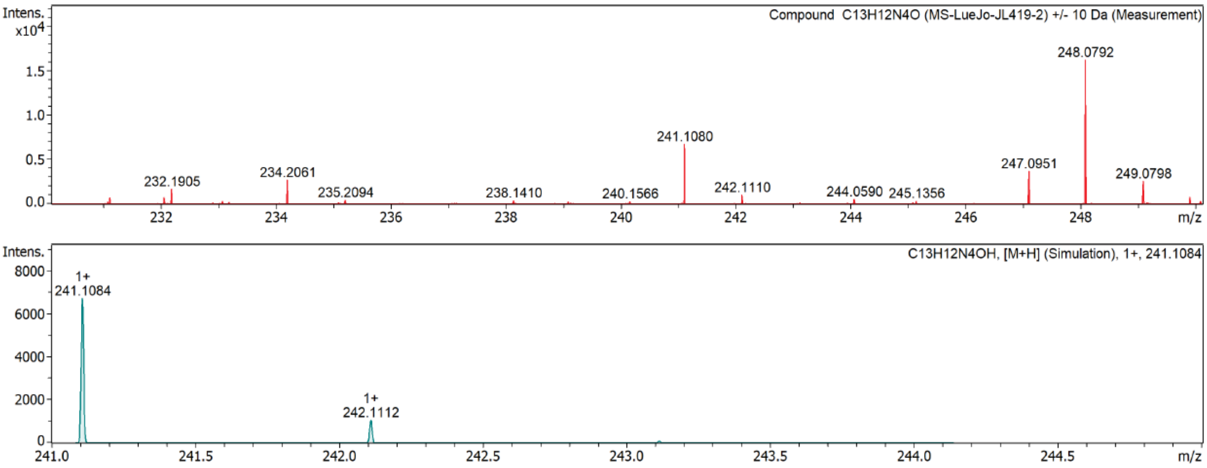

Figure S-25. Mass spectrum of **6** (MeOH). Additional simulation of the [**6**+H]$^{+}$ adduct.

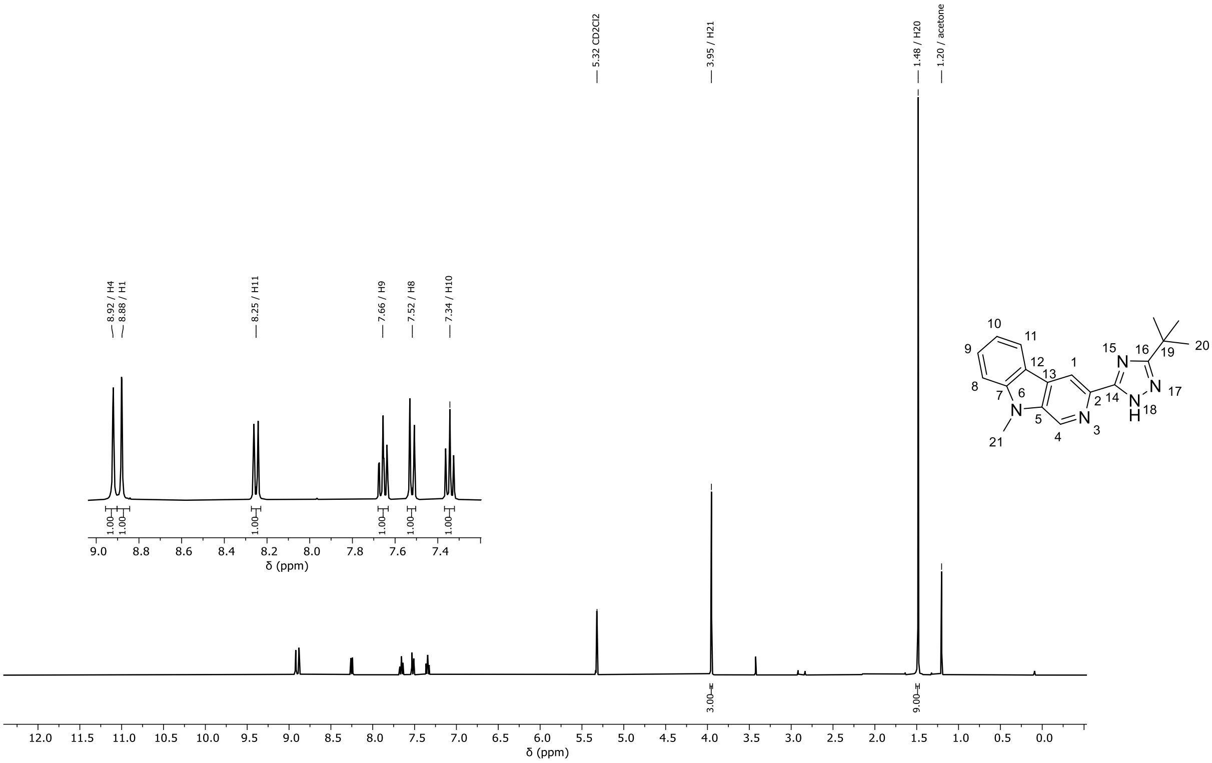

Figure S-26. ^{1}H-NMR spectrum (400 MHz, DMSO-d_6) of **L$_{\text{Me-nHo}}$**.

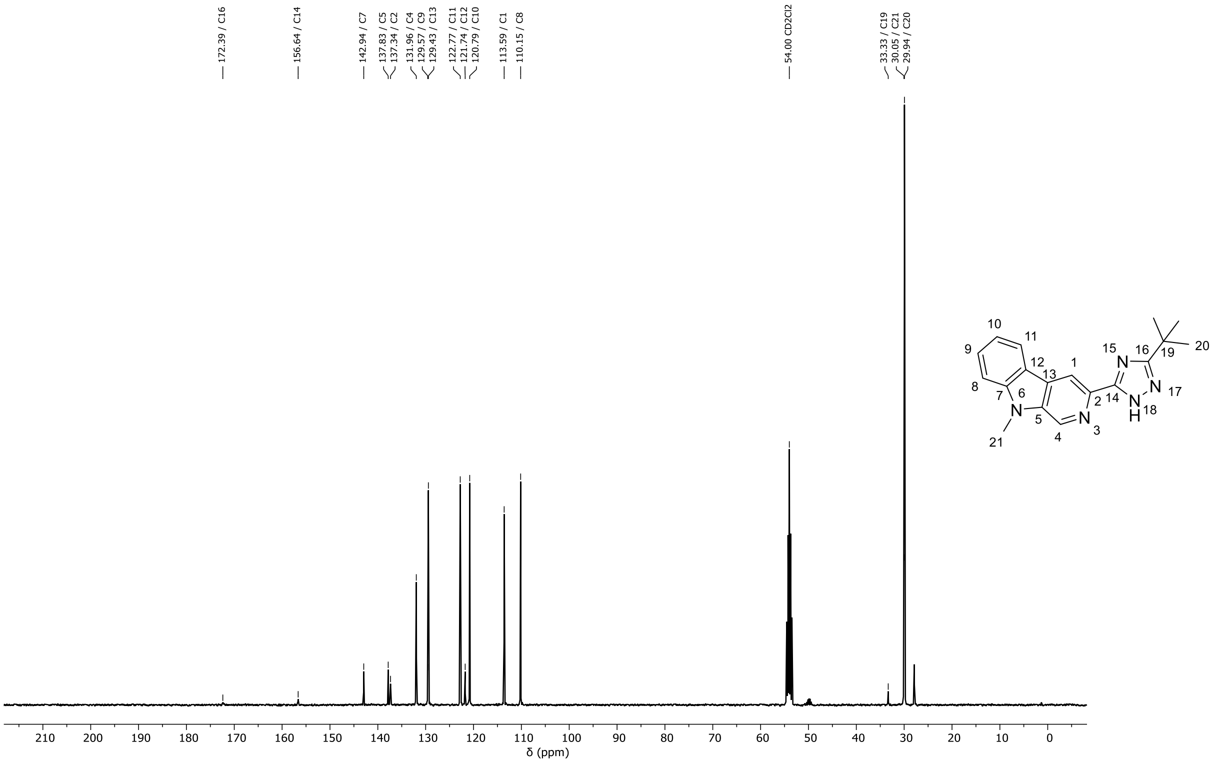

Figure S-27: $^{13}C\{^1H\}$-NMR spectrum (101 MHz, DMSO-d_6) of $\mathbf{L_{Me\text{-}nHo}}$.

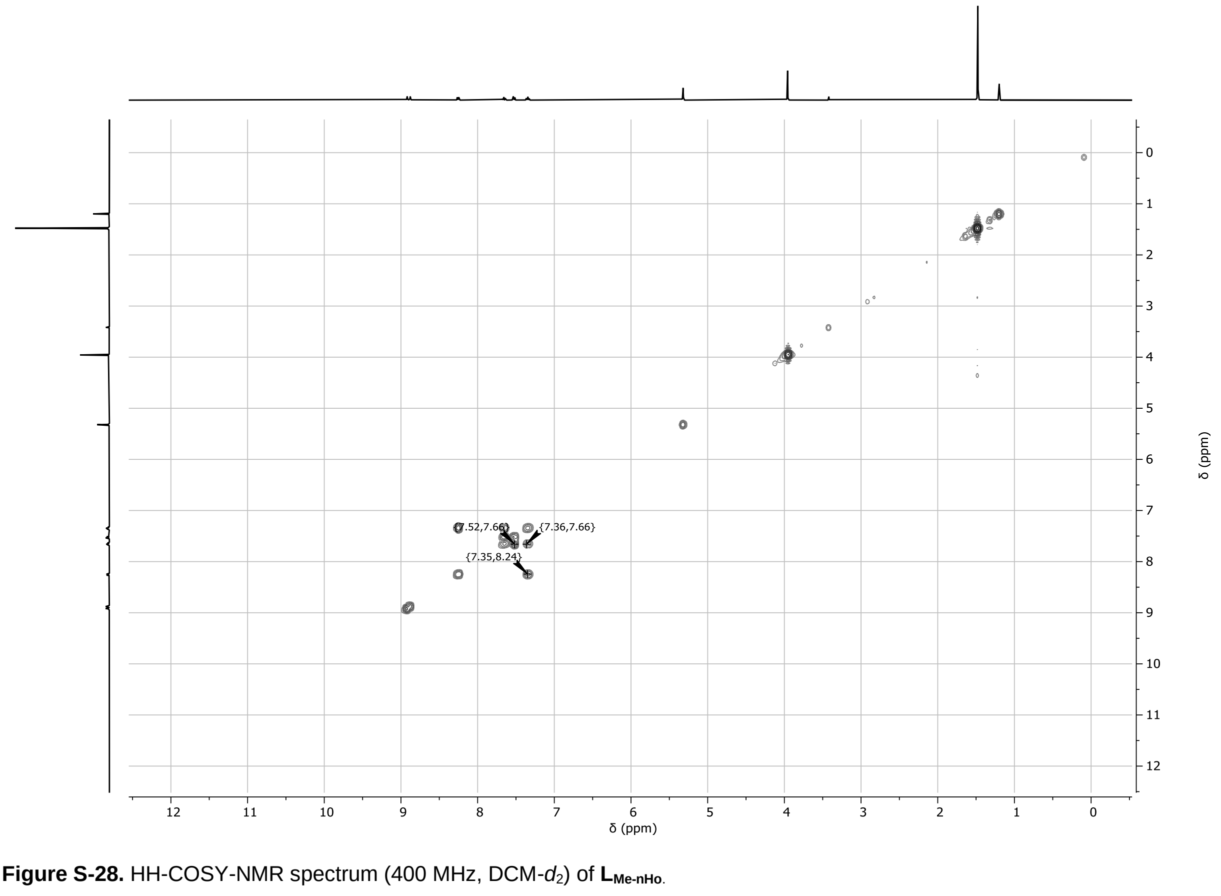

Figure S-28. HH-COSY-NMR spectrum (400 MHz, DCM-d_2) of $\mathbf{L_{Me\text{-}nHo}}$.

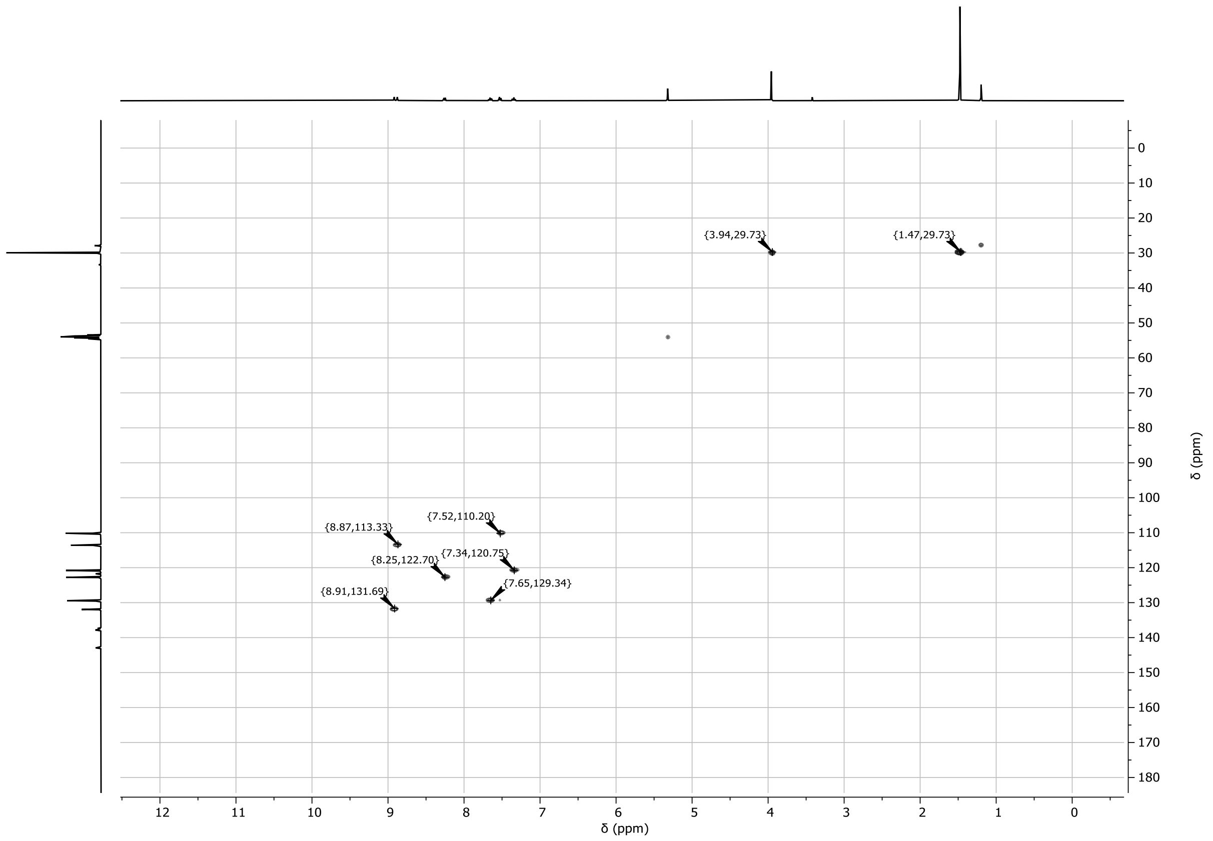

Figure S-29. HC-HSQC-NMR spectrum (101 MHz (^{13}C), 400 MHz (^{1}H), DCM-d_2) of $\mathbf{L}_{\mathbf{Me\text{-}nHo}}$.

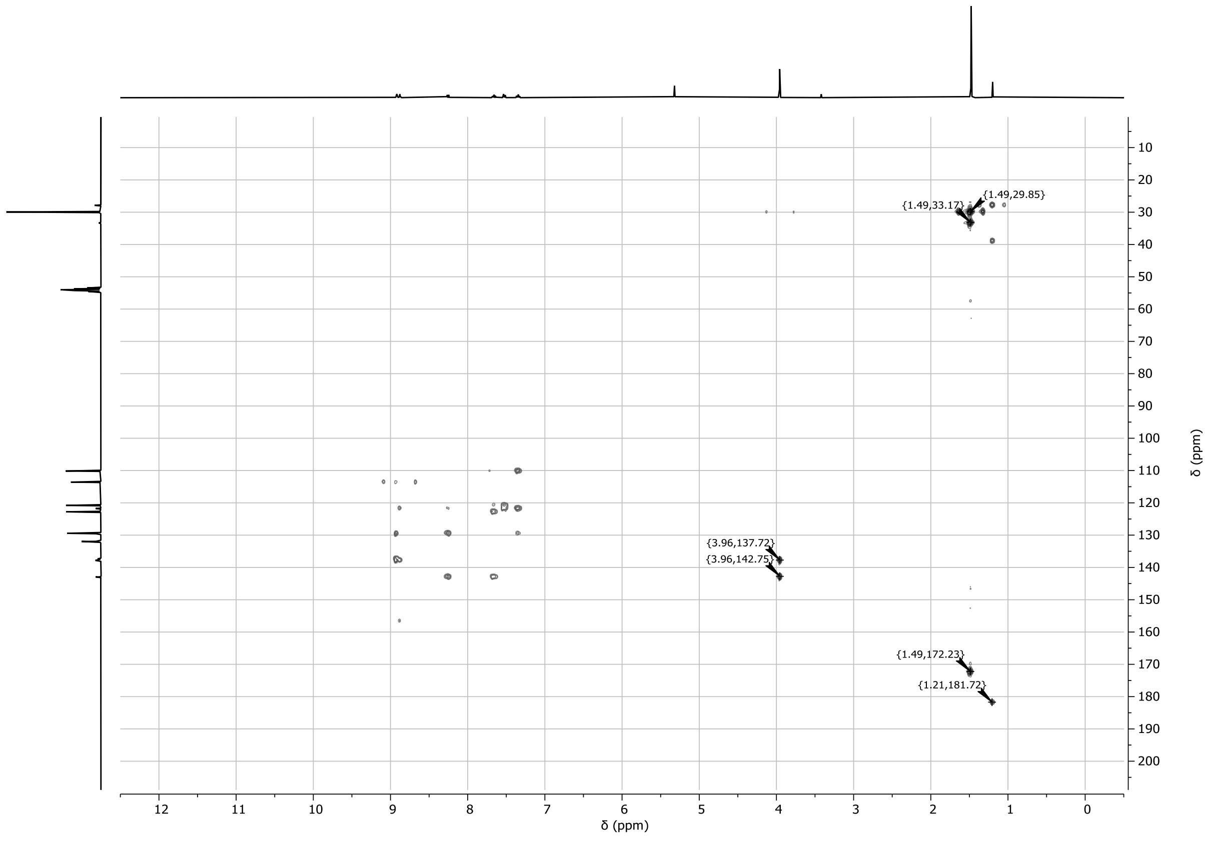

Figure S-30. HC-HMBC-NMR spectrum (101 MHz (^{13}C), 400 MHz (^{1}H), DCM-d_2) of $\mathbf{L}_{\mathbf{Me\text{-}nHo}}$.

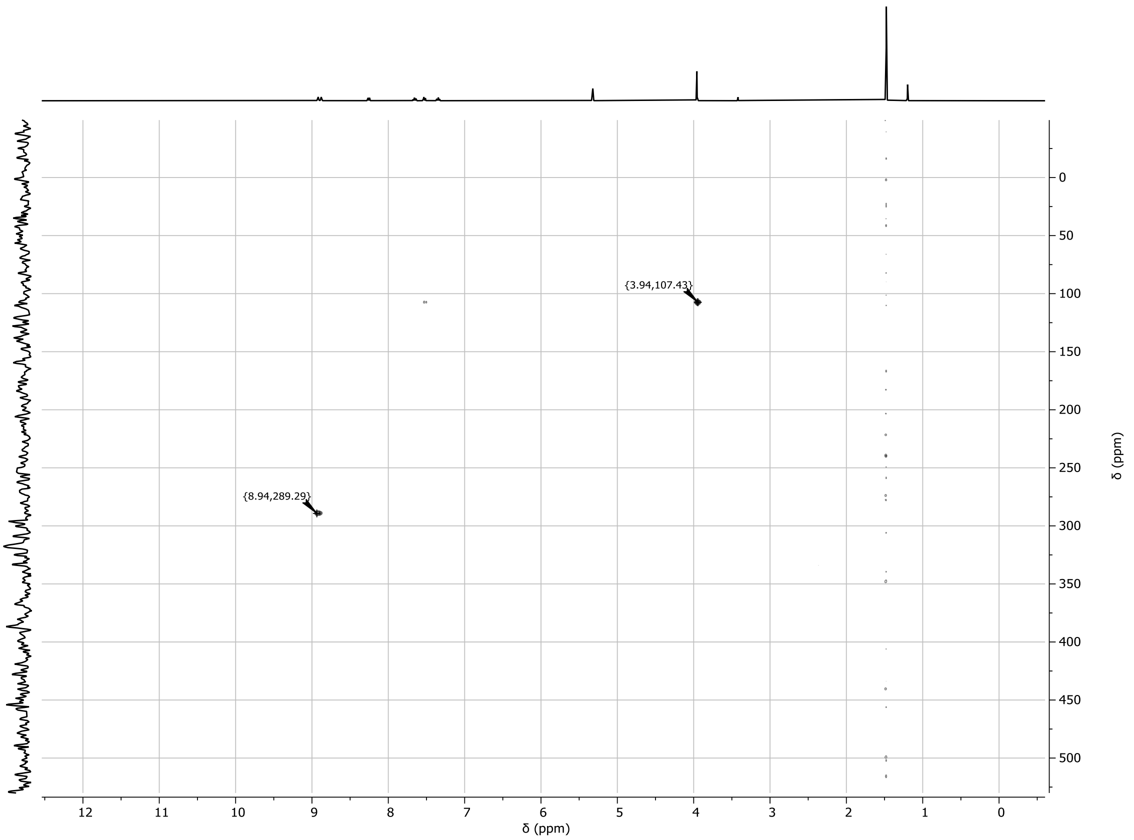

Figure S-31. HN-HMBC-NMR spectrum (41 MHz (^{13}C), 400 MHz (^{1}H), DCM-d_2) of $\mathbf{L}_{\mathbf{Me\text{-}nHo}}$.

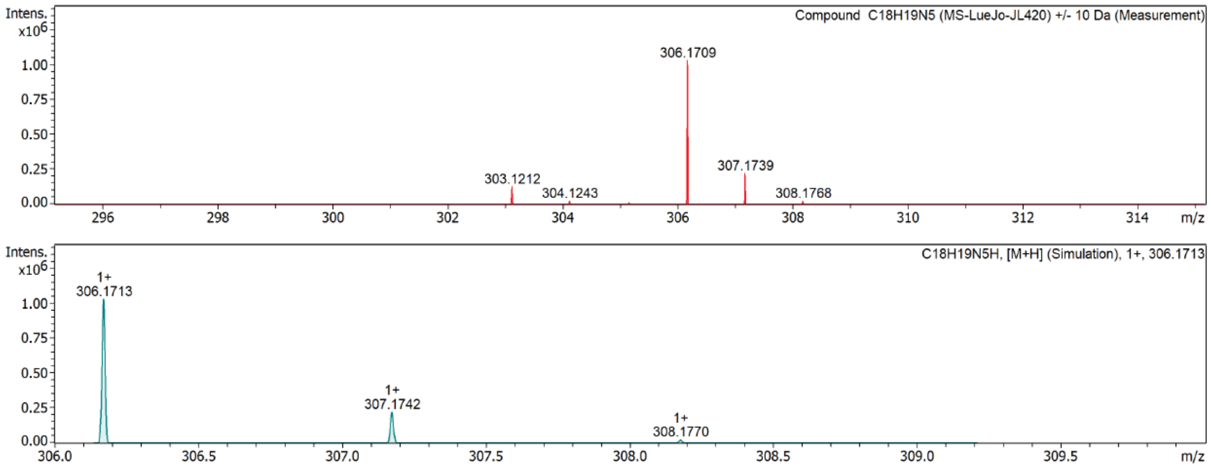

Figure S-32. Mass spectrum of $\mathbf{L}_{\mathbf{Me\text{-}nHo}}$ (MeOH). Additional simulation of the [$\mathbf{L}_{\mathbf{Me\text{-}nHo}}$+H]$^{+}$ adduct.

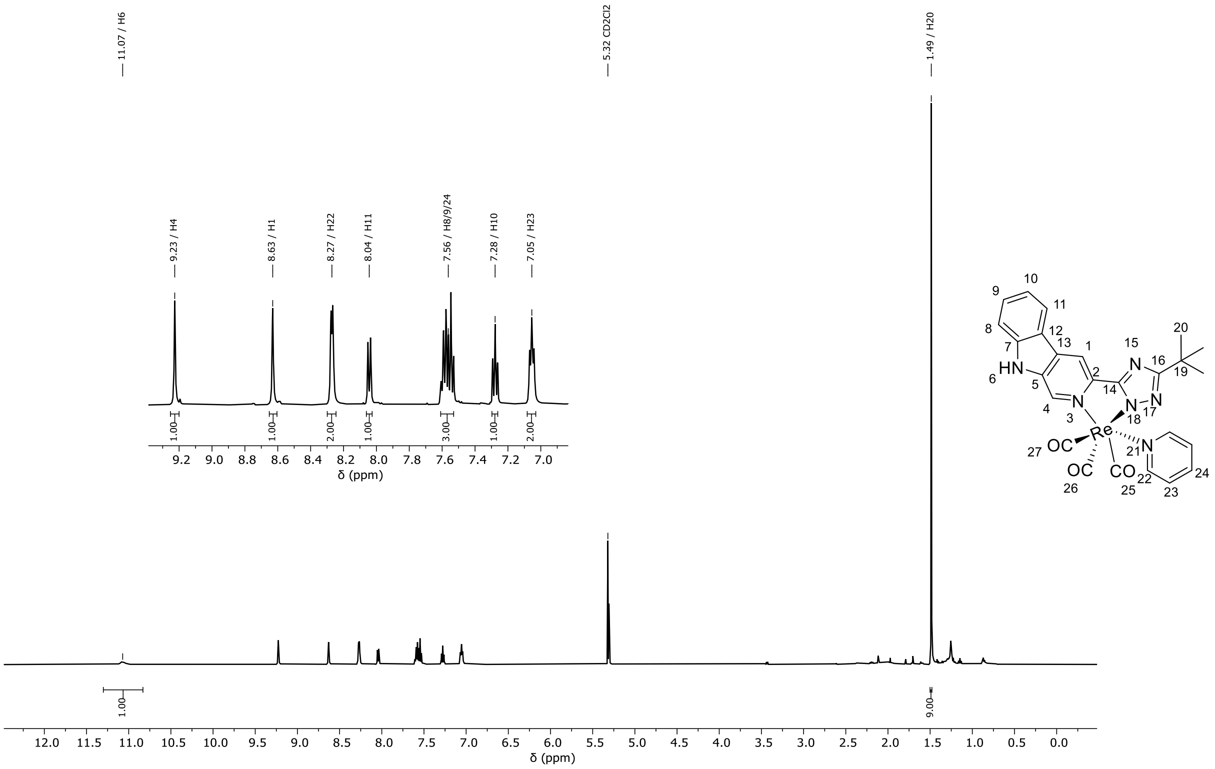

Figure S-33. ^{1}H-NMR spectrum (500 MHz, DCM-d_2) of **Re(L$_{nHo}$)Py**.

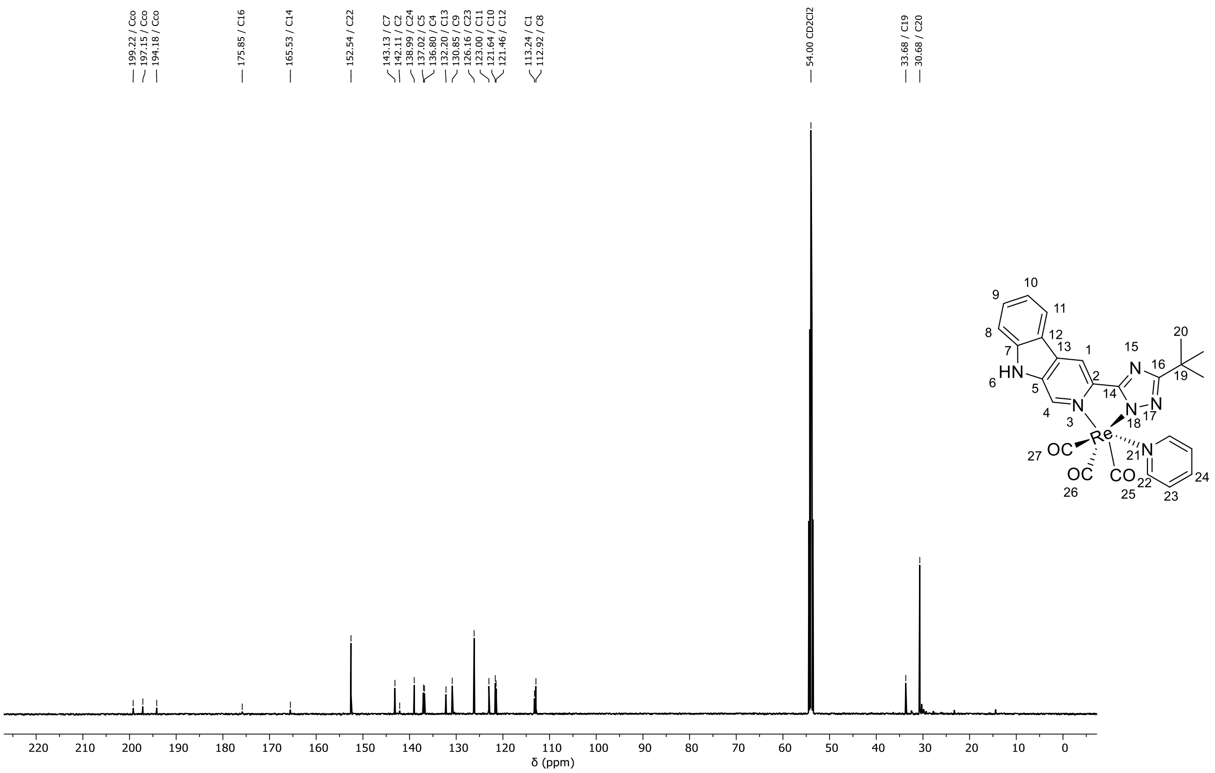

Figure S-34: ^{13}C{^{1}H}-NMR spectrum (126 MHz, DCM-d_2) of **Re(L$_{nHo}$)Py**.

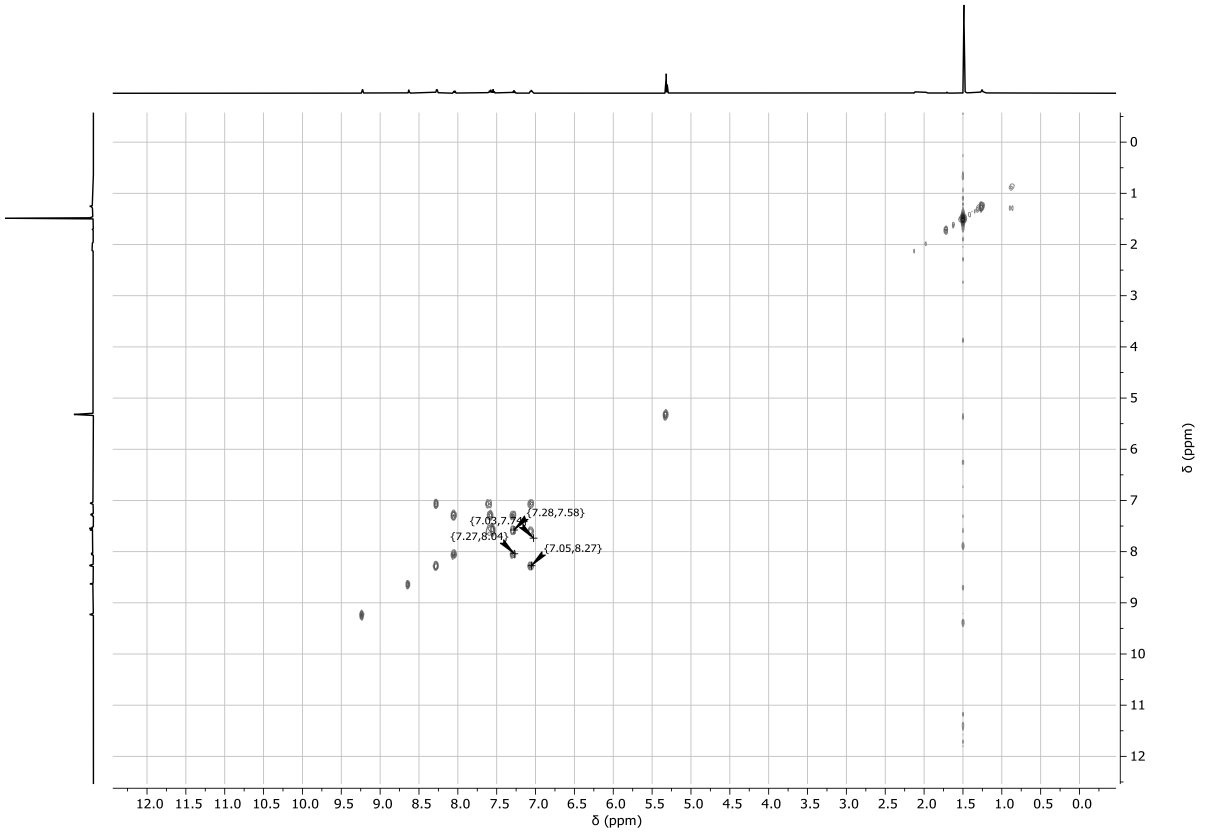

Figure S-35. HH-COSY-NMR spectrum (500 MHz, DCM-d_2) of **Re(L_{nHo})Py**.

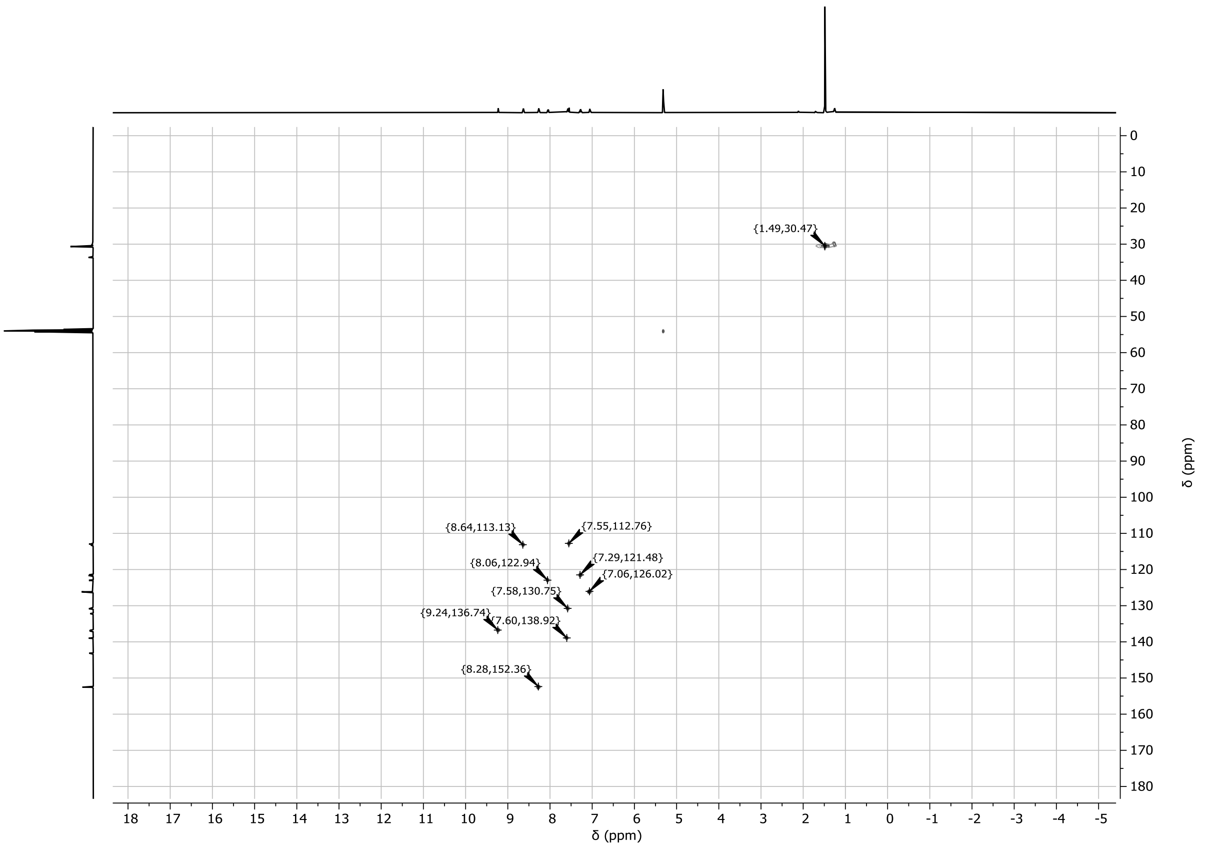

Figure S-36. HC-HSQC-NMR spectrum (126 MHz (^{13}C), 500 MHz (^{1}H), DCM-d_2) of **Re(L_{nHo})Py**.

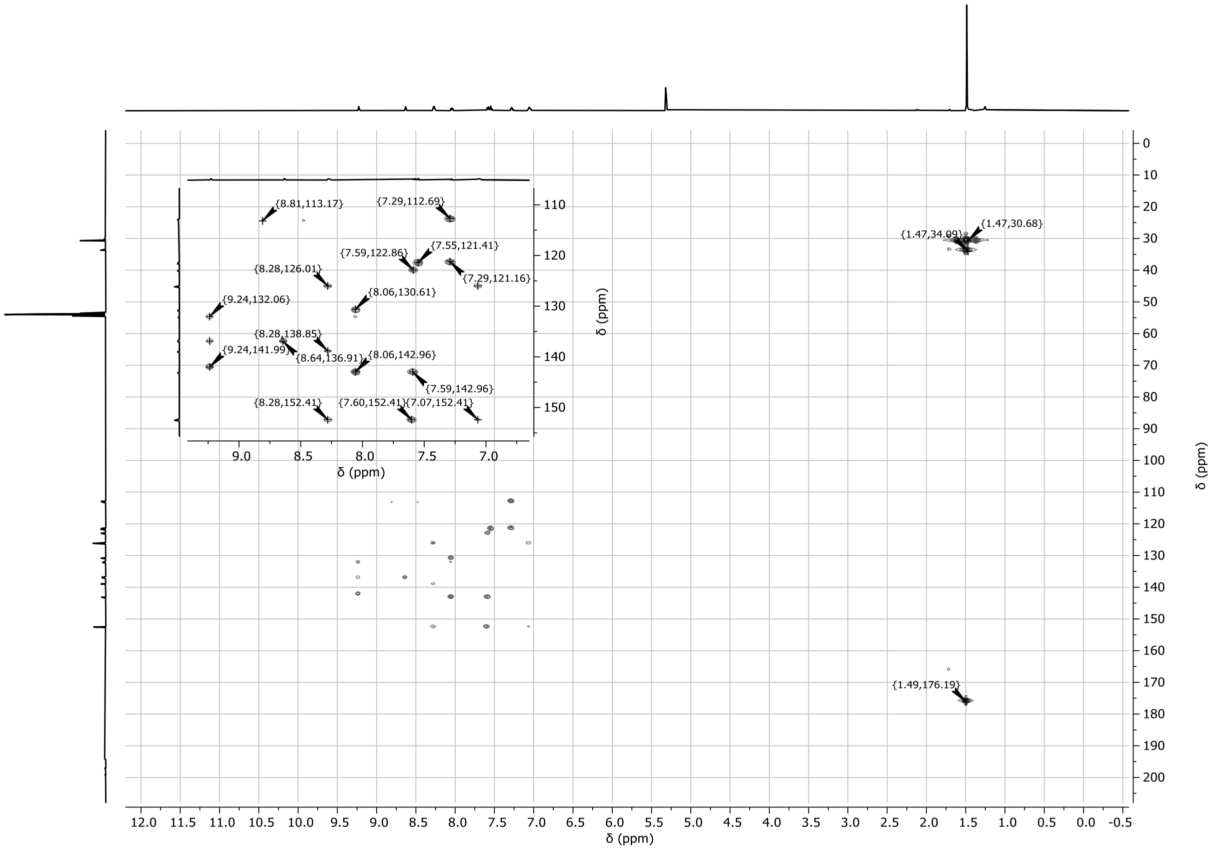

Figure S-37. HC-HMBC-NMR spectrum (126 MHz (^{13}C), 500 MHz (^{1}H), DCM-d_2) of **Re(L$_{nHo}$)Py**.

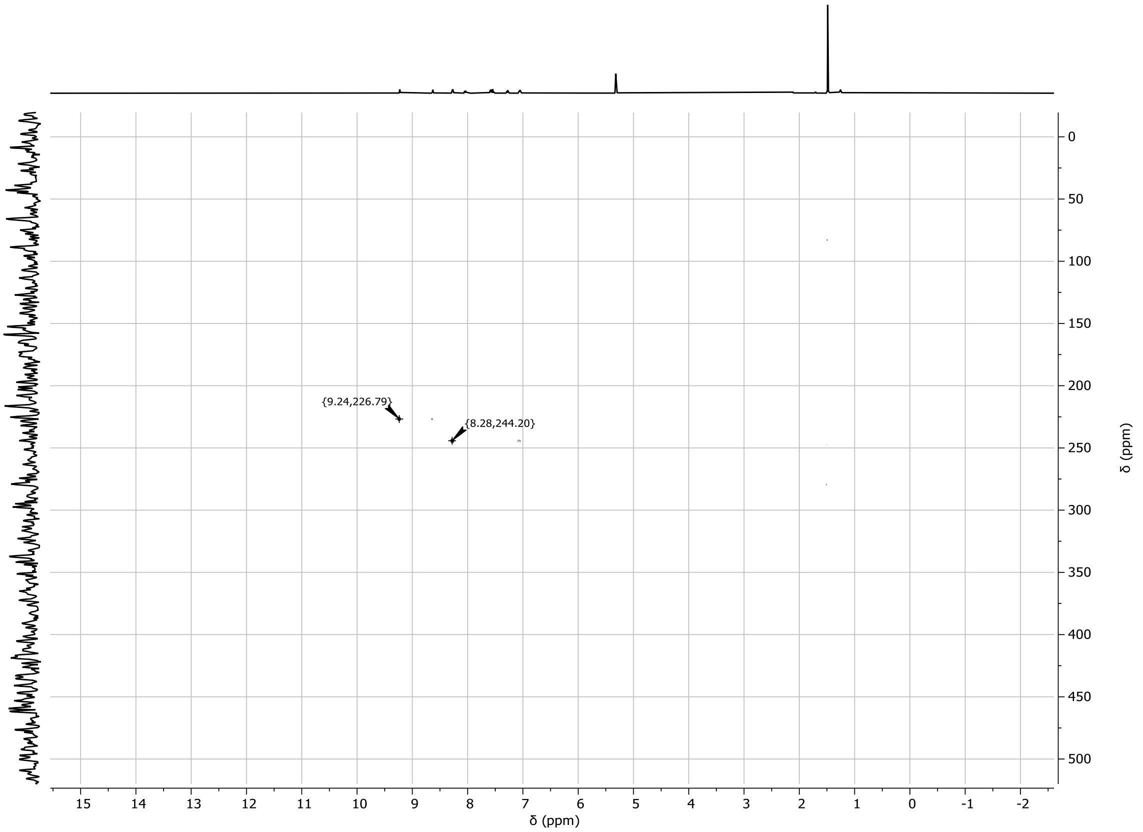

Figure S-38. HN-HMBC-NMR spectrum (51 MHz (^{15}N), 500 MHz (^{1}H), DCM-d_2) of **Re(L$_{nHo}$)Py**.

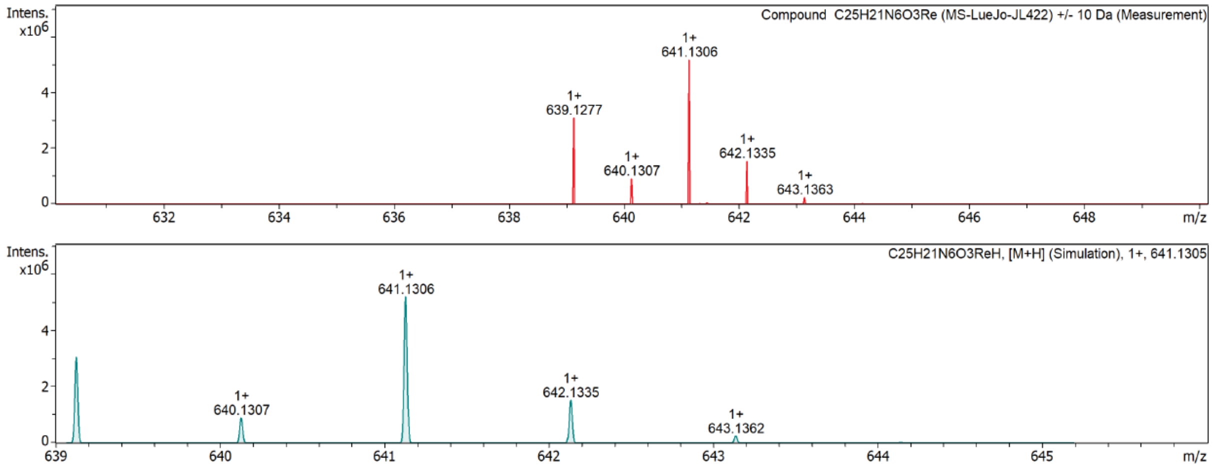

Figure S-39. Mass spectrum of **Re(L$_{nHo}$)Py** (MeOH). Additional simulation of the [**Re(L$_{nHo}$)Py**+H]$^+$ adduct.

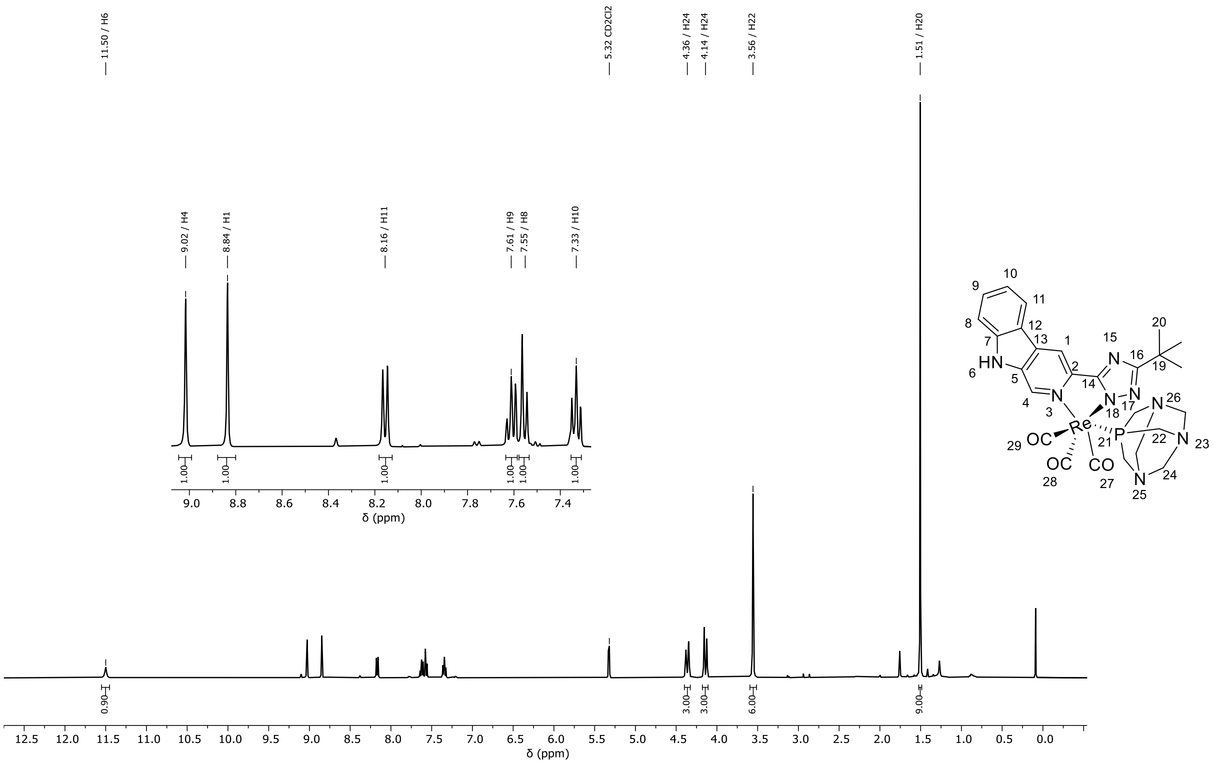

Figure S 40. ^{1}H-NMR spectrum (400 MHz, DCM-d_2) of **Re(L$_{nHo}$)PTA**.

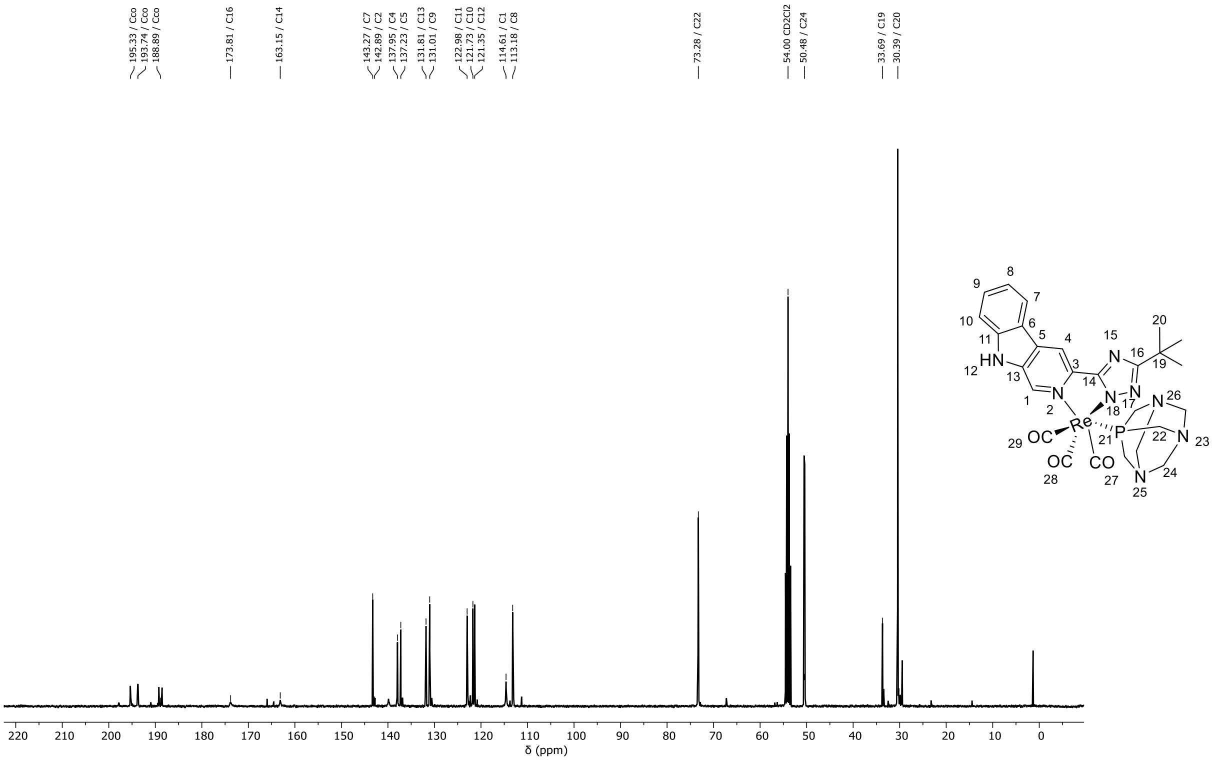

Figure S 41. $^{13}C\{^{1}H\}$-NMR spectrum (101 MHz, DCM-d_2) of **Re(L$_{nHo}$)PTA**.

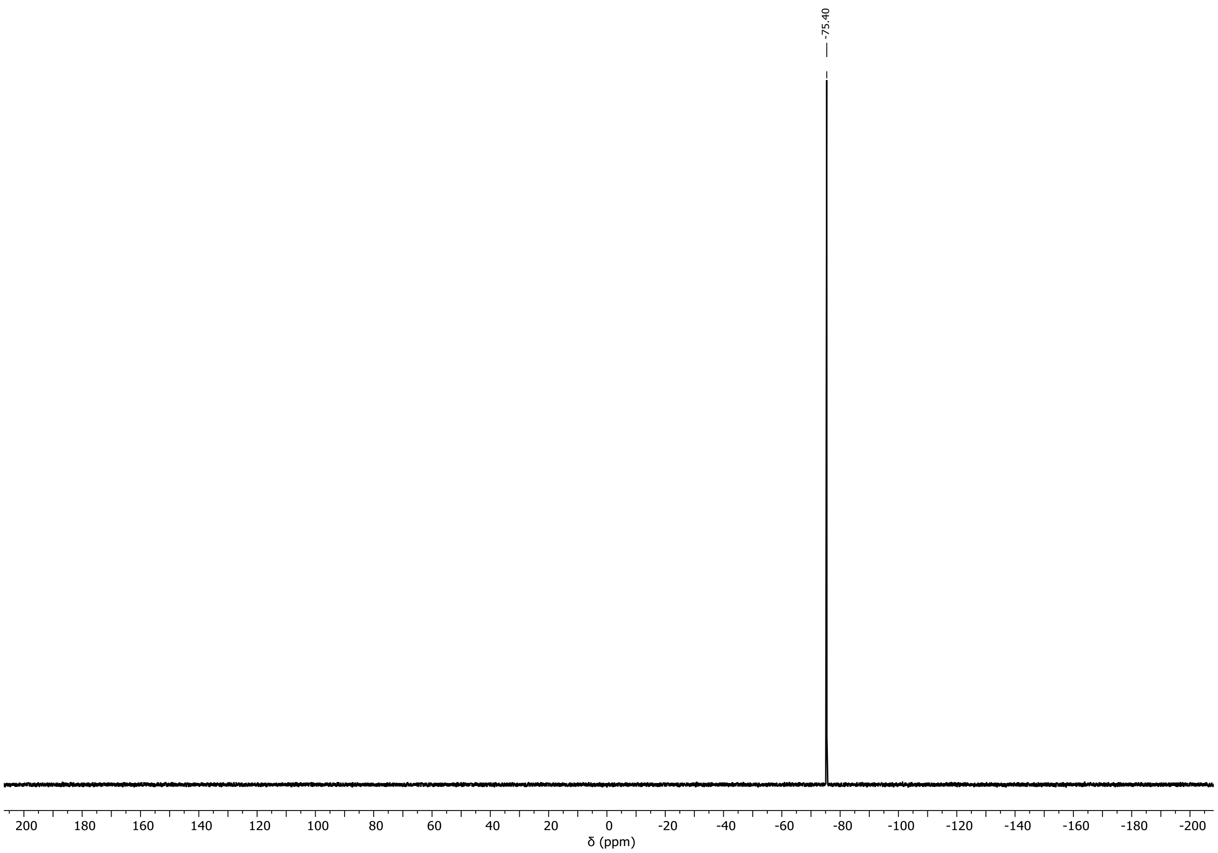

Figure S 42. $^{31}P\{^{1}H\}$-NMR spectrum (162 MHz, DCM-d_2) of **Re(L$_{nHo}$)PTA**.

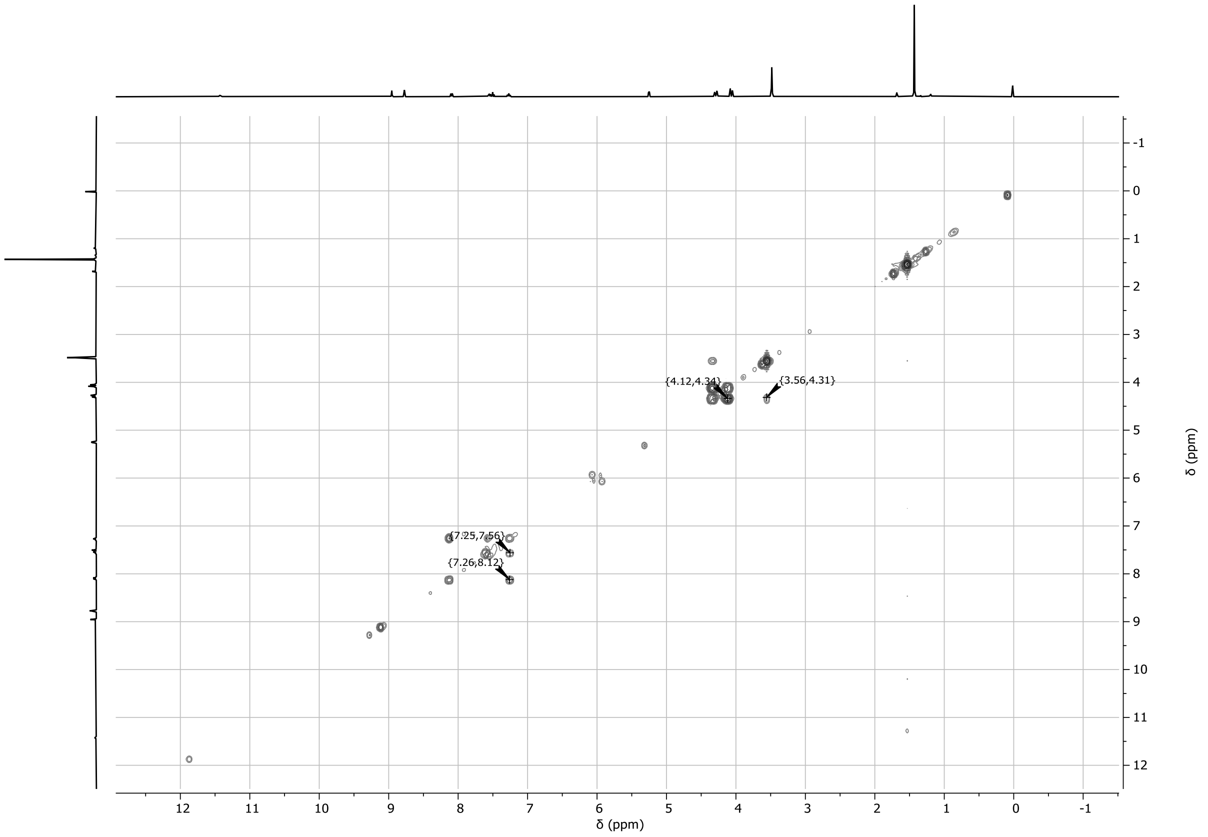

Figure S 43. HH-COSY-NMR spectrum (400 MHz, DCM-d_2) of **Re(L_{nHo})PTA**.

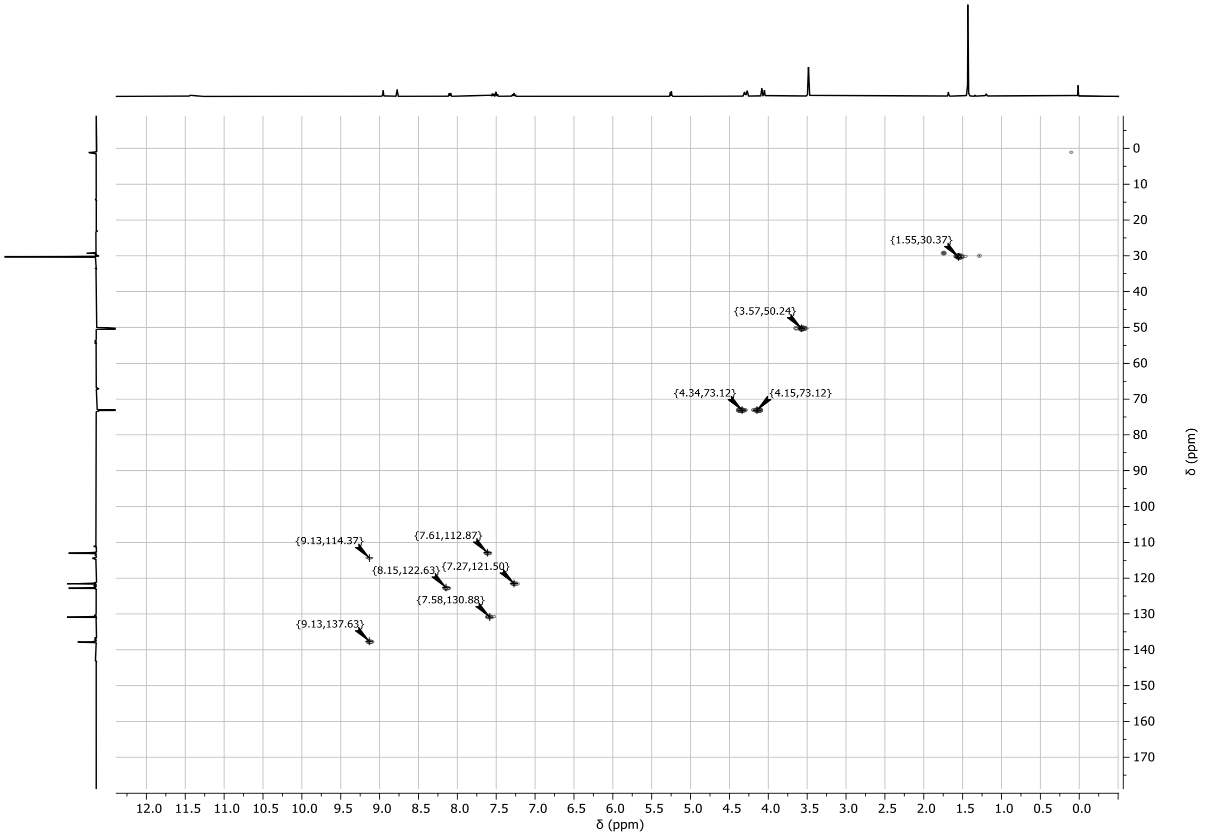

Figure S 44. HC-HSQC-NMR spectrum (101 MHz (^{13}C), 400 MHz (^{1}H), DCM-d_2) of **Re(L_{nHo})PTA**.

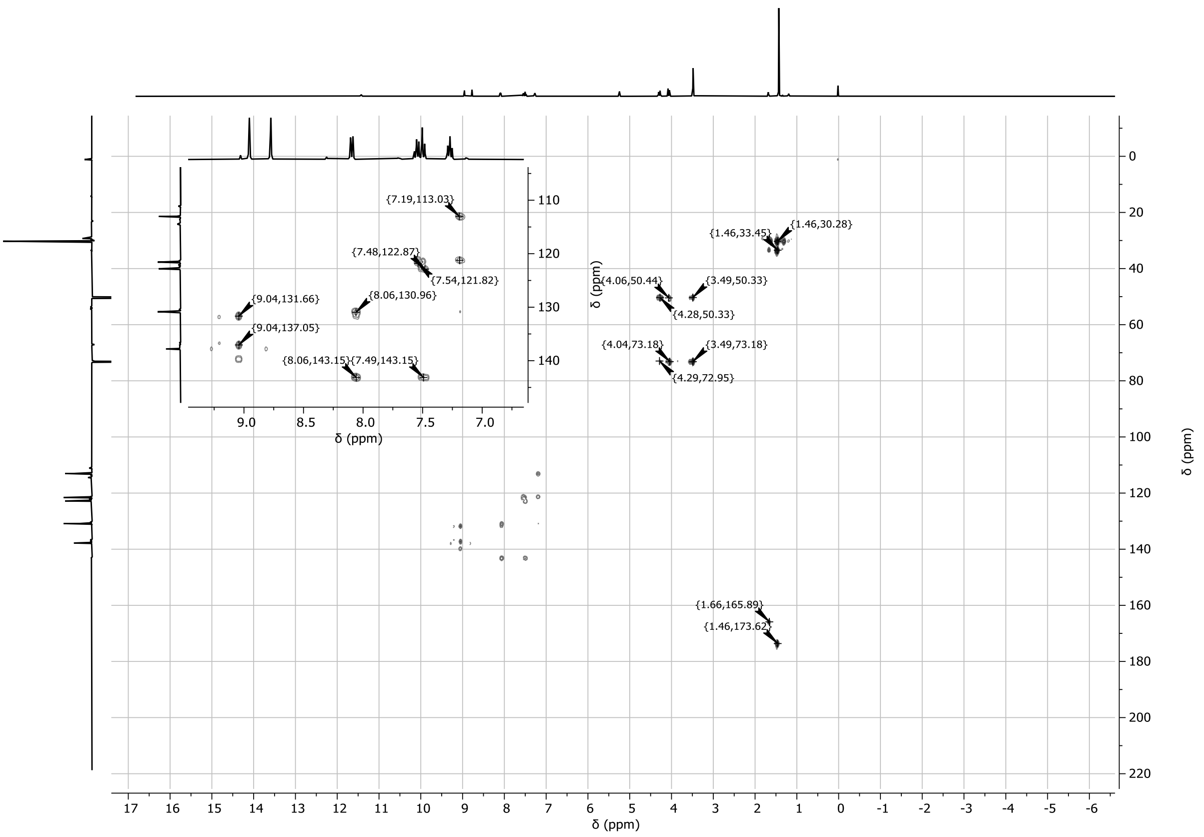

Figure S 45. HC-HMBC-NMR spectrum (101 MHz (^{13}C), 400 MHz (^{1}H), DCM-d_2) of **Re(L$_{nHo}$)PTA**.

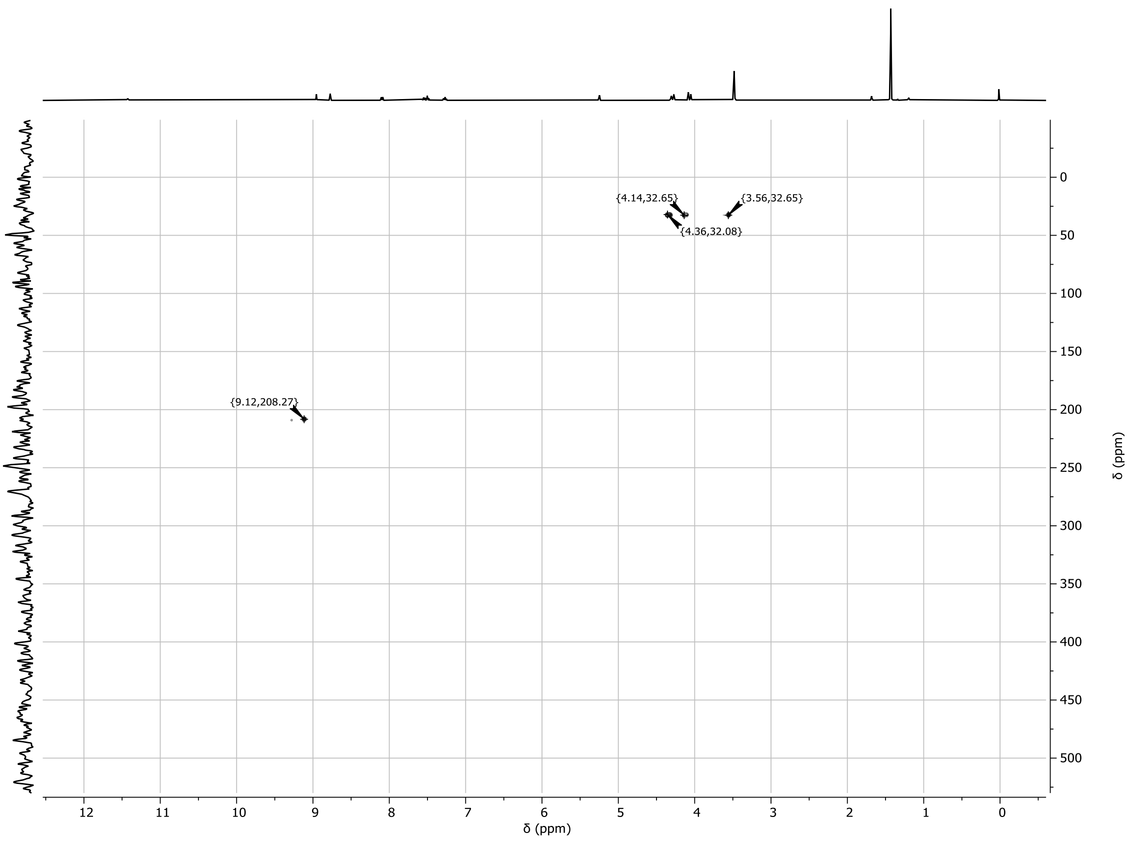

Figure S 46. HN-HMBC-NMR spectrum (41 MHz (^{15}N), 400 MHz (^{1}H), DCM-d_2) of **Re(L$_{nHo}$)PTA**.

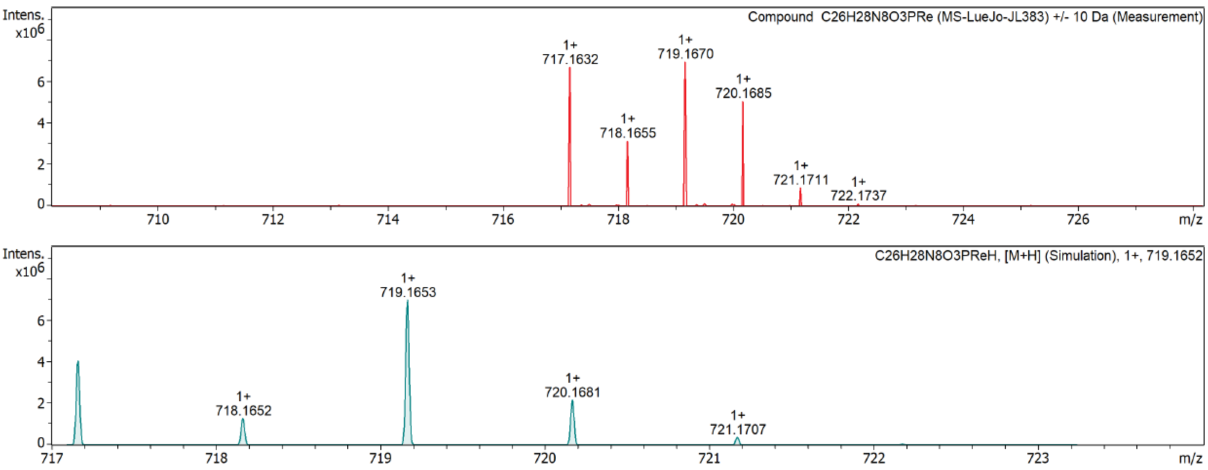

Figure S 47. Mass spectrum of **Re(L$_{nHo}$)PTA** (MeOH). Additional simulation of the [**Re(L$_{nHo}$)PTA**+H]$^{+}$ adduct.

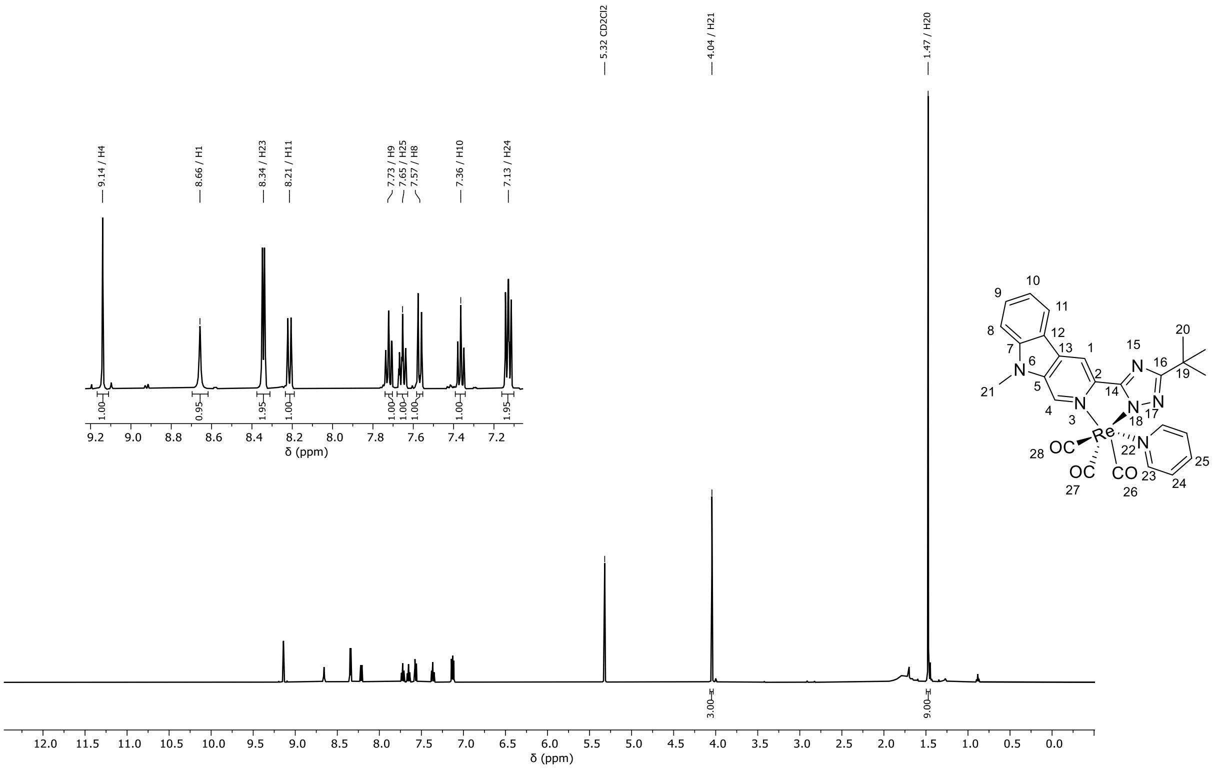

Figure S 48. ^{1}H-NMR spectrum (500 MHz, DCM-d_2) of **Re(L$_{Me\text{-}nHo}$)Py**.

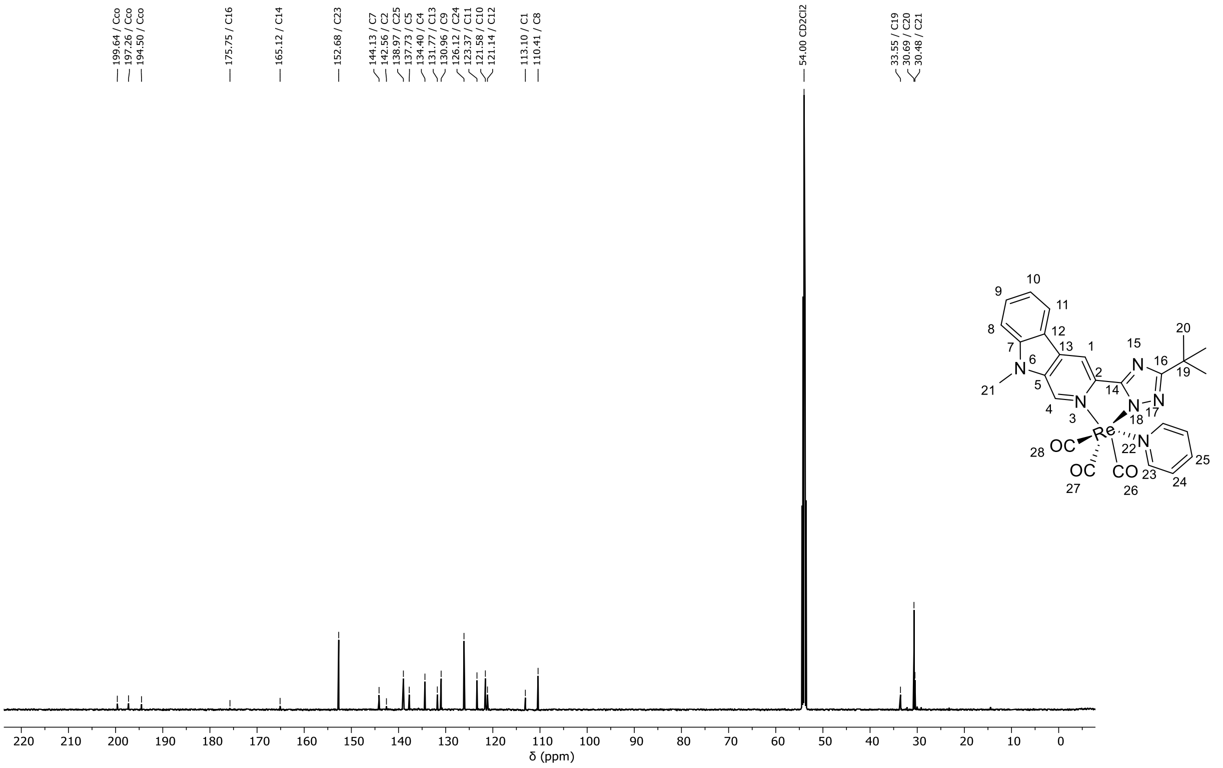

Figure S 49. $^{13}C\{^{1}H\}$-NMR spectrum (126 MHz, DCM-d_2) of **Re(L$_{\text{Me-nHo}}$)Py**.

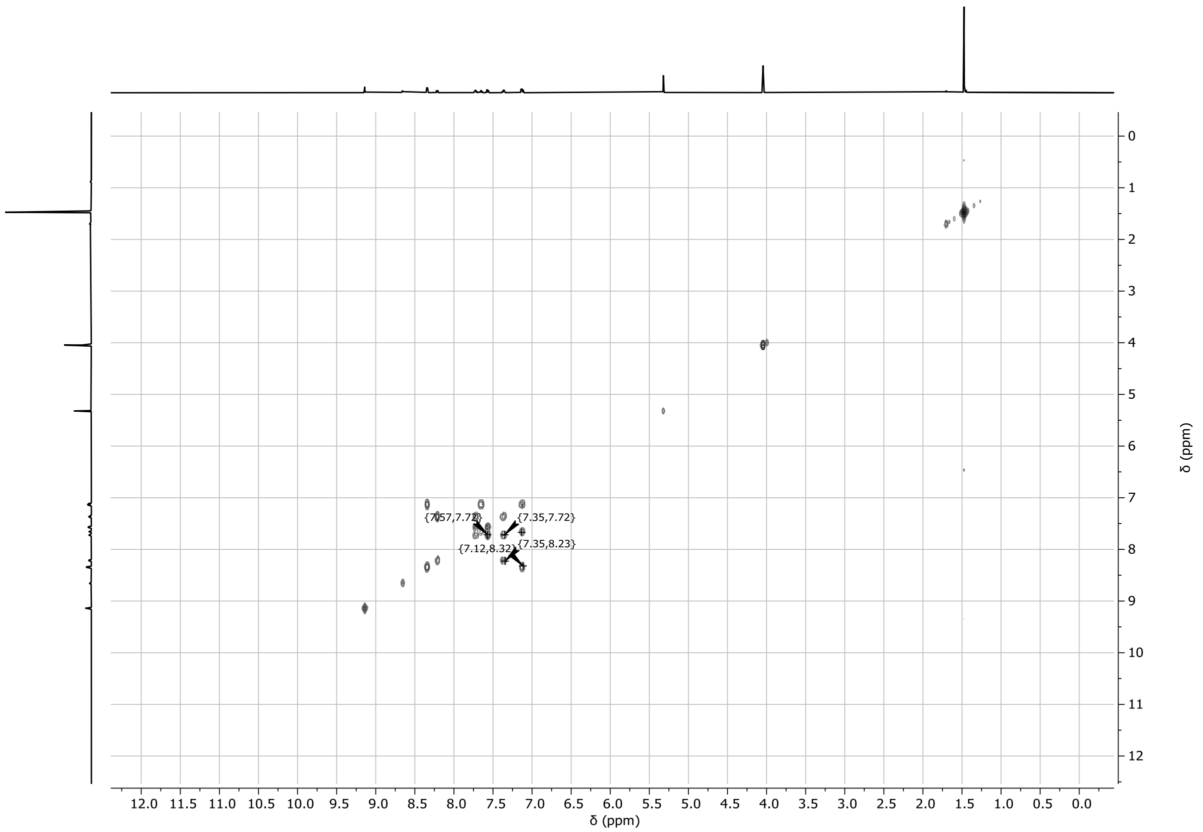

Figure S 50. HH-COSY-NMR spectrum (500 MHz, DCM-d_2) of **Re(L$_{\text{Me-nHo}}$)Py**.

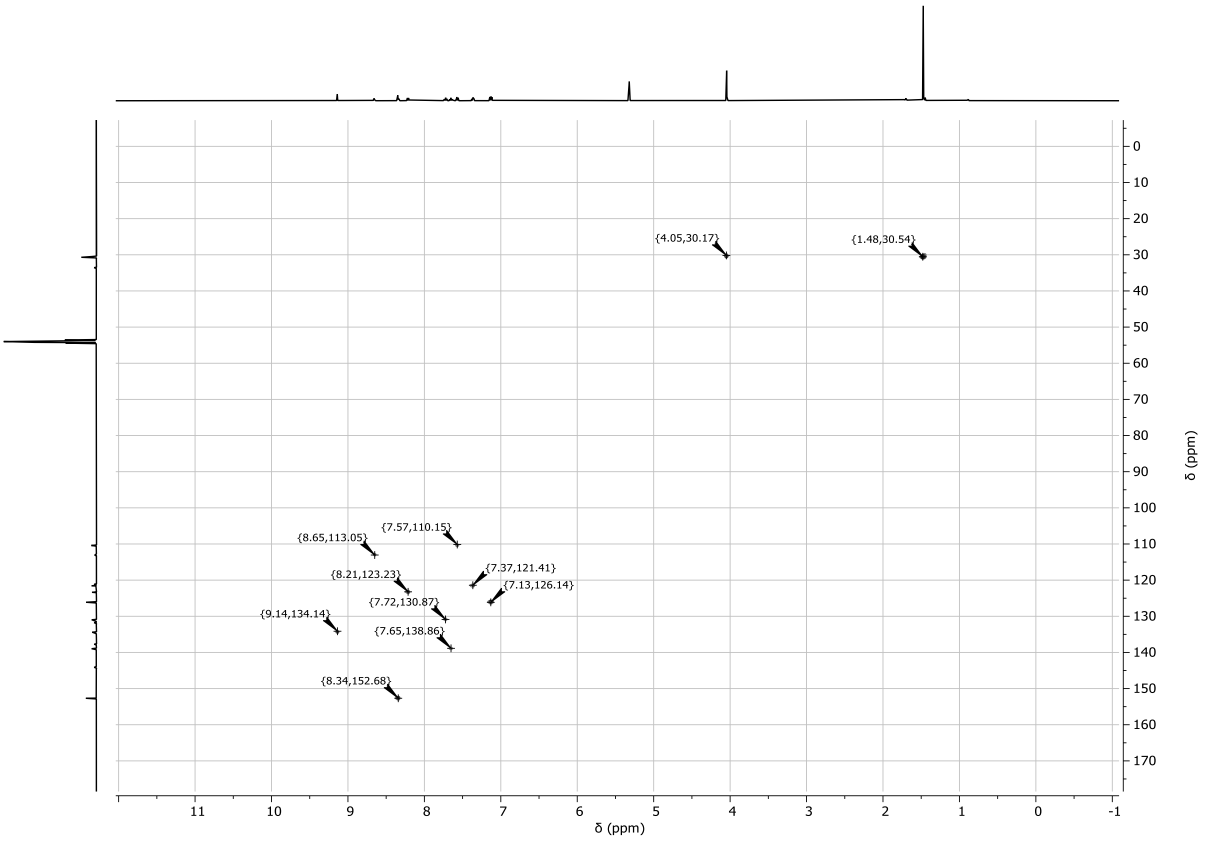

Figure S 51. HC-HSQC-NMR spectrum (126 MHz (^{13}C), 500 MHz (^{1}H), DCM-d_2) of **Re(L$_{\text{Me-nHo}}$)Py**.

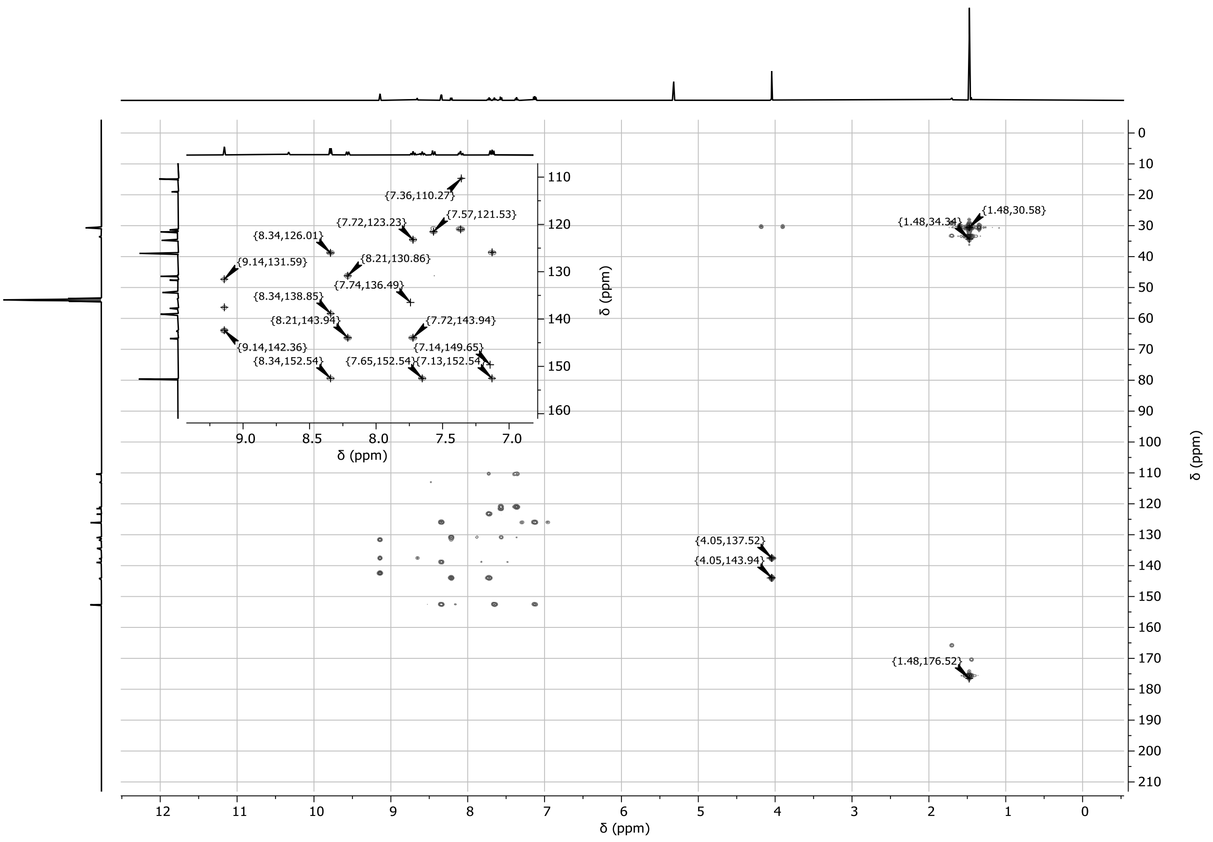

Figure S 52. HC-HMBC-NMR spectrum (126 MHz (^{13}C), 500 MHz (^{1}H), DCM-d_2) of **Re(L$_{\text{Me-nHo}}$)Py**.

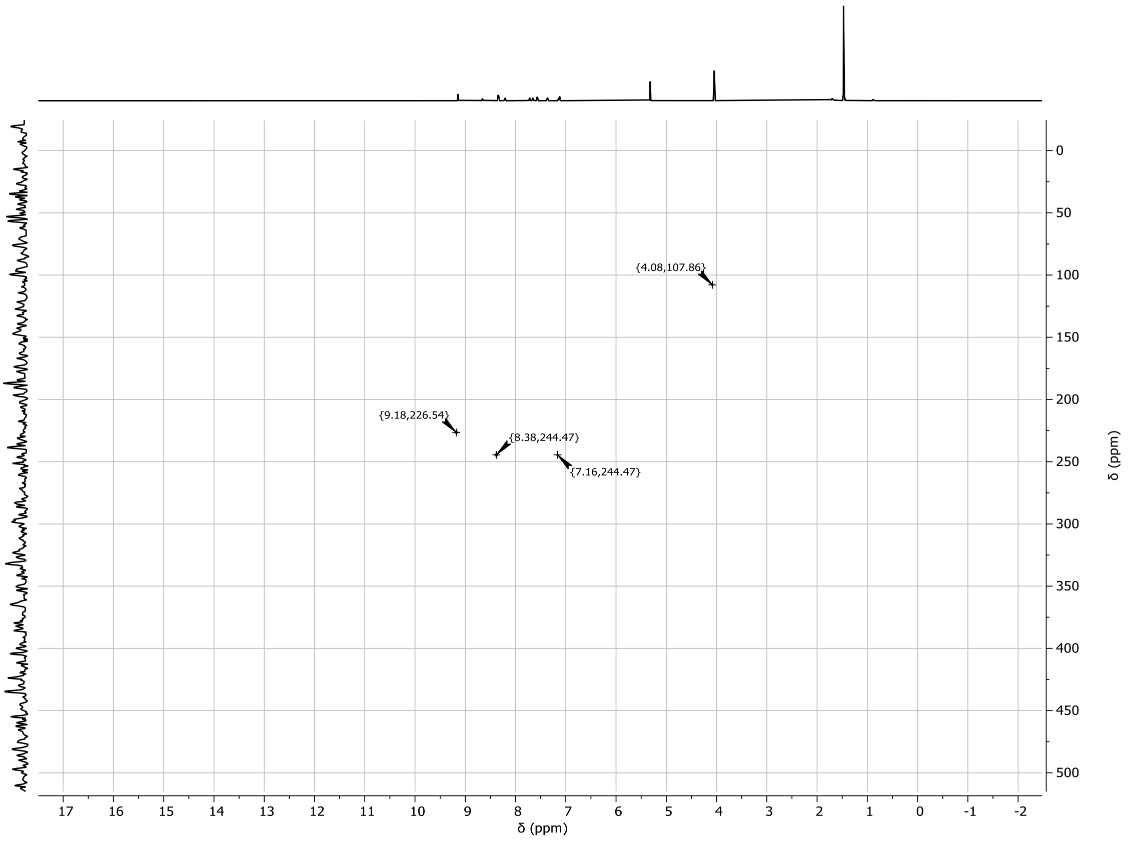

Figure S 53. HN-HMBC-NMR spectrum (51 MHz (^{15}N), 500 MHz (^{1}H), DCM-d_2) of **Re(L$_{\text{Me-nHo}}$)Py**.

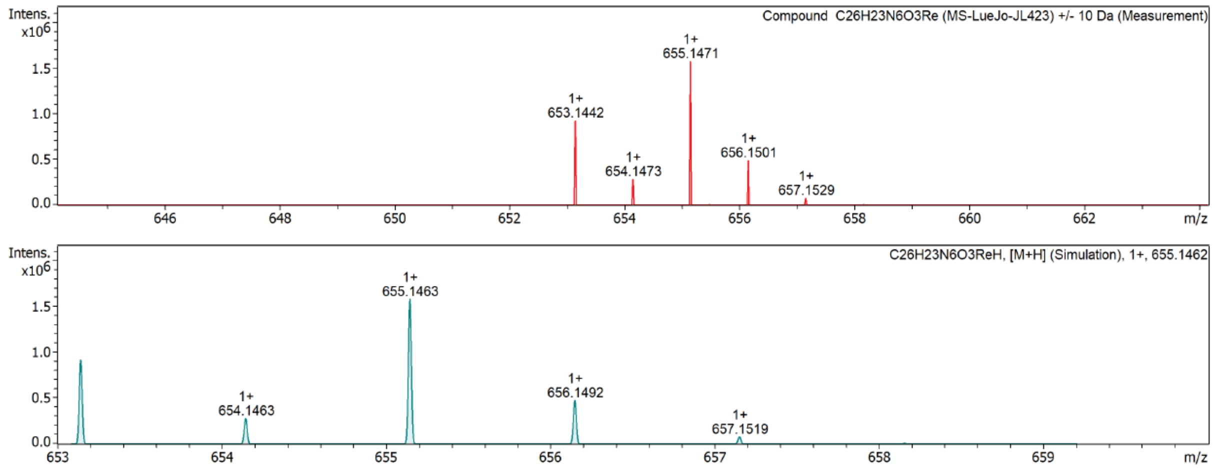

Figure S 54. Mass spectrum of **Re(L$_{\text{Me-nHo}}$)Py** (MeOH). Additional simulation of the [**Re(L$_{\text{Me-nHo}}$)Py**+H]$^+$ adduct.

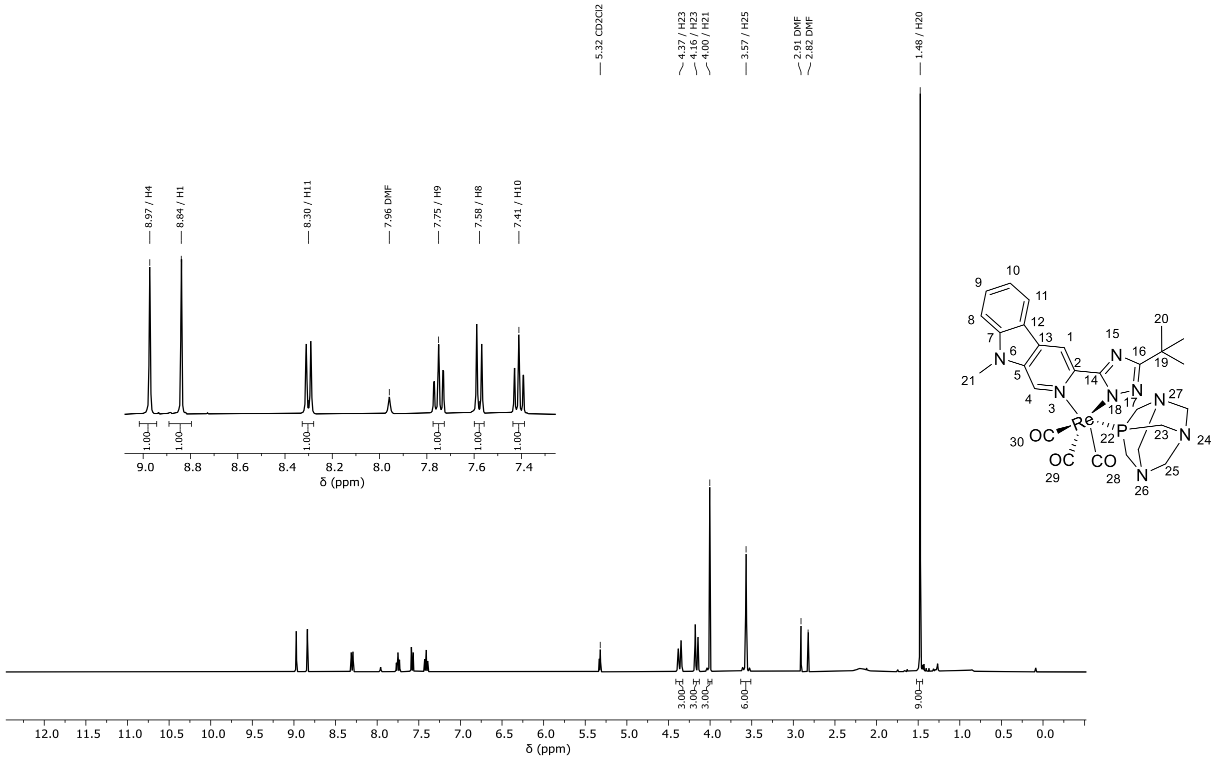

Figure S-55. ^{1}H-NMR spectrum (400 MHz, DCM-d_2) of **Re(L$_{Me-nHo}$)PTA**.

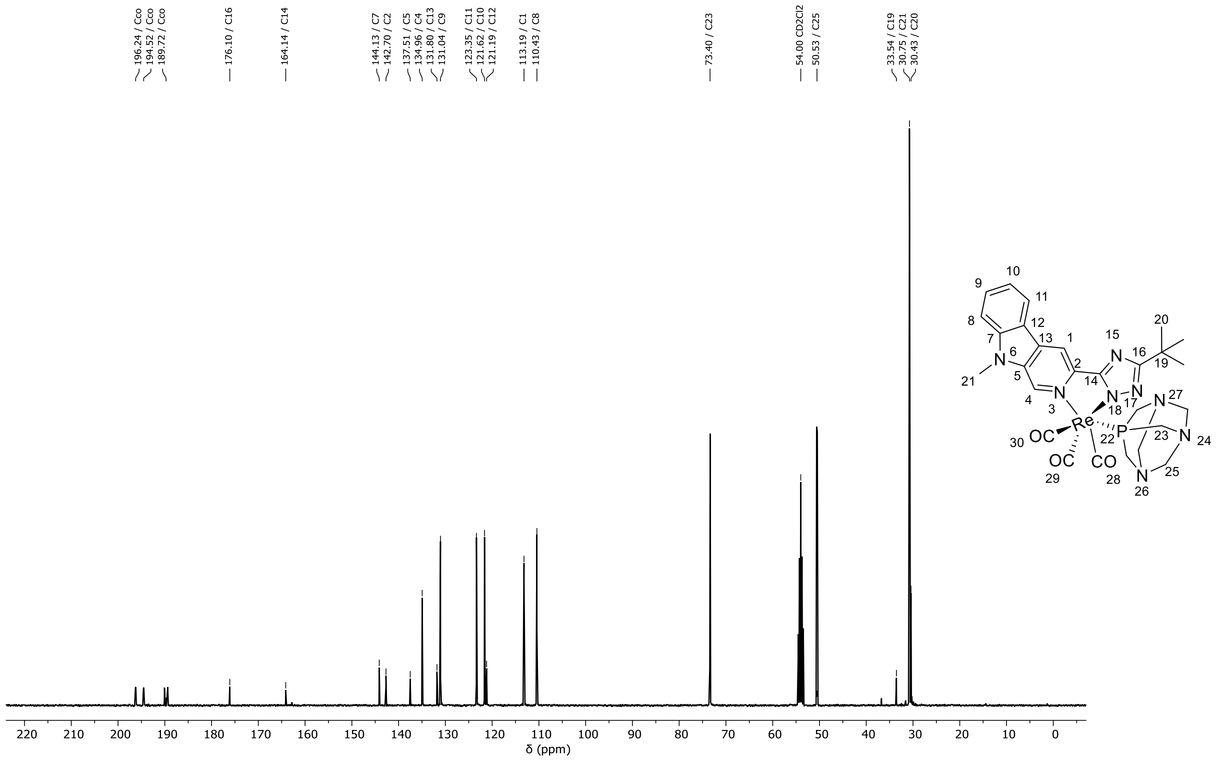

Figure S-56. ^{13}C{^{1}H}-NMR spectrum (101 MHz, DCM-d_2) of **Re(L$_{Me-nHo}$)PTA**.

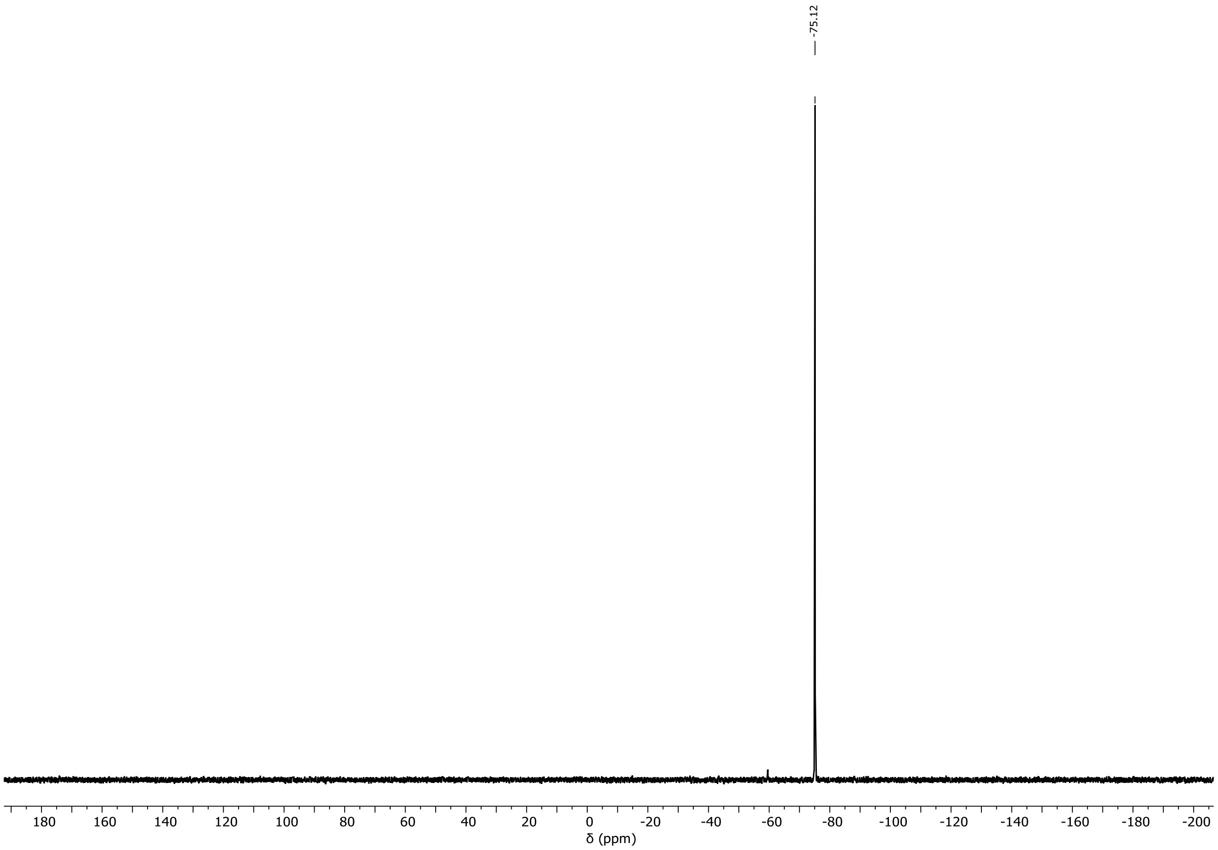

Figure S-57. $^{31}P\{^{1}H\}$-NMR spectrum (162 MHz, DCM-d_2) of $\mathbf{Re(L_{Me\text{-}nHo})PTA}$.

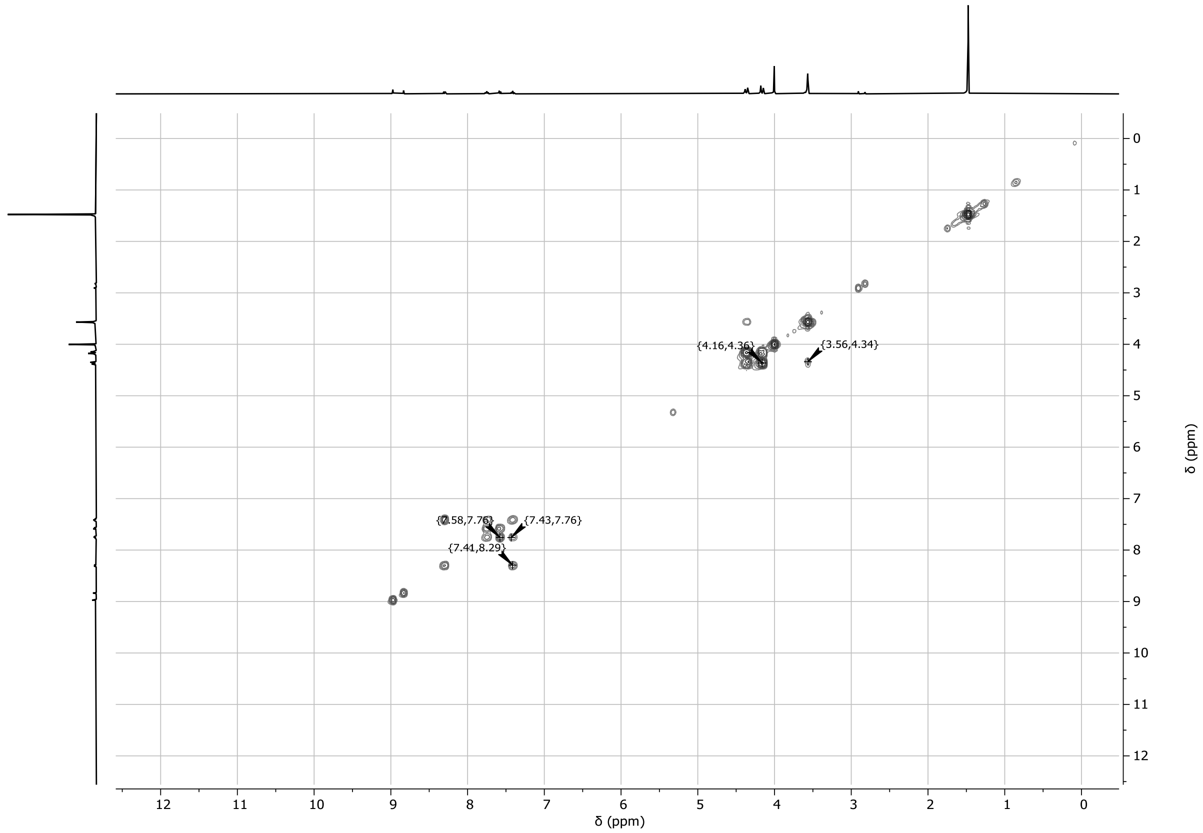

Figure S-58. HH-COSY-NMR spectrum (400 MHz, DCM-d_2) of $\mathbf{Re(L_{Me\text{-}nHo})PTA}$.

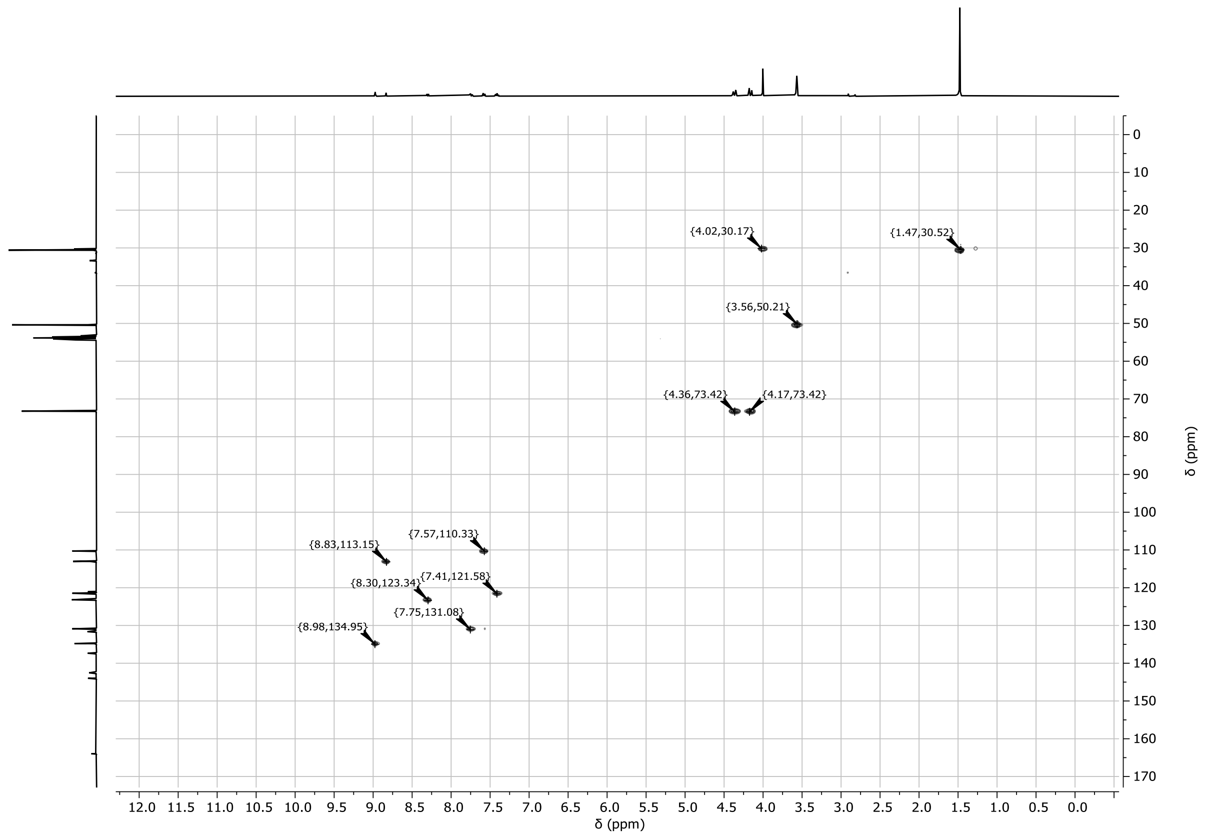

Figure S-59. HC-HSQC-NMR spectrum (101 MHz (^{13}C), 400 MHz (^{1}H), DCM-d_2) of **Re(L$_{\text{Me-nHo}}$)PTA**.

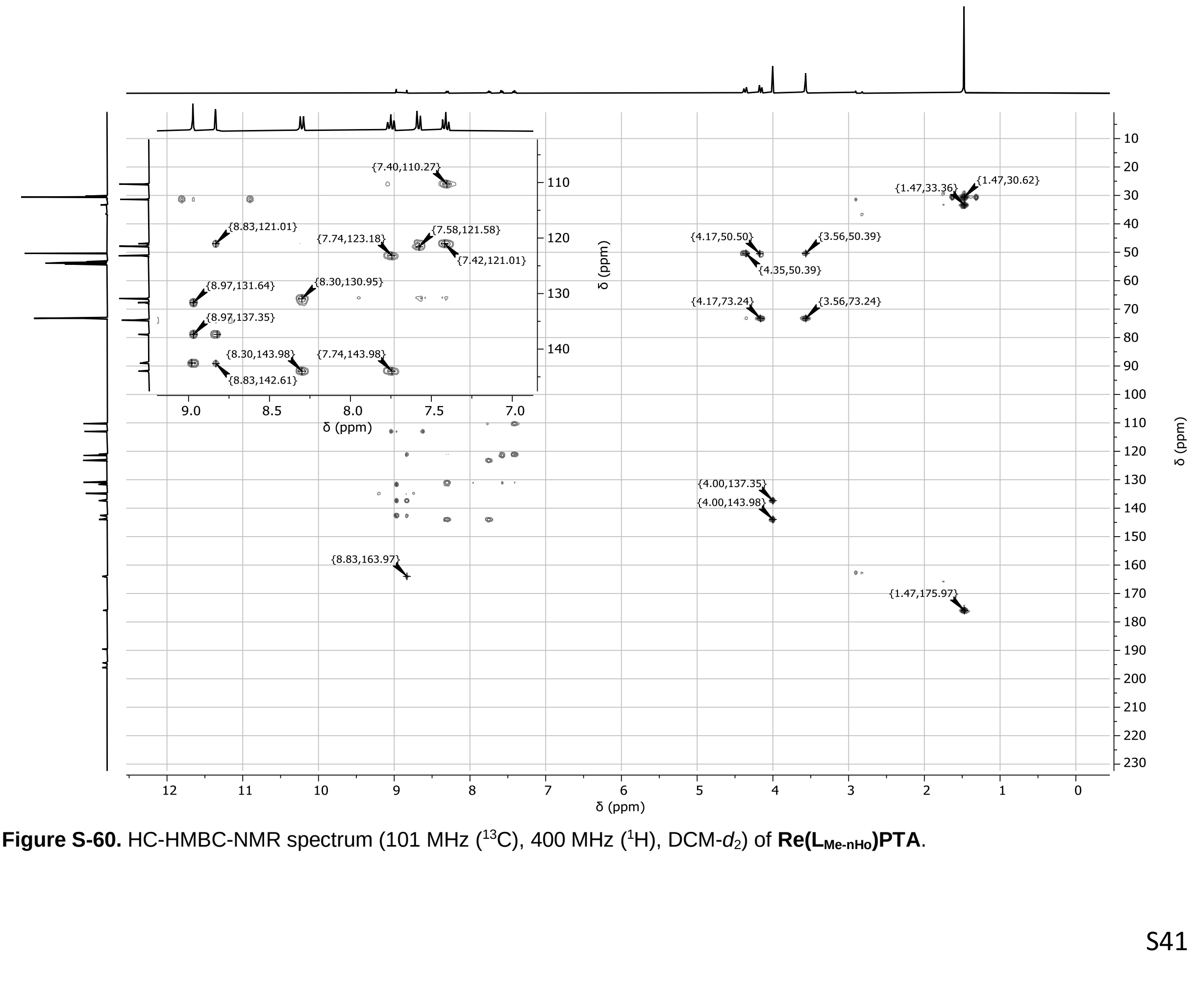

Figure S-60. HC-HMBC-NMR spectrum (101 MHz (^{13}C), 400 MHz (^{1}H), DCM-d_2) of **Re(L$_{\text{Me-nHo}}$)PTA**.

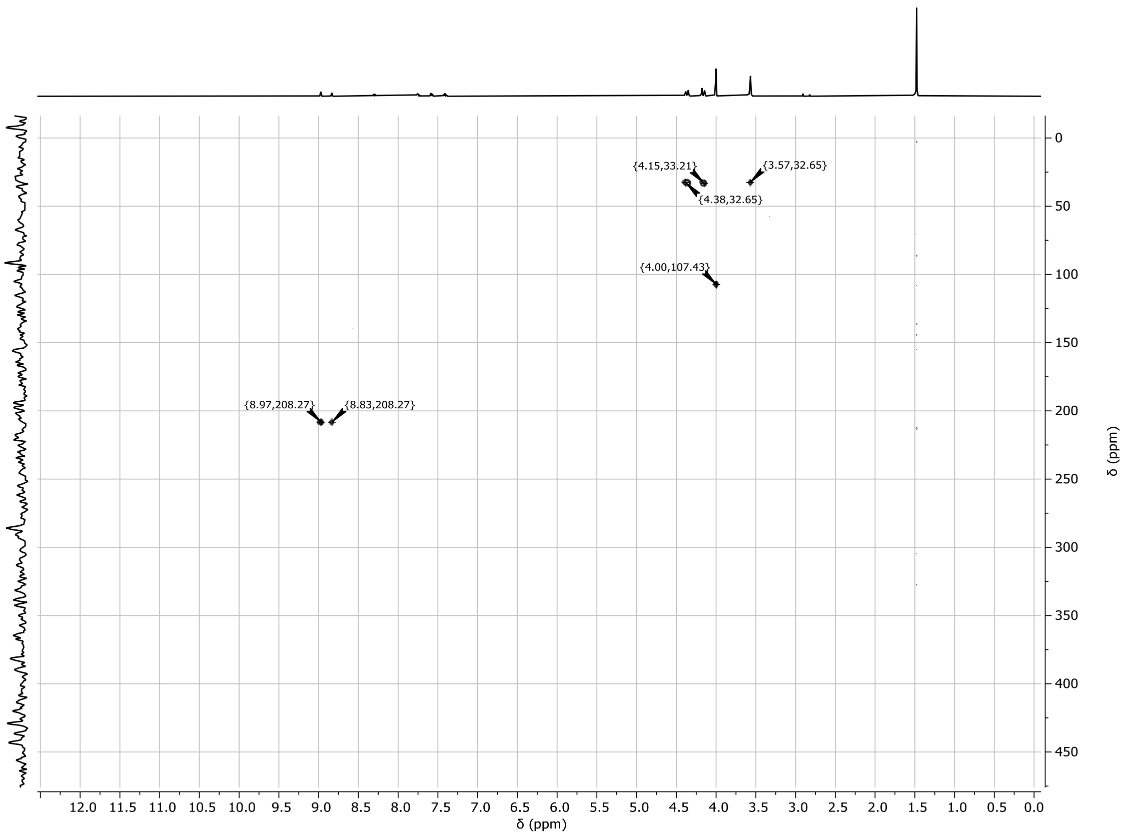

Figure S-61. HN-HMBC-NMR spectrum (41 MHz (^{15}N), 400 MHz (^{1}H), DCM-d_2) of **Re(L$_{Me\text{-}nHo}$)PTA**.

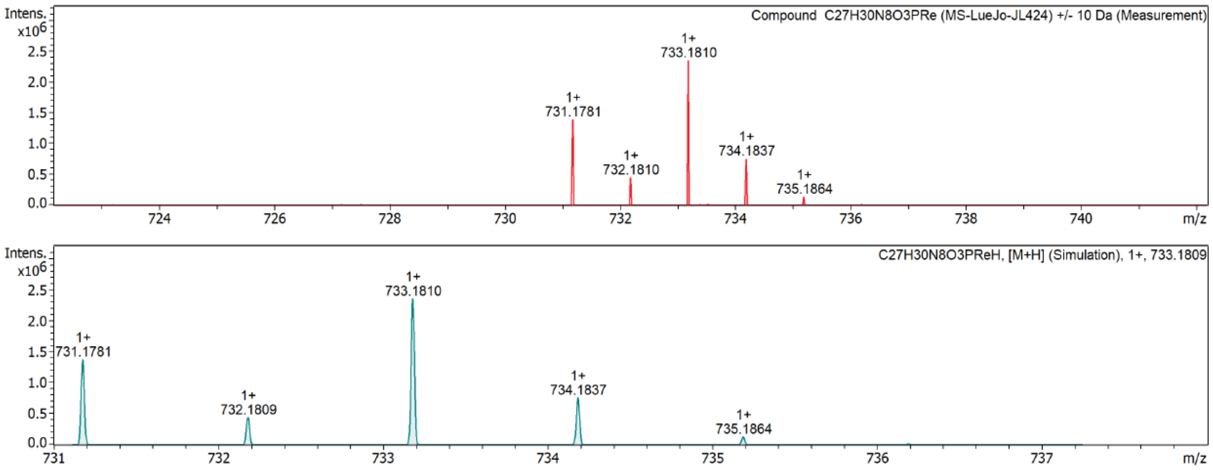

Figure S-62. Mass spectrum of **Re(L$_{Me\text{-}nHo}$)PTA** (MeOH). Additional simulation of the [**Re(L$_{Me\text{-}nHo}$)PTA**+H]$^{+}$ adduct.

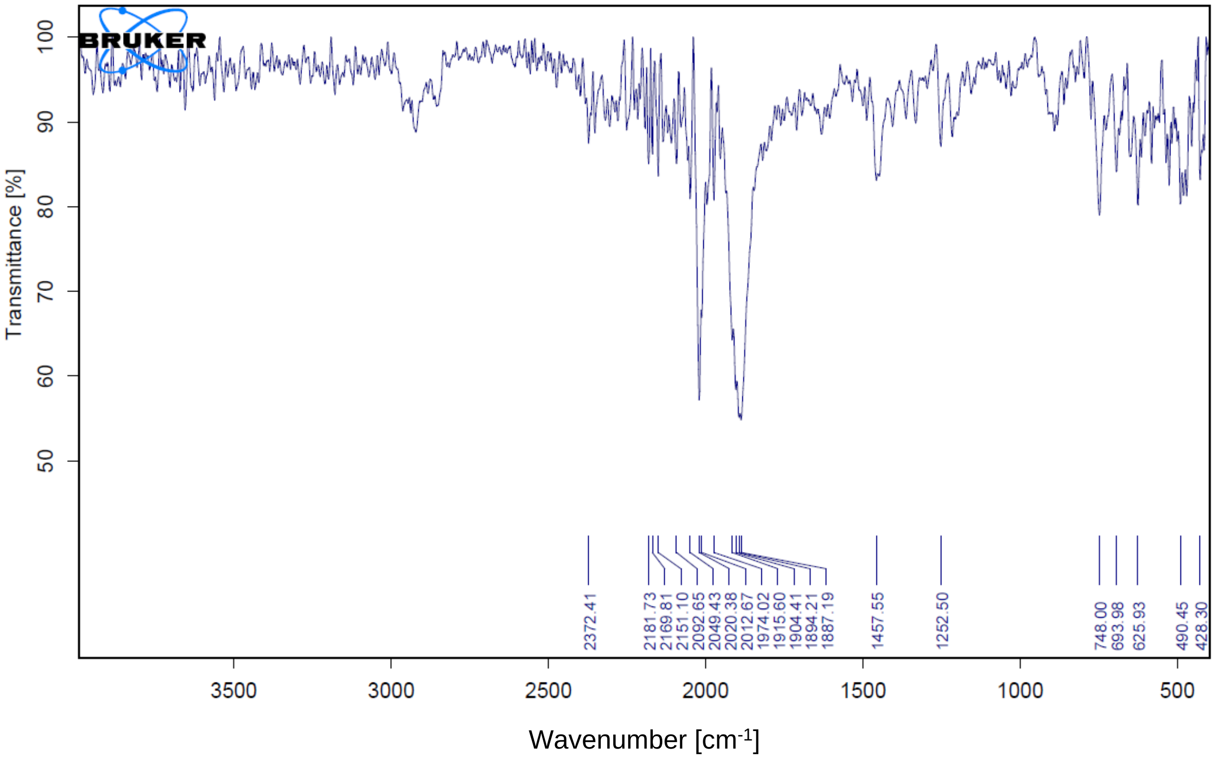

Figure S 63. IR spectrum of **Re(L_{nHo})Py**.

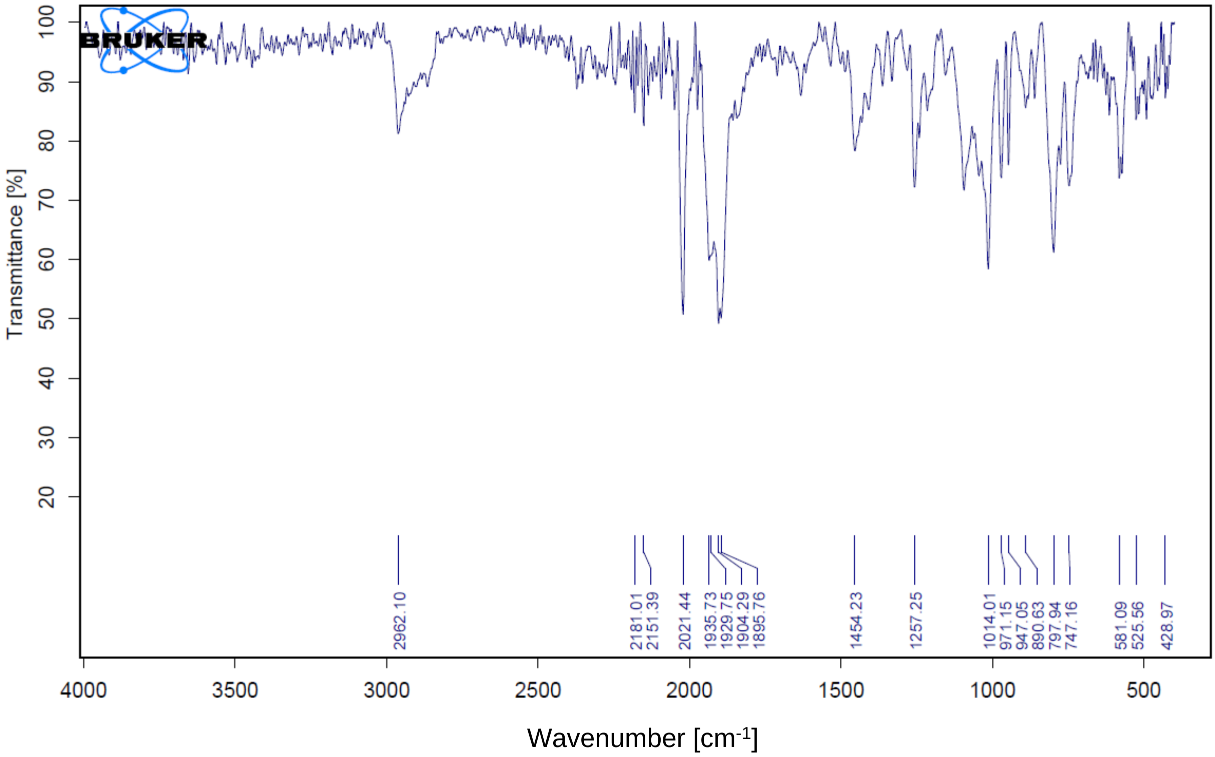

Figure S 64. IR spectrum of **Re(L_{nHo})PTA**.

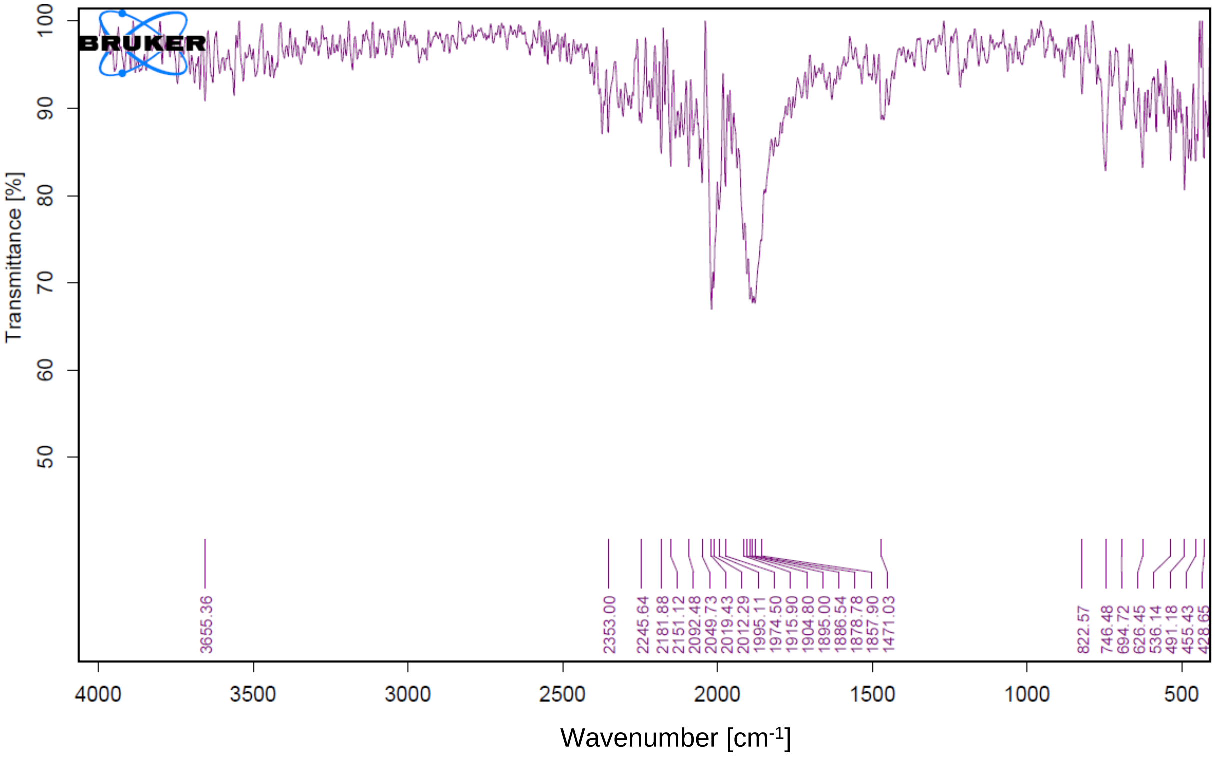

Figure S 65. IR spectrum of **Re(L_{Me-nHo})Py**.

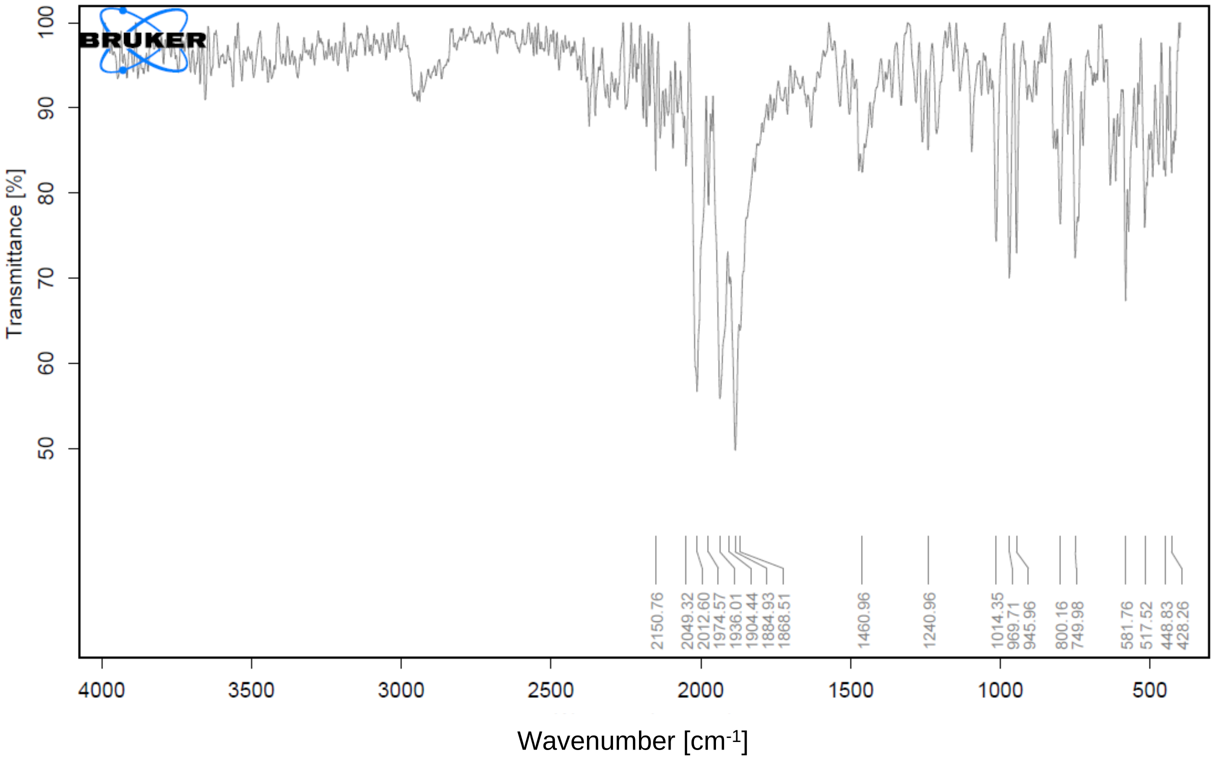

Figure S 66. IR spectrum of **Re(L_{Me-nHo})PTA**.

Section S4: Photophysical characterization of the complexes in solution

Table S-1: Complete set of photophysical data.

		λ_{abs} (nm) / ε (10^4 $M^{-1}cm^{-1}$)	λ_{exc} (nm)	λ_{em} (nm)	τ (µs)		Φ_L (%)		$k_{r\ (RT,\ Ar)}$ / 10^4 s^{-1}	$k_{nr\ (RT,\ Ar)}$ / 10^4 s^{-1}
					air	Ar	air	Ar		
	liquid solution<br>(298 K, ACN, 10^{-5} M)	273 / 3.33<br>297 / 3.48<br>376 / 0.52	337, 396	482, 503	τ_{av_amp} = 0.038 ± 0.002<br>[τ_1 = 0.074 ± 0.001 (49%)]<br>[τ_2 = 0.003 ± 0.001 (51%)]	τ_{av_amp} = 13.77 ± 0.09<br>[τ_1 = 15.30 ± 0.01 (89%)]<br>[τ_2 = 1.2 ± 0.1 (11%)]	< 2	14 ± 2	9 ± 1	56 ± 2
**Re(L_{nHo})Py**	liquid solution<br>(298 K, DCM, 10^{-5} M)	n.d.	313, 382	484	τ_{av_amp} = 0.484 ± 0.001<br>[τ_1 = 0.770 ± 0.004 (49%)]<br>[τ_2 = 0.210 ± 0.003 (51%)]	τ_{av_amp} = 10.85 ± 0.03<br>[τ_1 = 11.45 ± 0.03 (92%)]<br>[τ_2 = 4.2 ± 0.4 (8%)]	< 2	26 ± 3	2.3 ± 0.2	6.6 ± 0.2
	glassy matrix<br>(DCM/MeOH, 77 K, 10^{-7} M)	n.d.	310, 393	468, 500, 529	τ_{av_amp} = 83.6 ± 0.2<br>[τ_1 = 114 ± 3 (44%)]<br>[τ_2 = 60 ± 2 (56%)]		95 ± 5		1.13 ± 0.03	0.06 ± 0.02
	liquid solution<br>(298 K, ACN, 10^{-5} M)	274 / 3.67<br>375 / 0.55	310	475, 499	τ_{av_amp} = 0.063 ± 0.001<br>[τ_1 = 0.073 ± 0.001 (86%)]<br>[τ_2 = 0.005 ± 0.001 (14%)]	2.084 ± 0.003	< 2	< 2	< 2	47 ± 1
**Re(L_{nHo})PTA**	liquid solution<br>(298 K, DCM, 10^{-5} M)	n.d.	307, 383	475, 495	τ_{av_amp} = 0.357 ± 0.001<br>[τ_1 = 0.554 ± 0.004 (49%)]<br>[τ_2 = 0.167 ± 0.006 (51%)]	τ_{av_amp} = 3.46 ± 0.01<br>[τ_1 = 6.4± 0.3 (13%)]<br>[τ_2 = 3.04 ± 0.05 (87%)]	< 2	6 ± 2	1.7 ± 0.6	27.5 ± 0.6
	glassy matrix<br>(DCM/MeOH, 77 K, 10^{-7} M)	n.d.	309, 392	464, 492, 528	τ_{av_amp} = 141.7 ± 0.2<br>[τ_1 = 195 ± 2 (44%)]<br>[τ_2 = 99 ± 1 (56%)]		95 ± 5		0.67 ± 0.02	0.04 ± 0.01
	liquid solution<br>(298 K, ACN, 10^{-5} M)	275 / 4.02<br>388 / 0.44	319, 407	492, 520	τ_{av_amp} = 0.059 ± 0.001<br>[τ_1 = 0.093 ± 0.001 (58%)]<br>[τ_2 = 0.011 ± 0.001 (42%)]	54.03 ± 0.06	< 2	26 ± 3	0.48 ± 0.04	1.37 ± 0.04
**Re($L_{Me\text{-}nHo}$)Py**	liquid solution<br>(298 K, DCM, 10^{-5} M)	n.d.	313, 397	495, 517	τ_{av_amp} = 0.612 ± 0.004<br>[τ_1 = 0.776 ± 0.003 (72%)]<br>[τ_2 = 0.20 ± 0.01 (28%)]	τ_{av_amp} = 38.4 ± 0.2<br>[τ_1 = 42.08 ± 0.04 (91%)]<br>[τ_2 = 2.8 ± 0.3 (9%)]	< 2	43 ± 4	1.03 ± 0.05	1.36 ± 0.05
	glassy matrix<br>(DCM/MeOH, 77 K, 10^{-7} M)	n.d.	310, 403	473, 504, 538	τ_{av_amp} = 111.4 ± 0.2<br>[τ_1 = 1498 ± 2 (41%)]<br>[τ_2 = 86 ± 2 (59%)]		95 ± 5		0.85 ± 0.01	0.04 ± 0.02
	liquid solution<br>(298 K, ACN, 10^{-5} M)	278 / 3.98<br>388 / 0.53	409	487, 513	τ_{av_amp} = 0.037 ± 0.001<br>[τ_1 = 0.088 ± 0.001 (80%)]<br>[τ_2 = 0.005 ± 0.001 (20%)]	12.458 ± 0.005	< 2	10 ± 2	0.8 ± 0.2	7.2 ± 0.2
**Re($L_{Me\text{-}nHo}$)PTA**	liquid solution<br>(298 K, DCM, 10^{-5} M)	n.d.	325, 411	487, 514	τ_{av_amp} = 0.284 ± 0.001<br>[τ_1 = 0.086 ± 0.001 (39%)]<br>[τ_2 = 0.005 ± 0.001 (61%)]	τ_{av_amp} = 28.12 ± 0.05<br>[τ_1 = 31.8 ± 0.3 (71%)]<br>[τ_2 = 19 ± 1 (29%)]	< 2	22 ± 2	0.77 ± 0.07	2.76 ± 0.07
	glassy matrix<br>(DCM/MeOH, 77 K, 10^{-7} M)	n.d.	314, 402	472, 504, 539	τ_{av_amp} = 171.6 ± 0.3<br>[τ_1 = 213 ± 2 (54%)]<br>[τ_2 = 124 ± 3 (46%)]		95 ± 5		0.55 ± 0.01	0.03 ± 0.01

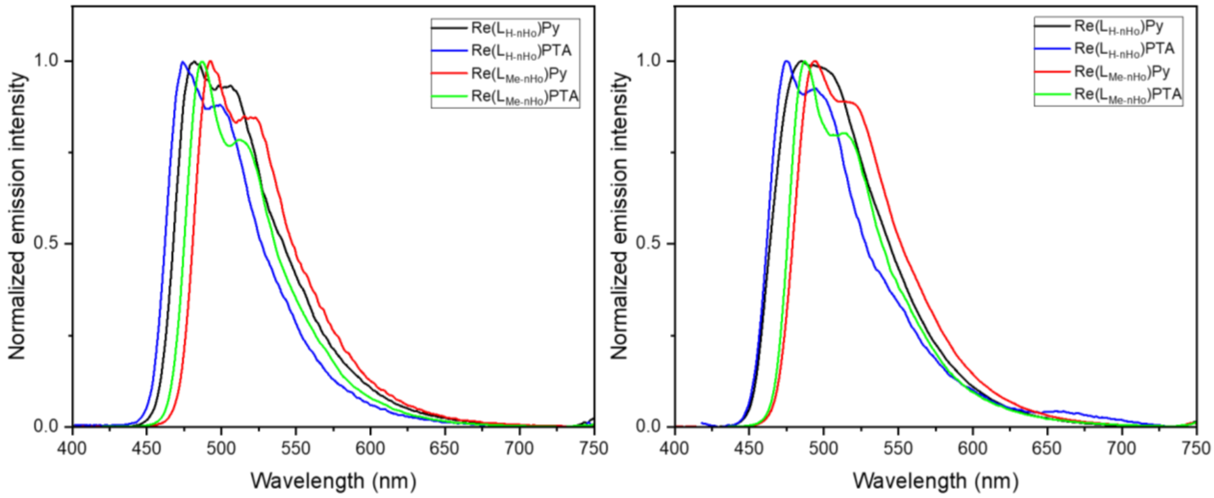

Figure S-67. Photoluminescence emission spectra of **Re($_{nHo}$)Py**, **Re(L$_{nHo}$)PTA**, **Re(L$_{Me\text{-}nHo}$)Py** and **Re(L$_{Me\text{-}nHo}$)PTA** at 298 K in liquid acetonitrile (c = 10^{-5} M, left) and **Re(L$_{nHo}$)Py**, **Re(L$_{nHo}$)PTA**, **Re(L$_{Me\text{-}nHo}$)Py** and **Re(L$_{Me\text{-}nHo}$)PTA** at 298 K in liquid DCM (c = 10^{-5} M, right). Spectra normalized to the highest intensity (λ_{exc} = 350 nm).

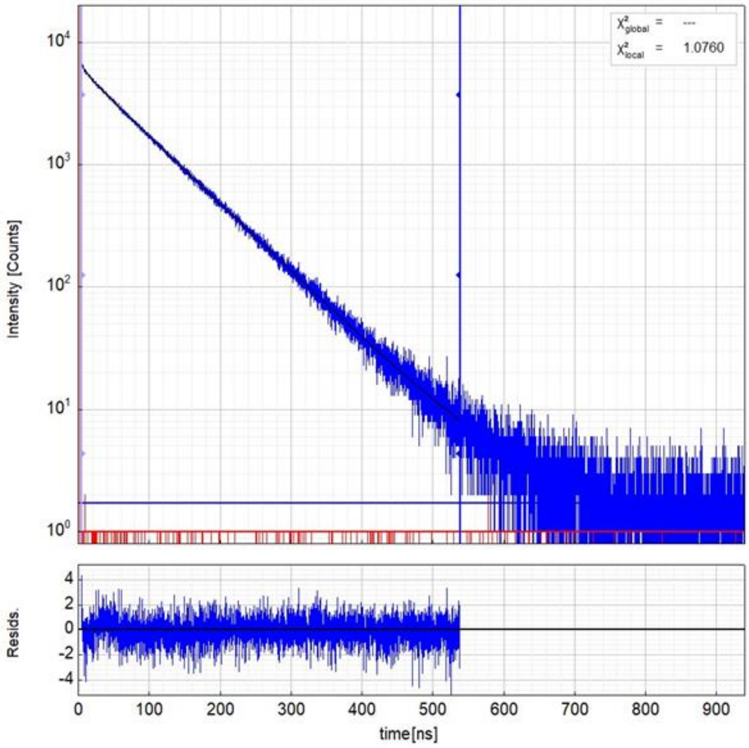

Parameter	Value	Δ	δ
A_1[kCnts/Chnl]	0.98	±0.12	11%
τ_1[ns]	10.5	±2.2	21%
I_1[kCnts]	79.6	±9.2	12%
A_{Rel1}[%]	14.1	±1.4	9.5%
I_{Rel1}[%]	2.2	±0.3	12%
A_2[kCnts/Chnl]	5.959	±0.031	0.5%
τ_2[ns]	78.52	±0.19	0.2%
I_2[kCnts]	3 655	±12	0.3%
A_{Rel2}[%]	86.0	±1.4	1.6%
I_{Rel2}[%]	97.9	±0.3	0.3%
$Bkgr_{Dec}$[kCnts]	0.0017	---	---
$Bkgr_{IRF}$[Cnts/Chnl]	1.0000	---	---
$Shift_{IRF}$[ps]	1.0000	---	---
τ_{AvInt}[ns]	77.07	±0.11	0.1%
τ_{AvAmp}[ns]	69.0	±1.3	1.9%

Figure S-68. Left: Raw (experimental) time-resolved photoluminescence decay of **Re(L$_{nHo}$)Py** in liquid acetonitrile at 298 K (air-equilibrated, blue), including the residuals (λ_{exc} = 376.7 nm, λ_{em} = 482 nm) and the instrumental response function (IRF, red). Right: Fitting parameters including pre-exponential factors and confidence limits.

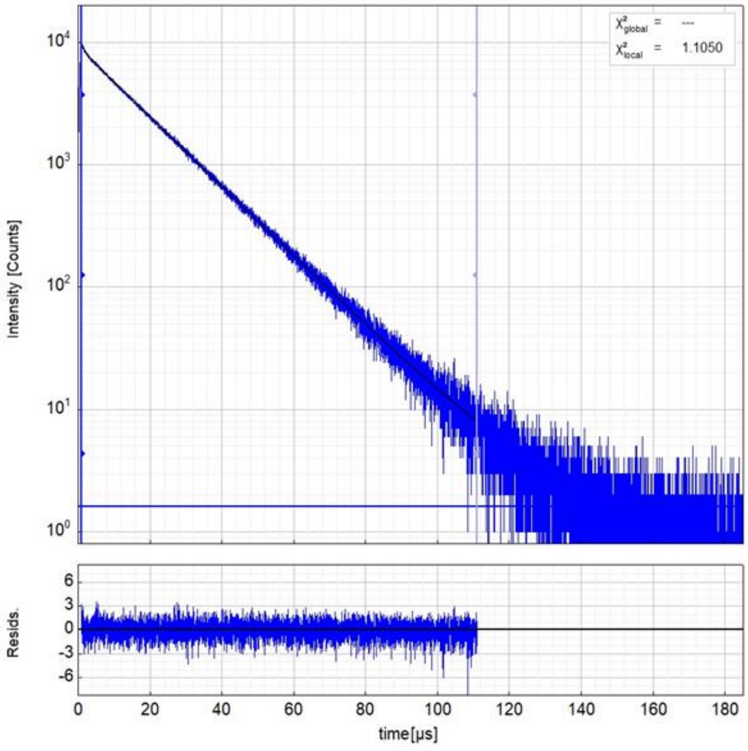

Parameter	Value	Δ	δ
A_1[kCnts/Chnl]	8.506	±0.014	0.2%
τ_1[ns]	15 296	±14	0.1%
I_1[kCnts]	8 131.0	±7.9	0.1%
A_{Rel1}[%]	89.2	±0.6	0.7%
I_{Rel1}[%]	99.1	±0.1	0.1%
A_2[kCnts/Chnl]	1.033	±0.064	6.2%
τ_2[ns]	1 170	±120	9.8%
I_2[kCnts]	75.0	±4.8	6.4%
A_{Rel2}[%]	10.9	±0.6	5.4%
I_{Rel2}[%]	1.0	±0.1	6.4%
$Bkgr_{Dec}$[kCnts]	0.0016	---	---
τ_{AvInt}[ns]	15 167	±11	0.1%
τ_{AvAmp}[ns]	13 766	±96	0.7%

Figure S-69. Left: Raw (experimental) time-resolved photoluminescence decay of **Re(L_{nHo})Py** in liquid acetonitrile at 298 K (Ar-purged), including the residuals (λ_{exc} = 376.7 nm, λ_{em} = 482 nm). Right: Fitting parameters including pre-exponential factors and confidence limits.

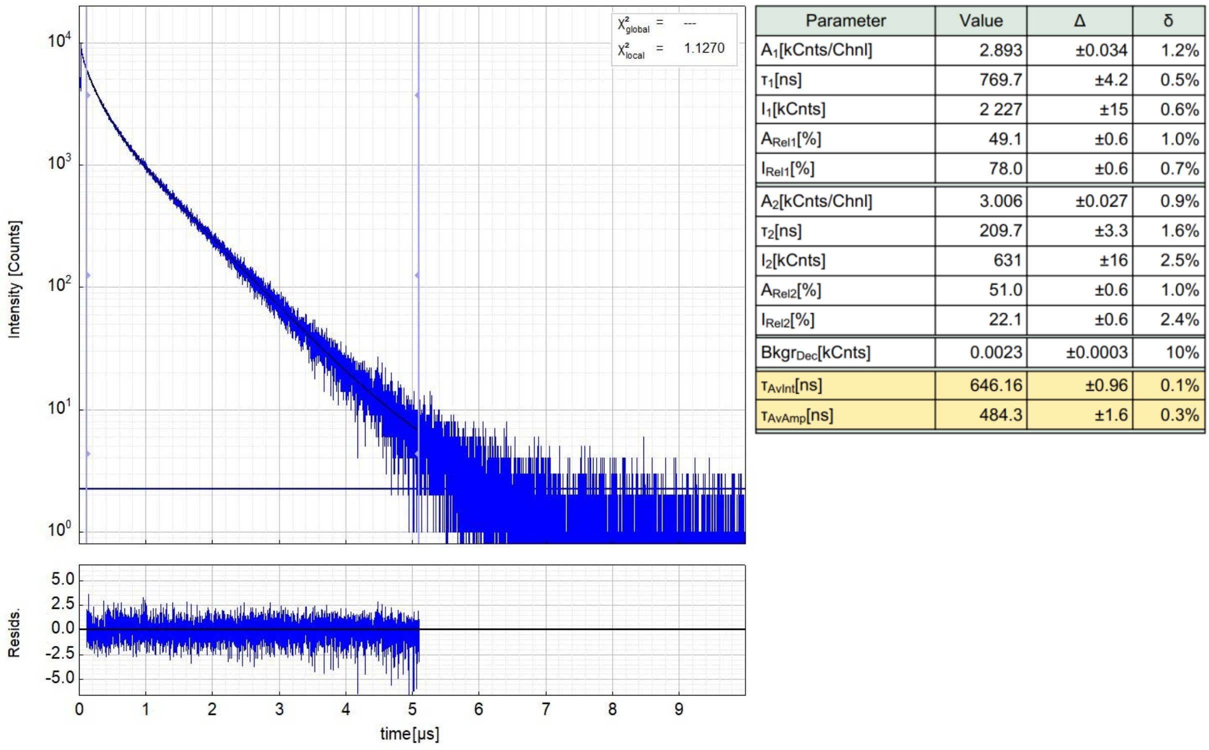

Parameter	Value	Δ	δ
A_1[kCnts/Chnl]	2.893	±0.034	1.2%
τ_1[ns]	769.7	±4.2	0.5%
I_1[kCnts]	2 227	±15	0.6%
A_{Rel1}[%]	49.1	±0.6	1.0%
I_{Rel1}[%]	78.0	±0.6	0.7%
A_2[kCnts/Chnl]	3.006	±0.027	0.9%
τ_2[ns]	209.7	±3.3	1.6%
I_2[kCnts]	631	±16	2.5%
A_{Rel2}[%]	51.0	±0.6	1.0%
I_{Rel2}[%]	22.1	±0.6	2.4%
$Bkgr_{Dec}$[kCnts]	0.0023	±0.0003	10%
τ_{AvInt}[ns]	646.16	±0.96	0.1%
τ_{AvAmp}[ns]	484.3	±1.6	0.3%

Figure S-70. Left: Raw (experimental) time-resolved photoluminescence decay of **Re(L_{nHo})Py** in liquid DCM at 298 K (air-equilibrated), including the residuals (λ_{exc} = 376.7 nm, λ_{em} = 498 nm). Right: Fitting parameters including pre-exponential factors and confidence limits.

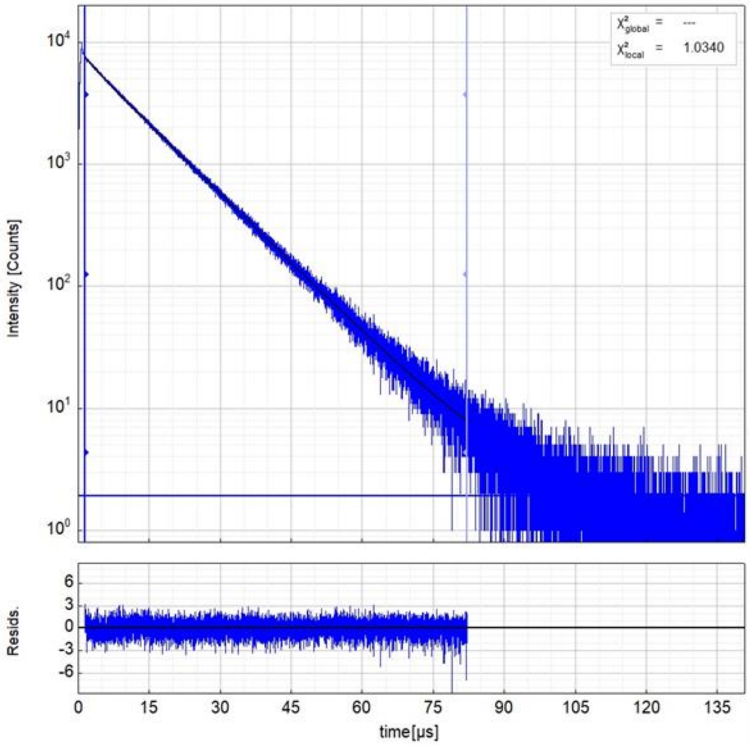

Parameter	Value	Δ	δ
A_1[kCnts/Chnl]	6.902	±0.048	0.7%
τ_1[ns]	11 445	±26	0.2%
I_1[kCnts]	9 874	±48	0.5%
A_{Rel1}[%]	91.9	±0.6	0.5%
I_{Rel1}[%]	96.9	±0.5	0.5%
A_2[kCnts/Chnl]	0.611	±0.038	6.1%
τ_2[ns]	4 160	±390	9.2%
I_2[kCnts]	318	±47	15%
A_{Rel2}[%]	8.2	±0.6	6.2%
I_{Rel2}[%]	3.2	±0.5	15%
$Bkgr_{Dec}$[kCnts]	0.0019	---	---
τ_{AvInt}[ns]	11 218.0	±6.1	0.1%
τ_{AvAmp}[ns]	10 853	±25	0.2%

Figure S-71. Left: Raw (experimental) time-resolved photoluminescence decay of **Re(L_{nHo})Py** in liquid DCM at 298 K (Ar-purged), including the residuals (λ_{exc} = 376.7 nm, λ_{em} = 500 nm). Right: Fitting parameters including pre-exponential factors and confidence limits.

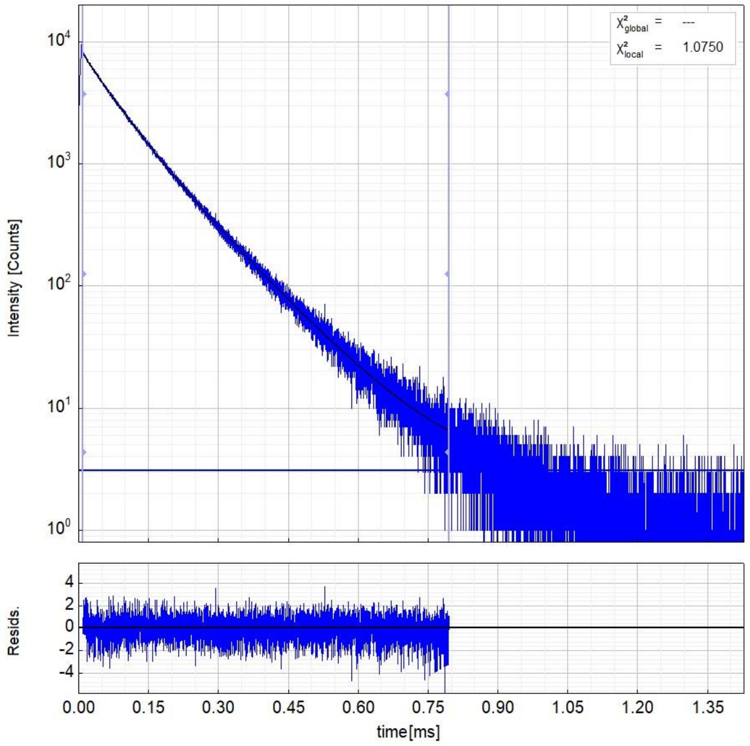

Parameter	Value	Δ	δ
A_1[kCnts/Chnl]	3.49	±0.31	8.8%
τ_1[ns]	113 500	±2 900	2.5%
I_1[kCnts]	3 090	±200	6.4%
A_{Rel1}[%]	44.2	±3.9	8.8%
I_{Rel1}[%]	60.0	±4.0	6.6%
A_2[kCnts/Chnl]	4.41	±0.31	6.9%
τ_2[ns]	60 000	±1 800	2.8%
I_2[kCnts]	2 070	±210	9.9%
A_{Rel2}[%]	55.9	±3.9	6.9%
I_{Rel2}[%]	40.1	±4.0	9.8%
$Bkgr_{Dec}$[kCnts]	0.0031	±0.0007	21%
τ_{AvInt}[ns]	92 010	±270	0.3%
τ_{AvAmp}[ns]	83 550	±150	0.2%

Figure S-72. Left: Raw (experimental) time-resolved photoluminescence decay of **Re(L_{nHo})Py** in a frozen glassy matrix of DCM/MeOH (V:V = 1:1) at 77 K, including the residuals (λ_{exc} = 376.7 nm, λ_{em} = 464 nm). Right: Fitting parameters including pre-exponential factors and confidence limits.

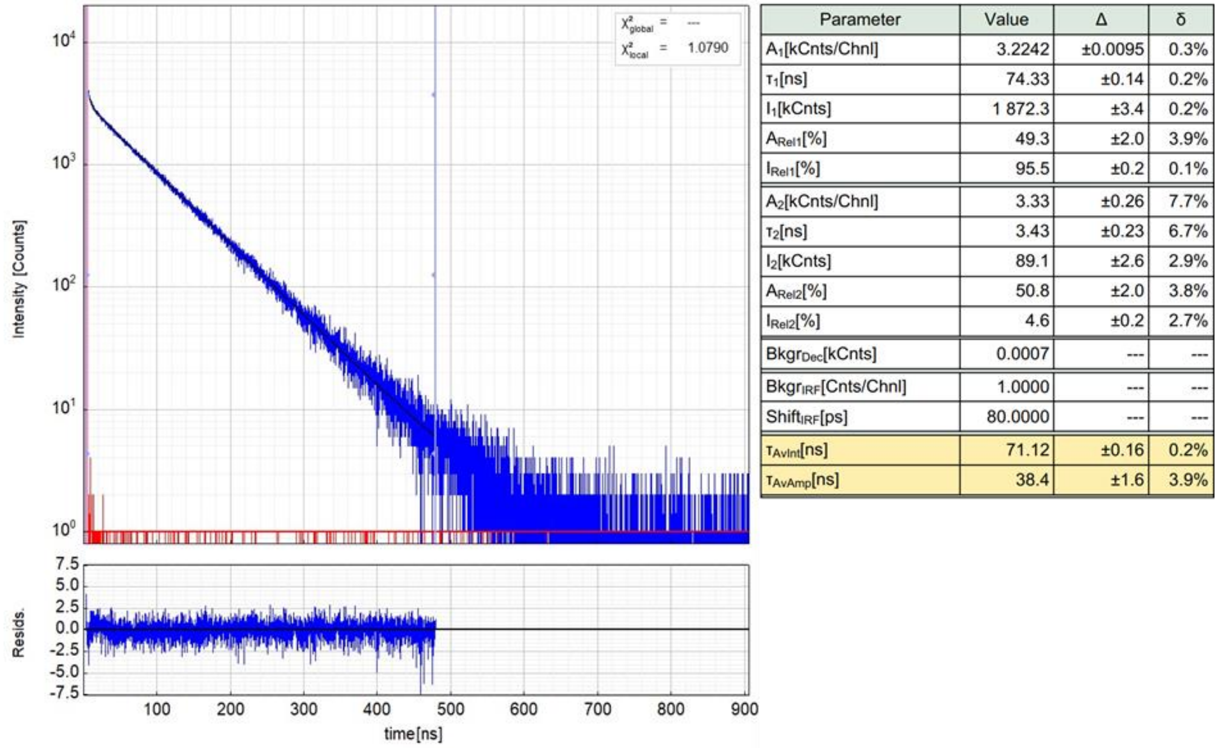

Parameter	Value	Δ	δ
A_1[kCnts/Chnl]	3.2242	±0.0095	0.3%
τ_1[ns]	74.33	±0.14	0.2%
I_1[kCnts]	1 872.3	±3.4	0.2%
A_{Rel1}[%]	49.3	±2.0	3.9%
I_{Rel1}[%]	95.5	±0.2	0.1%
A_2[kCnts/Chnl]	3.33	±0.26	7.7%
τ_2[ns]	3.43	±0.23	6.7%
I_2[kCnts]	89.1	±2.6	2.9%
A_{Rel2}[%]	50.8	±2.0	3.8%
I_{Rel2}[%]	4.6	±0.2	2.7%
$Bkgr_{Dec}$[kCnts]	0.0007	---	---
$Bkgr_{IRF}$[Cnts/Chnl]	1.0000	---	---
$Shift_{IRF}$[ps]	80.0000	---	---
τ_{AvInt}[ns]	71.12	±0.16	0.2%
τ_{AvAmp}[ns]	38.4	±1.6	3.9%

Figure S-73. Raw (experimental) time-resolved photoluminescence decay of **Re(L$_{nHo}$)PTA** in liquid acetonitrile at 298 K (air-equilibrated, blue), including the residuals (λ_{exc} = 376.7 nm, λ_{em} = 495 nm) and the instrument response function (IRF, red). Right: Fitting parameters including pre-exponential factors and confidence limits.

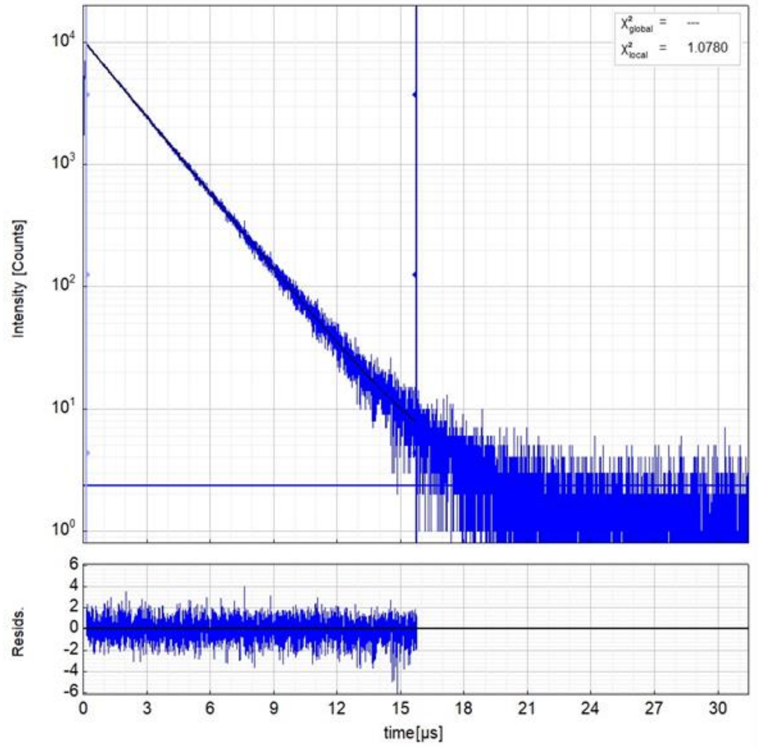

Parameter	Value	Δ	δ
A_1[kCnts/Chnl]	9.636	±0.017	0.2%
τ_1[ns]	2 083.9	±2.8	0.1%
I_1[kCnts]	5 019.9	±6.1	0.1%
A_{Rel1}[%]	100.0	---	---
I_{Rel1}[%]	100.0	---	---
$Bkgr_{Dec}$[kCnts]	0.0024	±0.0004	15%
τ_{AvInt}[ns]	2 083.9	±2.8	0.1%
τ_{AvAmp}[ns]	2 083.9	±2.8	0.1%

Figure S-74. Left: Raw (experimental) time-resolved photoluminescence decay of **Re(L$_{nHo}$)PTA** in liquid acetonitrile at 298 K (Ar-purged), including the residuals (λ_{exc} = 376.7 nm, λ_{em} = 495 nm). Right: Fitting parameters including pre-exponential factors and confidence limits.

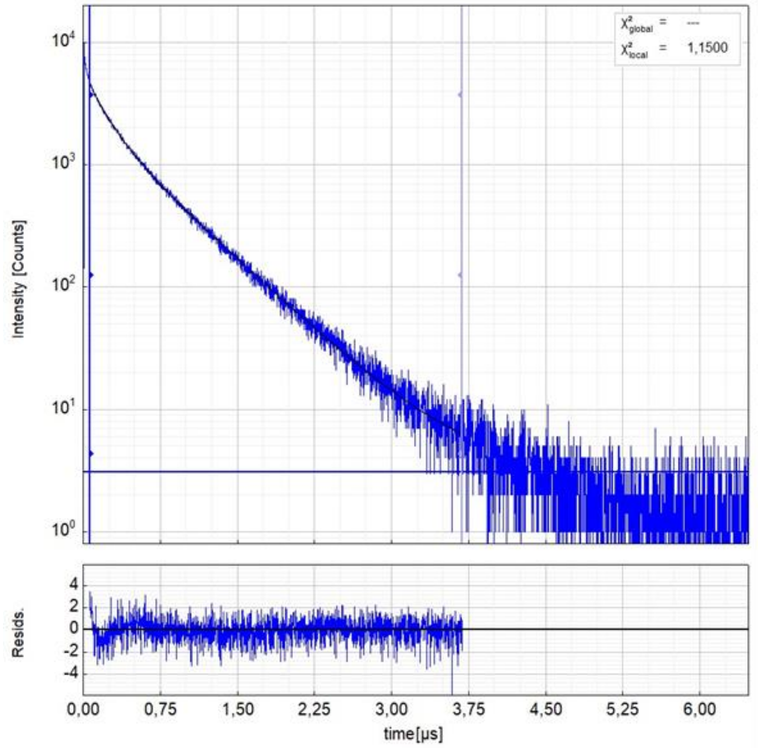

Parameter	Value	Δ	δ
A_1[kCnts/Chnl]	2,253	±0,048	2,1%
τ_1[ns]	553,6	±4,4	0,8%
I_1[kCnts]	623,5	±8,7	1,4%
A_{Rel1}[%]	49,1	±0,9	1,7%
I_{Rel1}[%]	76,2	±1,1	1,4%
A_2[kCnts/Chnl]	2,338	±0,043	1,8%
τ_2[ns]	167,2	±6,5	3,8%
I_2[kCnts]	195,5	±8,7	4,4%
A_{Rel2}[%]	51,0	±0,9	1,7%
I_{Rel2}[%]	23,9	±1,1	4,4%
$Bkgr_{Dec}$[kCnts]	0,0031	---	---
τ_{AvInt}[ns]	461,4	±1,4	0,3%
τ_{AvAmp}[ns]	356,8	±3,4	0,9%

Figure S-75. Left: Raw (experimental) time-resolved photoluminescence decay of **Re(L$_{nHo}$)PTA** in liquid DCM at 298 K (air-equilibrated), including the residuals (λ_{exc} = 376.7 nm, λ_{em} = 489 nm). Right: Fitting parameters including pre-exponential factors and confidence limits.

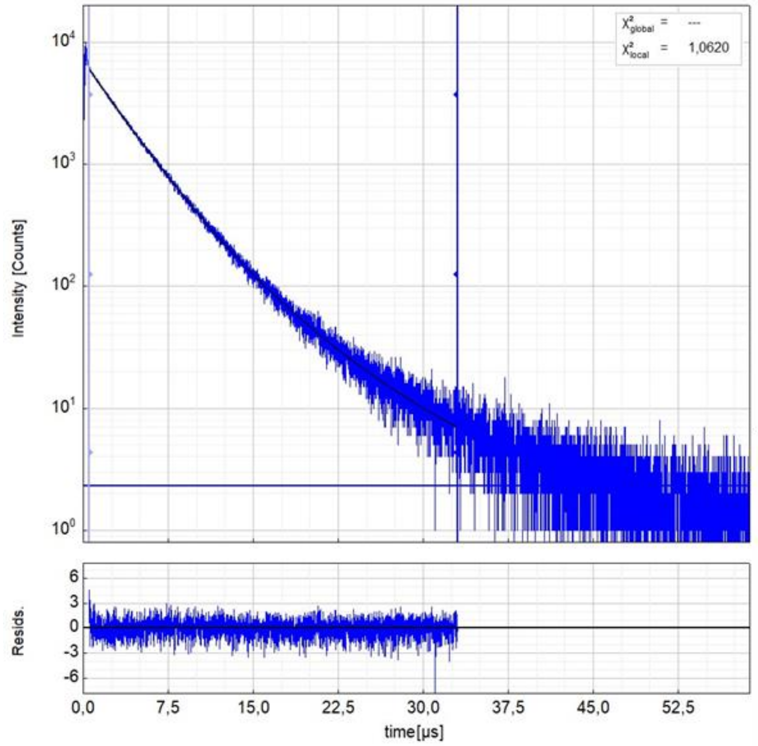

Parameter	Value	Δ	δ
A_1[kCnts/Chnl]	0,76	±0,11	14%
τ_1[ns]	6 370	±250	3,8%
I_1[kCnts]	605	±64	11%
A_{Rel1}[%]	12,8	±1,8	14%
I_{Rel1}[%]	23,4	±2,5	11%
A_2[kCnts/Chnl]	5,221	±0,094	1,8%
τ_2[ns]	3 036	±47	1,5%
I_2[kCnts]	1 981	±63	3,2%
A_{Rel2}[%]	87,3	±1,8	2,0%
I_{Rel2}[%]	76,7	±2,5	3,2%
$Bkgr_{Dec}$[kCnts]	0,0023	---	---
τ_{AvInt}[ns]	3 814	±12	0,3%
τ_{AvAmp}[ns]	3 459	±13	0,4%

Figure S-76. Left: Raw (experimental) time-resolved photoluminescence decay of **Re(L$_{nHo}$)PTA** in liquid DCM at 298 K (Ar-purged), including the residuals (λ_{exc} = 376.7 nm, λ_{em} = 470 nm). Right: Fitting parameters including pre-exponential factors and confidence limits.

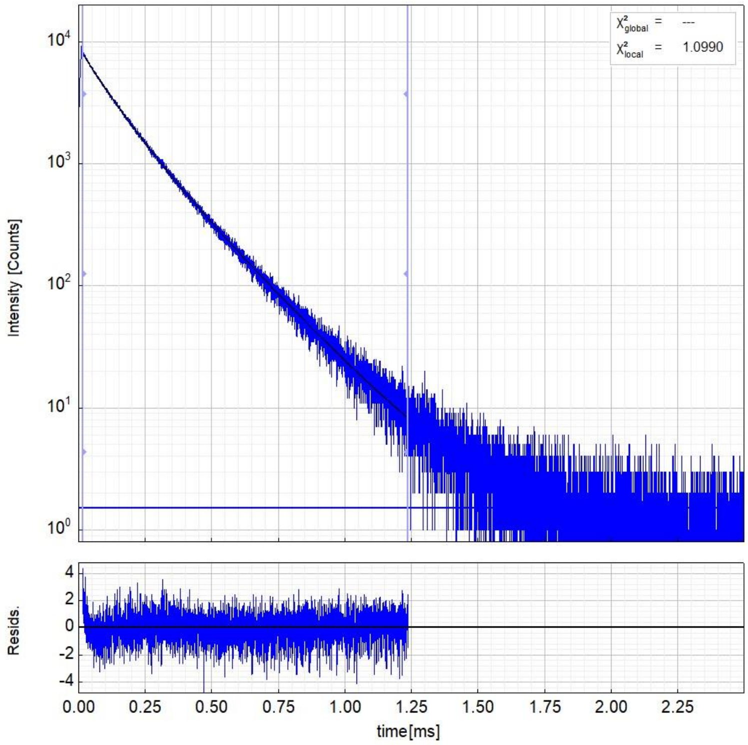

Parameter	Value	Δ	δ
A_1[kCnts/Chnl]	3.49	±0.11	3.0%
τ_1[ns]	195 300	±1 600	0.8%
I_1[kCnts]	2 660	±60	2.2%
A_{Rel1}[%]	44.3	±1.3	2.9%
I_{Rel1}[%]	61.1	±1.4	2.3%
A_2[kCnts/Chnl]	4.386	±0.098	2.2%
τ_2[ns]	99 200	±1 400	1.4%
I_2[kCnts]	1 699	±61	3.6%
A_{Rel2}[%]	55.8	±1.3	2.3%
I_{Rel2}[%]	39.0	±1.4	3.5%
$Bkgr_{Dec}$[kCnts]	0.0015	---	---
τ_{AvInt}[ns]	157 810	±180	0.1%
τ_{AvAmp}[ns]	141 730	±230	0.2%

Figure S-77. Left: Raw (experimental) time-resolved photoluminescence decay of **Re(L$_{nHo}$)PTA** in a frozen glassy matrix of DCM/MeOH (V:V = 1:1) at 77 K, including the residuals (λ_{exc} = 376.7 nm, λ_{em} = 464 nm). Right: Fitting parameters including pre-exponential factors and confidence limits.

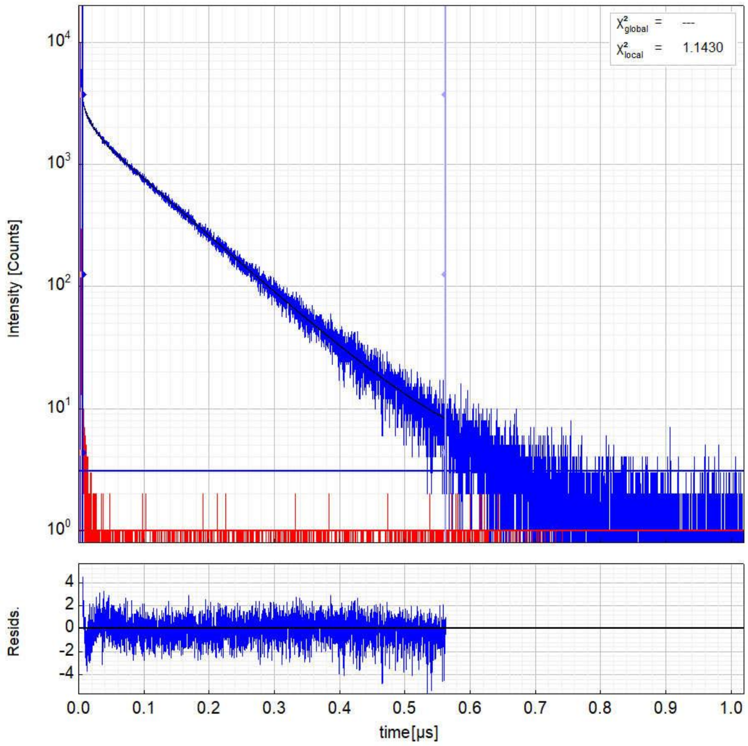

Parameter	Value	Δ	δ
A_1[kCnts/Chnl]	2.186	±0.016	0.7%
τ_1[ns]	92.59	±0.42	0.5%
I_1[kCnts]	1 580.7	±6.0	0.4%
A_{Rel1}[%]	58.3	±1.0	1.6%
I_{Rel1}[%]	92.0	±0.3	0.2%
A_2[kCnts/Chnl]	1.568	±0.069	4.4%
τ_2[ns]	11.37	±0.71	6.2%
I_2[kCnts]	139.2	±3.5	2.5%
A_{Rel2}[%]	41.8	±1.0	2.3%
I_{Rel2}[%]	8.1	±0.3	2.6%
$Bkgr_{Dec}$[kCnts]	0.0031	±0.0003	7.9%
$Bkgr_{IRF}$[Cnts/Chnl]	1.0000	---	---
$Shift_{IRF}$[ps]	-500.0000	---	---
τ_{AvInt}[ns]	86.02	±0.30	0.3%
τ_{AvAmp}[ns]	58.7	±1.2	2.0%

Figure S-78. Left: Raw (experimental) time-resolved photoluminescence decay of **Re(L$_{Me-nHo}$)Py** in liquid acetonitrile at 298 K (air-equilibrated, blue), including the residuals (λ_{exc} = 376.7 nm, λ_{em} = 497 nm) and the instrument response function (IRF, red). Right: Fitting parameters including pre-exponential factors and confidence limits.

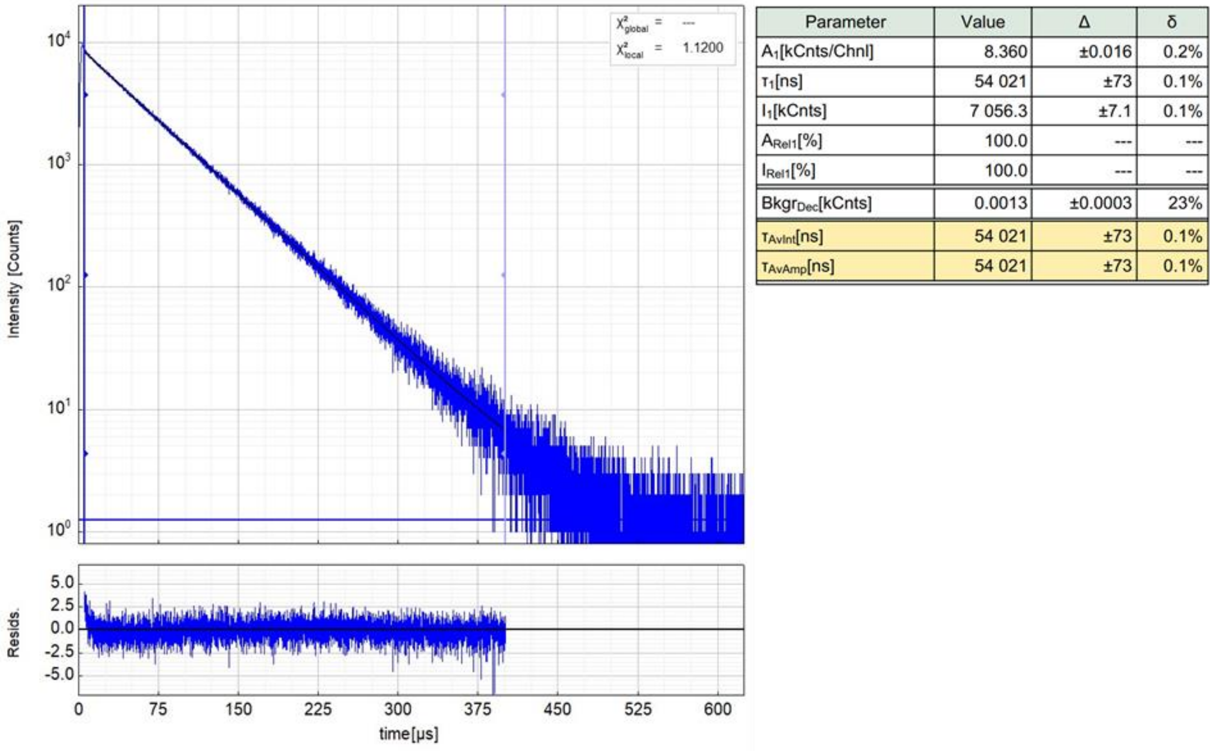

Parameter	Value	Δ	δ
A_1[kCnts/Chnl]	8.360	±0.016	0.2%
$τ_1$[ns]	54 021	±73	0.1%
I_1[kCnts]	7 056.3	±7.1	0.1%
A_{Rel1}[%]	100.0	---	---
I_{Rel1}[%]	100.0	---	---
$Bkgr_{Dec}$[kCnts]	0.0013	±0.0003	23%
$τ_{AvInt}$[ns]	54 021	±73	0.1%
$τ_{AvAmp}$[ns]	54 021	±73	0.1%

Figure S-79. Left: Raw (experimental) time-resolved photoluminescence decay of **Re(L$_{Me-nHo}$)Py** in liquid acetonitrile at 298 K (Ar-purged), including the residuals (λ_{exc} = 376.7 nm, λ_{em} = 482 nm). Right: Fitting parameters including pre-exponential factors and confidence limits.

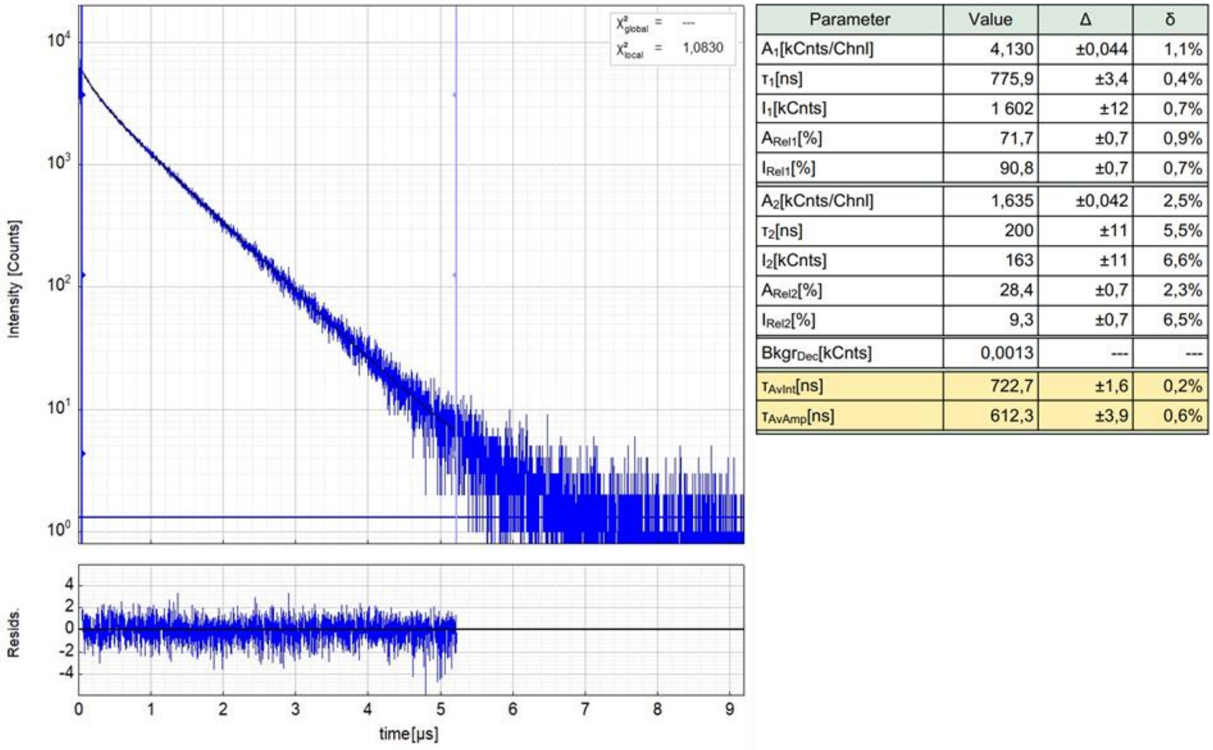

Parameter	Value	Δ	δ
A_1[kCnts/Chnl]	4,130	±0,044	1,1%
$τ_1$[ns]	775,9	±3,4	0,4%
I_1[kCnts]	1 602	±12	0,7%
A_{Rel1}[%]	71,7	±0,7	0,9%
I_{Rel1}[%]	90,8	±0,7	0,7%
A_2[kCnts/Chnl]	1,635	±0,042	2,5%
$τ_2$[ns]	200	±11	5,5%
I_2[kCnts]	163	±11	6,6%
A_{Rel2}[%]	28,4	±0,7	2,3%
I_{Rel2}[%]	9,3	±0,7	6,5%
$Bkgr_{Dec}$[kCnts]	0,0013	---	---
$τ_{AvInt}$[ns]	722,7	±1,6	0,2%
$τ_{AvAmp}$[ns]	612,3	±3,9	0,6%

Figure S-80. Left: Raw (experimental) time-resolved photoluminescence decay of **Re(L$_{Me-nHo}$)Py** in liquid DCM at 298 K (air-equilibrated), including the residuals (λ_{exc} = 376.7 nm, λ_{em} = 490 nm). Right: Fitting parameters including pre-exponential factors and confidence limits.

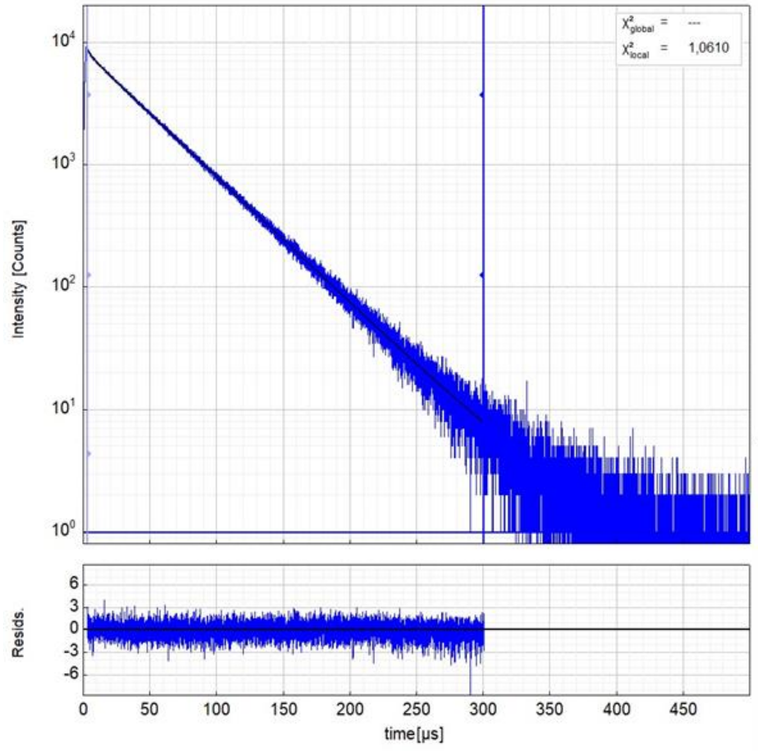

Parameter	Value	Δ	δ
A_1[kCnts/Chnl]	8,0091	±0,0096	0,1%
τ_1[ns]	42 075	±35	0,1%
I_1[kCnts]	10 530,7	±7,1	0,1%
A_{Rel1}[%]	90,6	±0,5	0,5%
I_{Rel1}[%]	99,4	±0,1	0,0%
A_2[kCnts/Chnl]	0,832	±0,044	5,3%
τ_2[ns]	2 790	±280	9,9%
I_2[kCnts]	72,4	±5,0	6,9%
A_{Rel2}[%]	9,5	±0,5	4,7%
I_{Rel2}[%]	0,7	±0,1	6,9%
$Bkgr_{Dec}$[kCnts]	0,0010	±0,0002	16%
τ_{AvInt}[ns]	41 807	±28	0,1%
τ_{AvAmp}[ns]	38 380	±210	0,5%

Figure S-81. Left: Raw (experimental) time-resolved photoluminescence decay of **Re(L$_{Me\text{-}nHo}$)Py** in liquid DCM at 298 K (Ar-purged), including the residuals (λ_{exc} = 376.7 nm, λ_{em} = 495 nm). Right: Fitting parameters including pre-exponential factors and confidence limits.

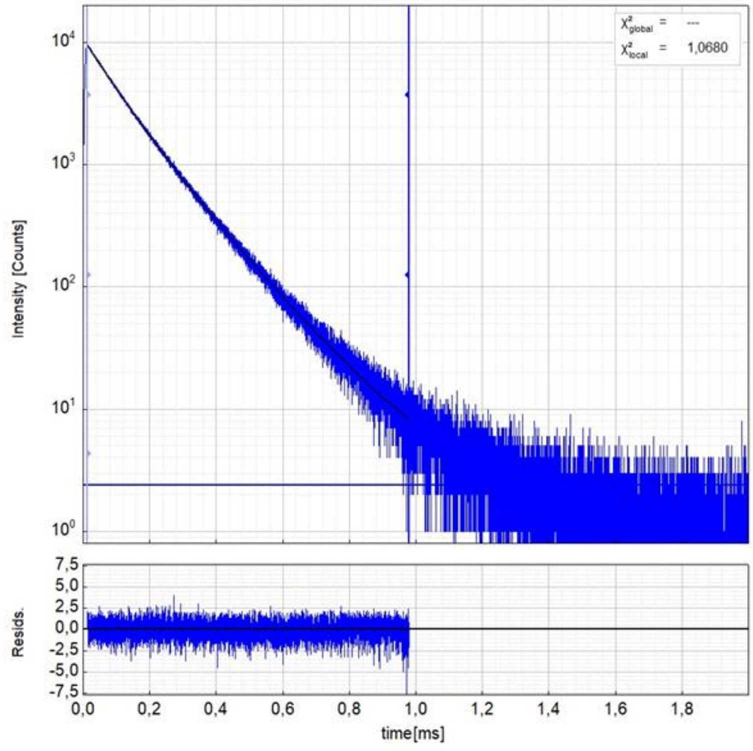

Parameter	Value	Δ	δ
A_1[kCnts/Chnl]	3,88	±0,24	6,2%
τ_1[ns]	148 800	±1 900	1,3%
I_1[kCnts]	4 500	±230	4,9%
A_{Rel1}[%]	41,1	±2,5	6,0%
I_{Rel1}[%]	54,9	±2,7	4,9%
A_2[kCnts/Chnl]	5,56	±0,23	4,1%
τ_2[ns]	85 500	±1 600	1,9%
I_2[kCnts]	3 710	±230	6,0%
A_{Rel2}[%]	59,0	±2,5	4,2%
I_{Rel2}[%]	45,2	±2,7	6,0%
$Bkgr_{Dec}$[kCnts]	0,0024	---	---
τ_{AvInt}[ns]	120 140	±110	0,1%
τ_{AvAmp}[ns]	111 430	±180	0,2%

Figure S-82. Left: Raw (experimental) time-resolved photoluminescence decay of **Re(L$_{Me\text{-}nHo}$)Py** in a frozen glassy matrix of DCM/MeOH (V:V = 1:1) at 77 K, including the residuals (λ_{exc} = 376.7 nm, λ_{em} = 476 nm). Right: Fitting parameters including pre-exponential factors and confidence limits.

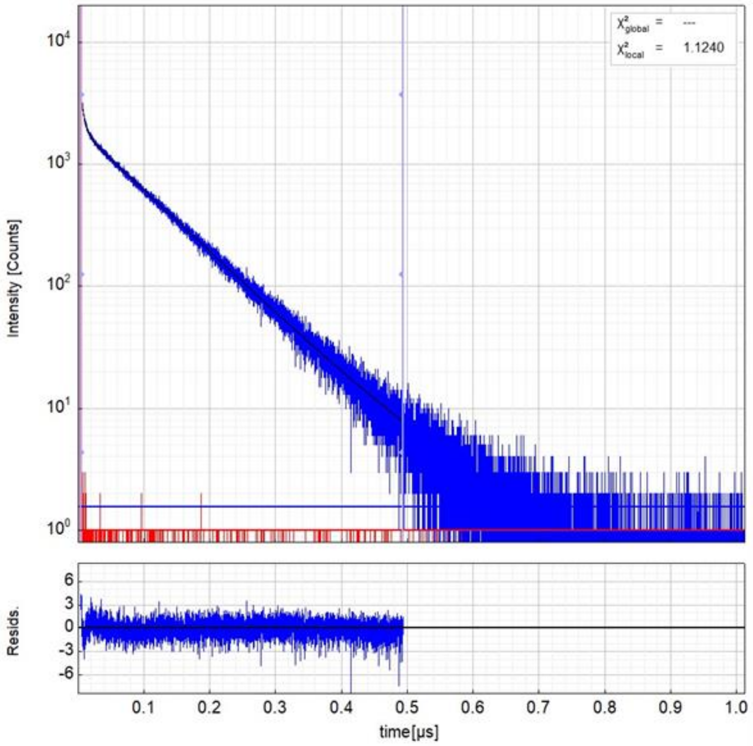

Parameter	Value	Δ	δ
A_1[kCnts/Chnl]	1.9171	±0.0064	0.3%
τ_1[ns]	85.88	±0.22	0.3%
I_1[kCnts]	2 572.3	±4.3	0.2%
A_{Rel1}[%]	39.1	±1.2	3.0%
I_{Rel1}[%]	92.0	±0.2	0.1%
A_2[kCnts/Chnl]	2.99	±0.16	5.1%
τ_2[ns]	4.82	±0.20	4.1%
I_2[kCnts]	225.1	±4.0	1.8%
A_{Rel2}[%]	61.0	±1.2	1.9%
I_{Rel2}[%]	8.1	±0.2	1.7%
$Bkgr_{Dec}$[kCnts]	0.0016	±0.0003	15%
$Bkgr_{IRF}$[Cnts/Chnl]	1.0000	---	---
$Shift_{IRF}$[ps]	123.5096	---	---
τ_{AvInt}[ns]	79.36	±0.23	0.3%
τ_{AvAmp}[ns]	36.5	±1.2	3.0%

Figure S-83. Left: Raw (experimental) time-resolved photoluminescence decay of **Re(L$_{Me-nHo}$)PTA** in liquid acetonitrile at 298 K (air-equilibrated, blue), including the residuals (λ_{exc} = 376.7 nm, λ_{em} = 490 nm) and the instrument response function (IRF, red). Right: Fitting parameters including pre-exponential factors and confidence limits.

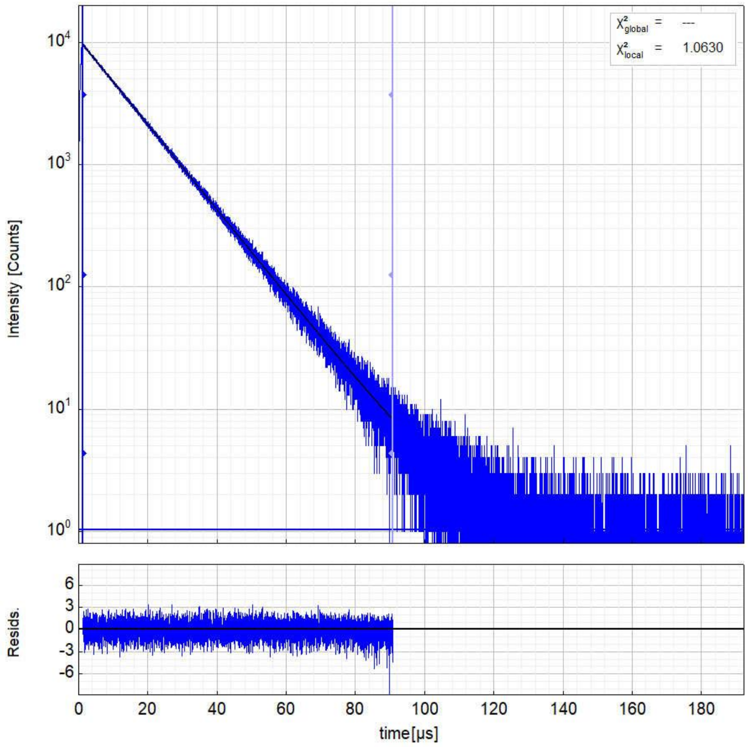

Parameter	Value	Δ	δ
A_1[kCnts/Chnl]	9.5394	±0.0035	0.0%
τ_1[ns]	12 458.3	±5.4	0.0%
I_1[kCnts]	14 855.6	±5.0	0.0%
A_{Rel1}[%]	100.0	---	---
I_{Rel1}[%]	100.0	---	---
$Bkgr_{Dec}$[kCnts]	0.0011	±0.0002	13%
τ_{AvInt}[ns]	12 458.3	±5.4	0.0%
τ_{AvAmp}[ns]	12 458.3	±5.4	0.0%

Figure S-84. Left: Raw (experimental) time-resolved photoluminescence decay of **Re(L$_{Me-nHo}$)PTA** in liquid acetonitrile at 298 K (Ar-purged), including the residuals (λ_{exc} = 376.7 nm, λ_{em} = 487 nm). Right: Fitting parameters including pre-exponential factors and confidence limits.

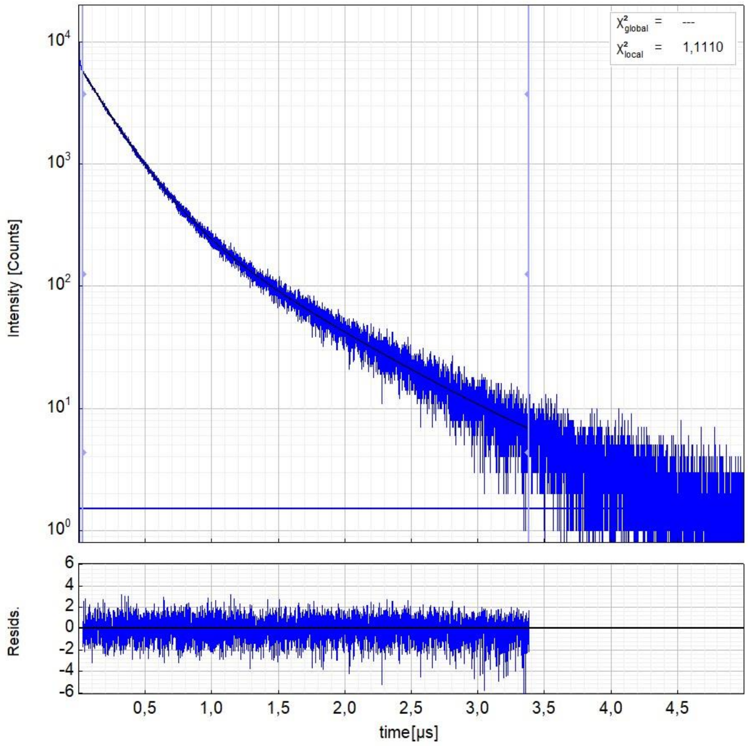

Parameter	Value	Δ	δ
A_1[kCnts/Chnl]	0,701	±0,020	2,8%
τ_1[ns]	686,6	±6,4	0,9%
I_1[kCnts]	962	±19	1,9%
A_{Rel1}[%]	12,4	±0,4	2,6%
I_{Rel1}[%]	30,0	±0,6	1,9%
A_2[kCnts/Chnl]	4,963	±0,012	0,2%
τ_2[ns]	226,8	±1,5	0,6%
I_2[kCnts]	2 251	±19	0,8%
A_{Rel2}[%]	87,7	±0,4	0,4%
I_{Rel2}[%]	70,1	±0,6	0,8%
$Bkgr_{Dec}$[kCnts]	0,0015	---	---
τ_{AvInt}[ns]	364,39	±0,41	0,1%
τ_{AvAmp}[ns]	283,57	±0,59	0,2%

Figure S-85. Left: Raw (experimental) time-resolved photoluminescence decay of **Re(L$_{Me-nHo}$)PTA** in liquid DCM at 298 K (air-equilibrated), including the residuals (λ_{exc} = 376.7 nm, λ_{em} = 490 nm). Right: Fitting parameters including pre-exponential factors and confidence limits.

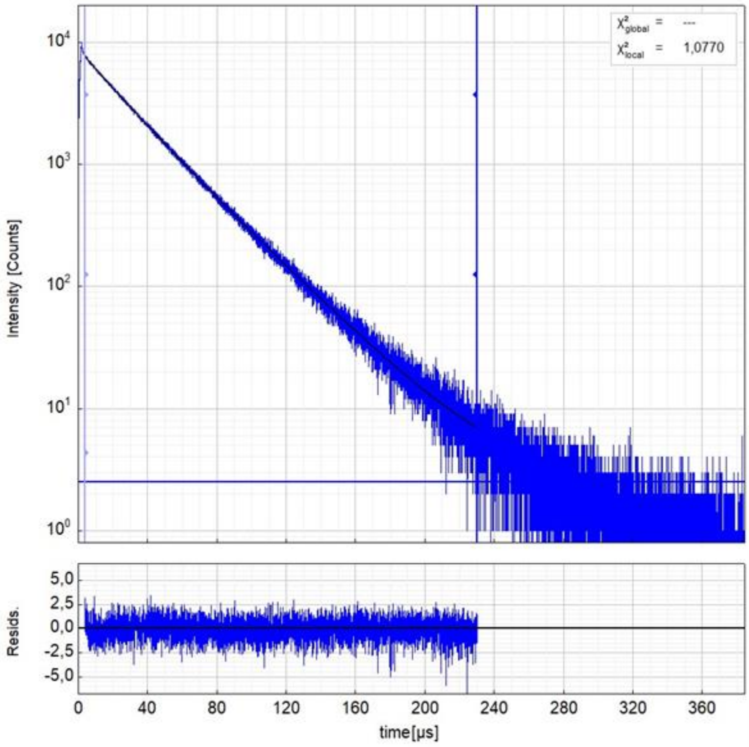

Parameter	Value	Δ	δ
A_1[kCnts/Chnl]	5,45	±0,29	5,3%
τ_1[ns]	31 830	±330	1,0%
I_1[kCnts]	5 420	±230	4,2%
A_{Rel1}[%]	71,0	±3,7	5,2%
I_{Rel1}[%]	80,3	±3,5	4,3%
A_2[kCnts/Chnl]	2,24	±0,29	13%
τ_2[ns]	19 080	±870	4,6%
I_2[kCnts]	1 340	±240	17%
A_{Rel2}[%]	29,1	±3,7	13%
I_{Rel2}[%]	19,8	±3,5	17%
$Bkgr_{Dec}$[kCnts]	0,0025	---	---
τ_{AvInt}[ns]	29 309	±26	0,1%
τ_{AvAmp}[ns]	28 117	±51	0,2%

Figure S-86. Left: Raw (experimental) time-resolved photoluminescence decay of **Re(L$_{Me-nHo}$)PTA** in liquid DCM at 298 K (Ar-purged), including the residuals (λ_{exc} = 376.7 nm, λ_{em} = 488 nm). Right: Fitting parameters including pre-exponential factors and confidence limits.

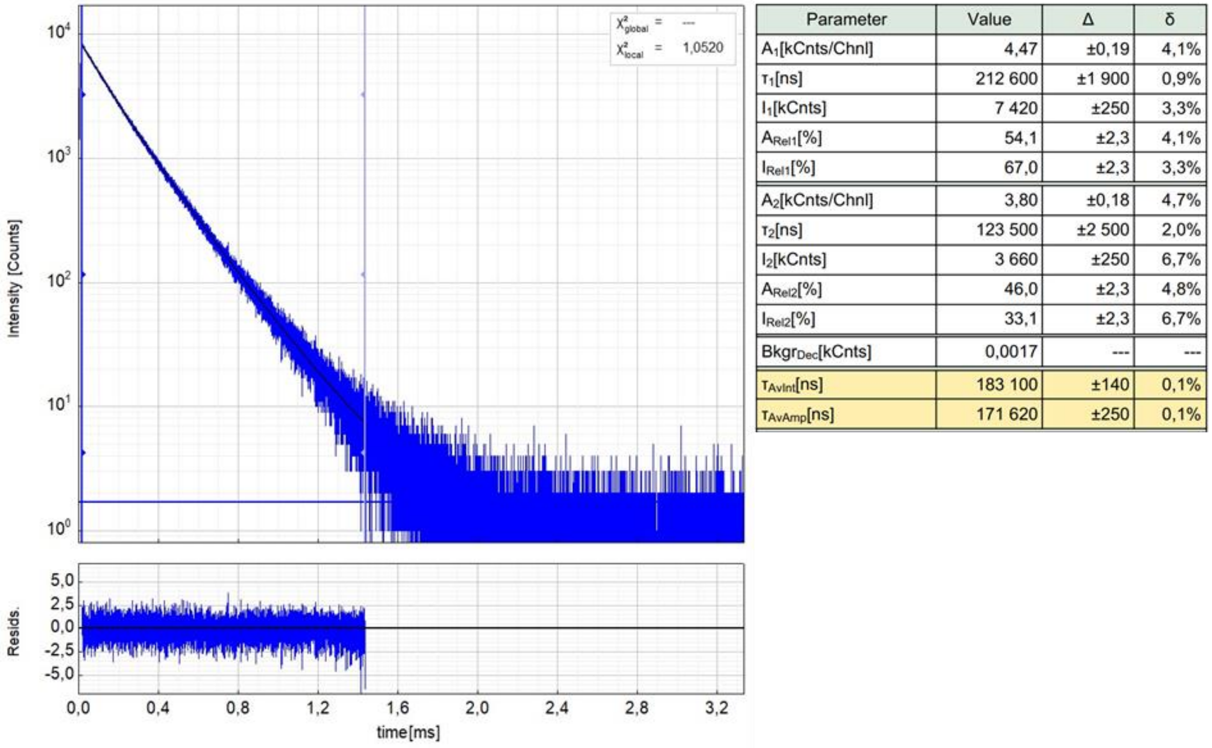

Parameter	Value	Δ	δ
A_1[kCnts/Chnl]	4,47	±0,19	4,1%
τ_1[ns]	212 600	±1 900	0,9%
I_1[kCnts]	7 420	±250	3,3%
A_{Rel1}[%]	54,1	±2,3	4,1%
I_{Rel1}[%]	67,0	±2,3	3,3%
A_2[kCnts/Chnl]	3,80	±0,18	4,7%
τ_2[ns]	123 500	±2 500	2,0%
I_2[kCnts]	3 660	±250	6,7%
A_{Rel2}[%]	46,0	±2,3	4,8%
I_{Rel2}[%]	33,1	±2,3	6,7%
$Bkgr_{Dec}$[kCnts]	0,0017	---	---
τ_{AvInt}[ns]	183 100	±140	0,1%
τ_{AvAmp}[ns]	171 620	±250	0,1%

Figure S-87. Left: Raw (experimental) time-resolved photoluminescence decay of **Re(L$_{Me\text{-}nHo}$)PTA** in a frozen glassy matrix of DCM/MeOH (V:V = 1:1) at 77 K, including the residuals (λ_{exc} = 376.7 nm, λ_{em} = 473 nm). Right: Fitting parameters including pre-exponential factors and confidence limits.

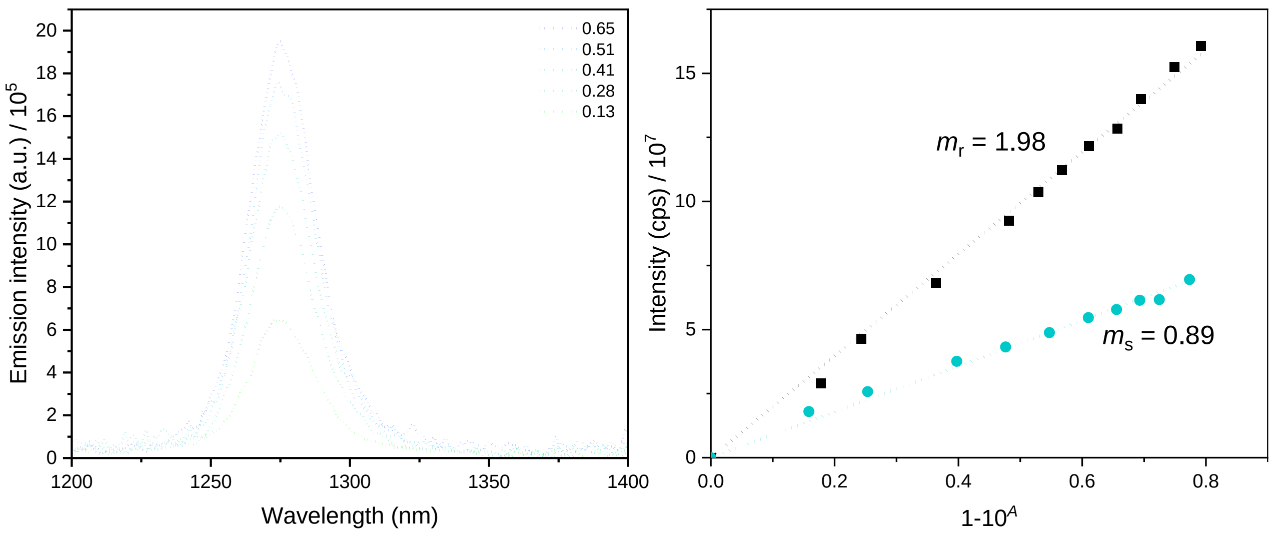

Figure S-88. Singlet **di**oxygen photogeneration from **Re(L$_{nHo}$)Py** in acetonitrile. Left: $^1O_2$ phosphorescence spectra at different concentrations of the complex. Right $^1O_2$ phosphorescence intensity *vs*. 1-10^A for **Re(L$_{nHo}$)Py** (cyan) and for the reference (black). λ_{exc} = 350 nm.

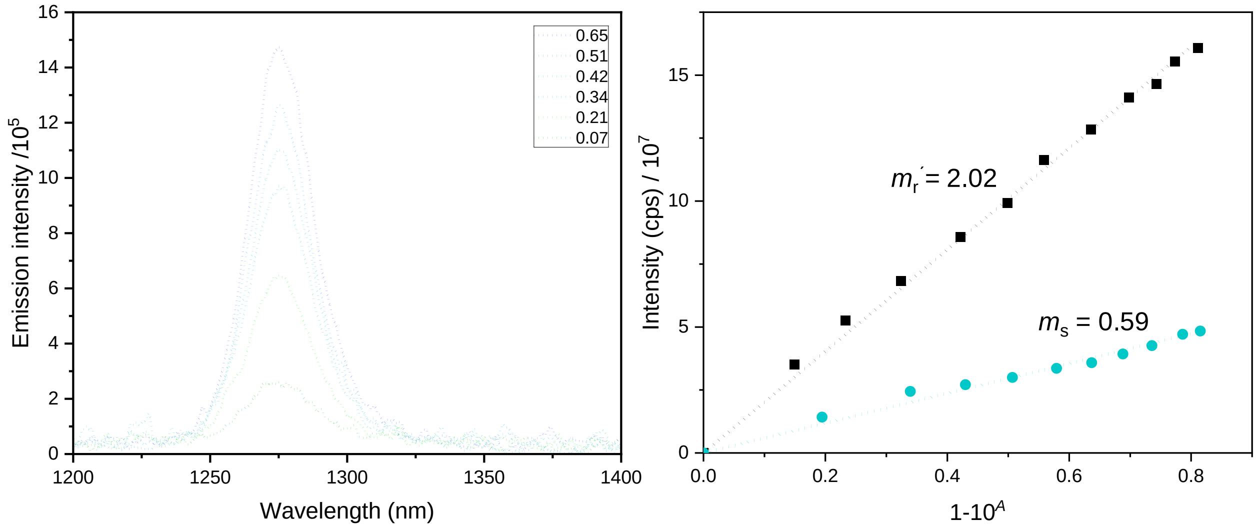

Figure S-89. Singlet **di**oxygen photogeneration from **Re(L$_{nHo}$)PTA** in acetonitrile. Left: $^1O_2$ phosphorescence spectra at different concentrations of the complex. Right $^1O_2$ phosphorescence intensity *vs*. 1-10^A for **Re(L$_{nHo}$)PTA** (cyan) and for the reference (black). λ_{exc} = 350 nm.

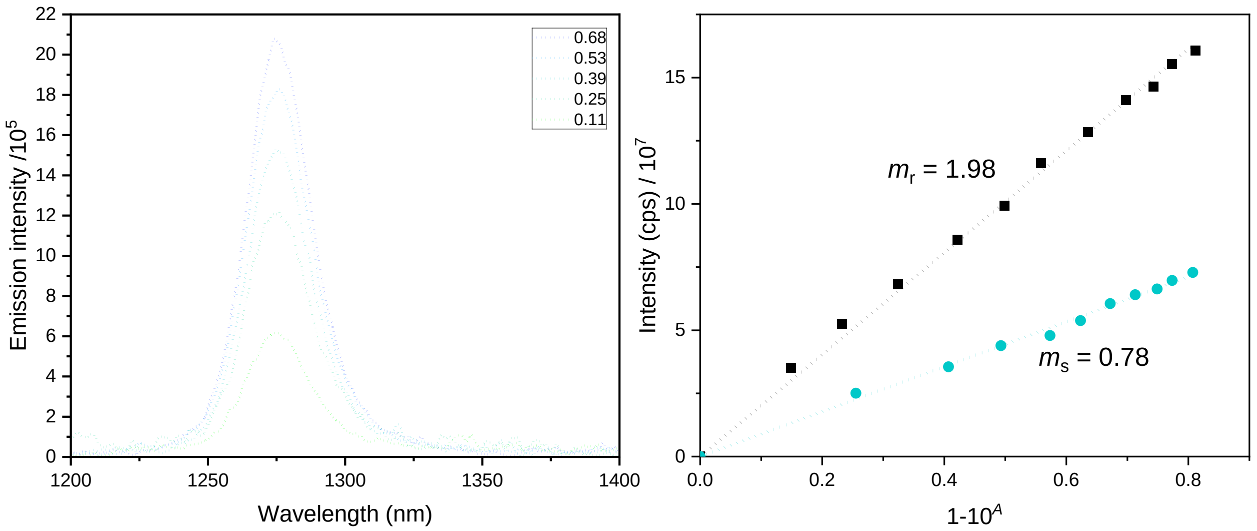

Figure S-90. Singlet **di**oxygen photogeneration from **Re($L_{Me\text{-}nHo}$)Py** in acetonitrile. Left: $^1O_2$ phosphorescence spectra at different concentrations of the complex. Right $^1O_2$ phosphorescence intensity *vs*. $1\text{-}10^A$ for **Re($L_{Me\text{-}nHo}$)Py** (cyan) and for the reference (black). λ_{exc} = 350 nm.

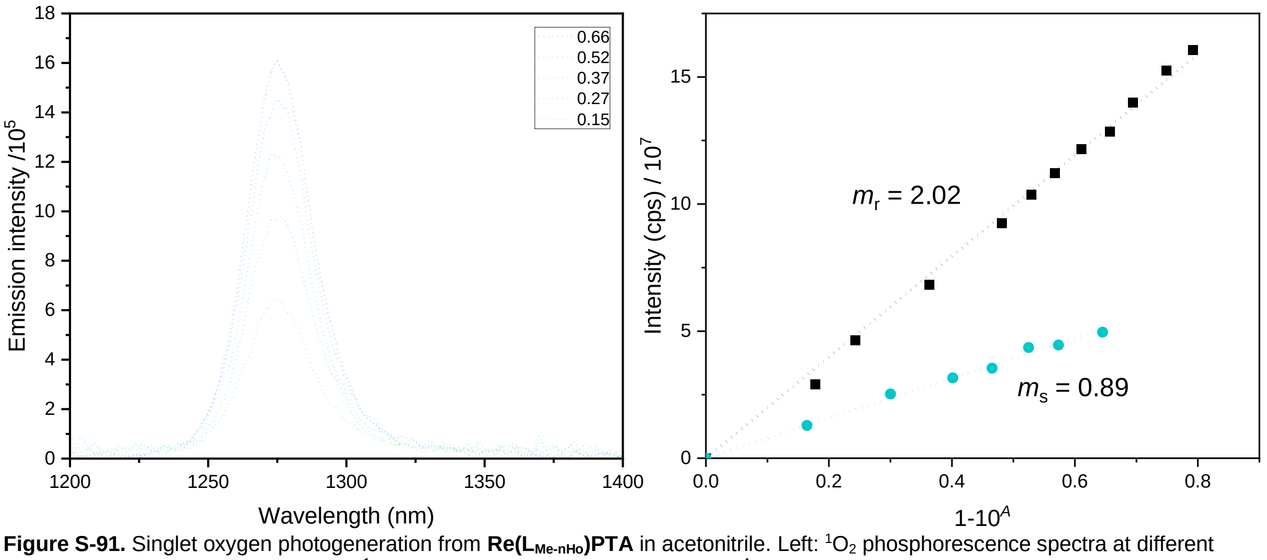

Figure S-91. Singlet oxygen photogeneration from **Re($L_{Me\text{-}nHo}$)PTA** in acetonitrile. Left: $^1O_2$ phosphorescence spectra at different concentrations of the complex. Right $^1O_2$ phosphorescence intensity *vs*. $1\text{-}10^A$ for **Re($L_{Me\text{-}nHo}$)PTA** (cyan) and for the reference (black). λ_{exc} = 350 nm.

Equations used to calculate the parameters of singlet dioxygen photogeneration and the corresponding uncertainties by the total differential method:

$$P_{\mathrm{O_2}}^{\mathrm{T}} = 1 - \frac{\tau_{\mathrm{air}}}{\tau_{\mathrm{Ar}}} \qquad \Delta P_{\mathrm{O_2}}^{\mathrm{T}} = \frac{\tau_{\mathrm{air}}\Delta\tau_{\mathrm{Ar}}}{(\tau_{\mathrm{Ar}})^2} + \frac{\Delta\tau_{\mathrm{air}}}{\tau_{\mathrm{Ar}}}$$

$$f_{\mathrm{O_2}}^{\mathrm{T}} = \frac{\Phi_{\Delta}^{\mathrm{s}}}{P_{\mathrm{O_2}}^{\mathrm{T}}} \qquad \Delta f_{\mathrm{O_2}}^{\mathrm{T}} = \frac{\Phi_{\Delta}^{\mathrm{s}}\Delta P_{\mathrm{O_2}}^{\mathrm{T}}}{(P_{\mathrm{O_2}}^{\mathrm{T}})^2} + \frac{\Delta\Phi_{\Delta}^{\mathrm{s}}}{P_{\mathrm{O_2}}^{\mathrm{T}}}$$

$$k_{\mathrm{q}} = \frac{\tau_{\mathrm{Ar}} - \tau_{\mathrm{air}}}{\tau_{\mathrm{Ar}}\tau_{\mathrm{air}}[\mathrm{O_2}]} \qquad \Delta k_{\mathrm{q}} = \frac{\Delta\tau_{\mathrm{air}}}{[\mathrm{O_2}](\tau_{\mathrm{air}})^2} + \frac{\Delta\tau_{\mathrm{Ar}}}{[\mathrm{O_2}](\tau_{\mathrm{Ar}})^2} + \frac{\Delta[\mathrm{O_2}](\tau_{\mathrm{Ar}} - \tau_{\mathrm{air}})}{\tau_{\mathrm{Ar}}\tau_{\mathrm{air}}[\mathrm{O_2}]}$$ (assuming that $\Delta[O_2] = 0.1[O_2]$)

$$\Phi_{\Delta}^{\mathrm{s}} = \Phi_{\Delta}^{\mathrm{r}} \cdot \frac{\mathrm{m_s}}{\mathrm{m_r}} \qquad \Delta\Phi_{\Delta}^{\mathrm{s}} = \frac{\mathrm{m_s}}{\mathrm{m_r}}\Delta\Phi_{\Delta}^{\mathrm{r}} + \frac{\Phi_{\Delta}^{\mathrm{r}}}{\mathrm{m_r}}\Delta\mathrm{m_s} + \frac{\Phi_{\Delta}^{\mathrm{r}}}{\mathrm{m_r^2}}\Delta\mathrm{m_r}$$

Section S5: Reference

[1] Q.-M. Jin, Y. Lu, J.-L. Jin, H. Guo, G.-W. Lin, Y. Wang, T. Lu, Synthesis, characterization, DNA binding ability and cytotoxicity of the novel platinum(II), copper(II), cobalt(II) and nickel(II) complexes with 3-(1H-benzo[d]imidazol-2-yl)-*β*-carboline. *Inorg. Chim. Acta* **2014**, *421*, 91.
